# Identification of Novel and Potent Indole-Based Benzenesulfonamides as Selective Human Carbonic Anhydrase II Inhibitors: Design, Synthesis, In Vitro, and In Silico Studies

**DOI:** 10.3390/ijms23052540

**Published:** 2022-02-25

**Authors:** Ahmed Elkamhawy, Jiyu Woo, Hossam Nada, Andrea Angeli, Tarek M. Bedair, Claudiu T. Supuran, Kyeong Lee

**Affiliations:** 1 BK21 FOUR Team and Integrated Research Institute for Drug Development, College of Pharmacy, Dongguk University-Seoul, Goyang 10326, Korea; a_elkamhawy@mans.edu.eg (A.E.); wju2757@dgu.ac.kr (J.W.); hossam_hammouda@dgu.ac.kr (H.N.); 2 Department of Pharmaceutical Organic Chemistry, Faculty of Pharmacy, Mansoura University, Mansoura 35516, Egypt; 3 Pharmaceutical Chemistry Department, Faculty of Pharmacy, Badr University, Cairo 11829, Egypt; 4 NEUROFARBA Department, Sezione di Scienze Farmaceutiche, University of Florence, Via Ugo Schiff 6, Sesto Fiorentino, 50019 Florence, Italy; andrea.angeli@unifi.it; 5 Chemistry Department, Faculty of Science, Minia University, Minia 61519, Egypt; dr.tarek.bedair.2@gmail.com

**Keywords:** indole based-benzenesulfonamide, amide coupling, carbonic anhydrase inhibitors, molecular docking, structure–activity relationship (SAR)

## Abstract

In recent decades, human carbonic anhydrase inhibitors (hCAIs) have emerged as an important therapeutic class with various applications including antiglaucoma, anticonvulsants, and anticancer agents. Herein, a novel series of indole-based benzenesulfonamides were designed, synthesized, and biologically evaluated as potential hCAIs. A regioisomerism of the sulfonamide moiety was carried out to afford a total of fifteen indole-based benzenesulfonamides possessing different amide linkers that enable the ligands to be flexible and develop potential H-bond interaction(s) with the target protein. The activity of the synthesized compounds was evaluated against four hCA isoforms (I, II, IX and, XII). Compounds **2b**, **2c**, **2d**, **2f**, **2h** and **2o** exhibited potent and selective profiles over the hCA II isoform with K_i_ values of 7.3, 9.0, 7.1, 16.0, 8.6 and 7.5 nM, respectively. Among all, compound **2a** demonstrated the most potent inhibition against the hCA II isoform with an inhibitory constant (K_i_) of 5.9 nM, with 13-, 34-, and 9-fold selectivity for hCA II over I, IX and XII isoforms, respectively. Structure–activity relationship data attained for various substitutions were rationalized. Furthermore, a molecular docking study gave insights into both inhibitory activity and selectivity of the target compounds. Accordingly, this report presents a successful scaffold hoping approach that reveals compound **2a** as a highly potent and selective indole-based hCA II inhibitor worthy of further investigation.

## 1. Introduction

Carbonic anhydrases (CAs) are metalloenzymes found in all living things that are required for the conversion of CO_2_ to bicarbonate and protons [1]. CAs observed in humans belong to the α-class (α-CA), and are classified into sixteen isoforms that vary in molecular characteristics, oligomeric structure, cellular localization, distribution in organs and tissues, expression levels, and kinetic properties [2]. The distribution pattern of the CA isoforms can be described as, cytosolic (I, II, III, VII, and XIII), membrane (IV, IX, XII, and XIV), secretory (VI), and mitochondrial (VA and VB) forms [3,4]. Carbonic anhydrases have a role in a variety of physiological processes, including respiration, pH control, ion transport, bone resorption, and gastric fluid secretion. As a result, inhibitors of these enzymes have become an important therapeutic class in recent decades. Topically acting antiglaucoma, anticonvulsants, and anticancer agents are some of the novel applications of CA inhibitors that have been developed [2,5]. Among the CA isoforms, CA II is widely associated with various types of cancers, with a recent study finding that it is expressed in the endothelium of neovessels in melanoma, esophageal, renal, and lung cancers [6]. Other studies associate CA II with the key target antigen stimulation of an autoantibody response in melanoma patients [7]. The most common approach in designing selective CA inhibitors has been through the modulation of the ring directly linked to the zinc-binding group (ZBG) or by appending different tails to the aromatic ring [8,9]. 

**Figure 1:**
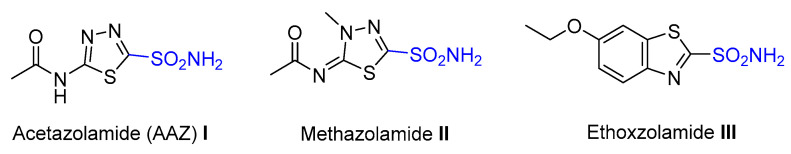
Chemical structures of some clinically used sulfonamide-containing drugs.

One of the most prominent ZBG moieties with carbonic anhydrase inhibitory activity are sulfonamides [10]. Among the investigated sulfonamides, aromatic sulfonamides have been shown to be specific and potent inhibitors of CA isoforms, and they have been considered as potential candidates for the further development and enhancement of more potent inhibitors [11]. Conversely, due to the structural similarity and identical subcellular location of the CA isoforms, carbonic anhydrase inhibitors suffer from a lack of selectivity which often results in unwanted side effects [12]. When designing inhibitors for the metalloenzymes carbonic anhydrases, sulfonamides are regarded as one of the most effective ZBGs among the studied sulfonamides. This is due to their structural features that are ideal for binding to the Zn^2+^ ion in the active site of the receptor [13,14,15]. The negatively charged nitrogen of sulfonamide coordinates the positively charged metal ion [9,16]. Accordingly, the primary sulfonamide group (SO_2_NH_2_) plays an important role in CA inhibition mechanism, with more than 20 compounds in clinical use including acetazolamide (AAZ) **I**, methazolamide **II**, and ethoxzolamide **III** (Figure 1).

**Figure 2:**
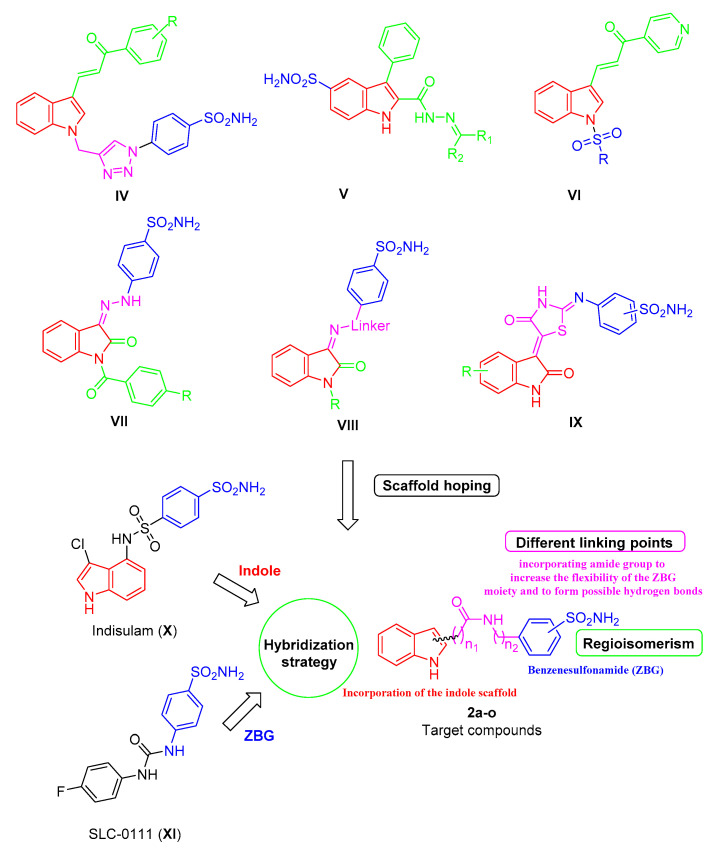
Design strategy of the synthesized hybrids **2a**–**o**.

The indole scaffold has been identified to afford highly potent carbonic anhydrase inhibitors. A recent series of indole-1,2,3-triazole chalcone hybrids was synthesized by click chemistry as CA inhibitors (general structure **IV**, Figure 2). Although some compounds of this series were able to successfully inhibit the cytosolic isoform hCA I in a low nanomolar range of potency (18.8–50.4 nM), they did not show a similar potency over the tumor-associated hCA II isoform. The tumor associated isoform hCA IX was also weakly inhibited by all the synthesized compounds with K_i_ values ranging from 69.3 nM to 1.4 μM [17]. Another series of sulfonamidoindole-based hydrazones possessing a 2-(hydrazinocarbonyl)-3-phenyl-1*H*-indole-5-sulfonamide scaffold (general structure **V**, Figure 2) was reported with CA inhibitory activity for the tumor-associated carbonic anhydrase isoforms, hCA IX and XII. Despite the high potency of these derivatives against hCA IX and XII, they displayed weak inhibitory activity against both hCA I isoform (K_i_s range: from 3.13 μM to >10.00 μM) and hCA II isoform (K_i_s range: from 309 nM to >10.00 μM) [18]. Peerzeeda et al. reported a series of sulfonamide derivatives of pyridyl-indole-based chalcone (general structure **VI**, Figure 2) as hCA IX inhibitors with antitumor activities against MCF-7 andHepG-2 cell lines [19]. *N*-aroyl-2-oxindole benzenesulfonamide conjugates (general structure **VII**, Figure 2) were also reported by George et al. to preferentially inhibit the tumor-associated isoforms IX and XII [20]. The same research group reported another series of 2-oxindole benzenesulfonamide conjugates (general structure **VIII**, Figure 2), with many compounds showing promising activity and selectivity toward CAI, II and IX compared to AAZ (**I**) [20]. Novel 4/3-((4-oxo-5-(2-oxoindolin-3-ylidene)thiazolidin-2-ylidene)amino)benzenesulfonamides (general structure **IX**, Figure 2) were also recently reported as CA inhibitors. Isoforms II and IX (tumor-associated) were found to be more susceptible to inhibition by this series, with K_i_s in the range of 2.6–598.2 nM for hCA II, and of 16.1–321 nM for hCA IX. Regarding clinical trials, indisulam (**X**, Figure 2) is one of these indole-based benzenesulfonamide anticancer agents. It was in phase II clinical studies until recently, and it inhibits several CA isoforms, including the tumor-associated isoform IX [21]. Moreover, another example in clinical trials is SLC-0111 (**XI**), a benzenesulfonamide compound currently in phase I/II clinical trials for the treatment of advanced metastatic solid tumors [22].

Inspired by all these efforts related to the development of indole-based hCA inhibitors, we designed a novel series of indole-based benzenesulfonamides (Figure 2) through a hybridization process of these two scaffolds (indole and benzenesulfonamide). Thereafter, a regioisomerism of the ZBG moiety was carried out to afford fifteen indole-based benzenesulfonamides linked together via an amide linker, giving the synthesized compounds flexibility as well as the ability to form hydrogen bond interactions with the target protein. To the best of our knowledge, this hybridization approach of the indole moiety with the most effective ZBG moiety (the benzenesulfonamide group) via a flexible amide linker on different positions on the indole core to develop novel small molecules with a promising potential to inhibit carbonic anhydrase isoforms has not been reported so far.

## 2. Results and Discussion

### 2.1. Chemical Synthesis

**Scheme 1:**
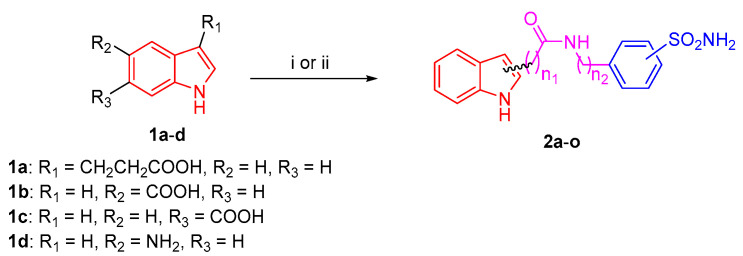
Reagent: appropriate benzenesulfonamide reagent (see Table 1). Conditions: (i) EDCI, HOBt, DIPEA, acetonitrile or DMF/DCM (5/1), rt, 12 h, or (ii) T3P, DIPEA, THF, rt, 12 h.

As shown in Scheme 1, a series of fifteen benzenesulfonamide analogues (**2a**–**o**) were synthesized via a coupling reaction of amide synthesis. The reaction was carried out utilizing a range of commercially available appropriate indole-based starting materials; 3-(1*H*-indol-3-yl)propanoic acid (**1a**), 1*H*-indole-5-carboxylic acid (**1b**), 1*H*-indole-6-carboxylic acid (**1c**), or 1*H*-indol-5-amine (**1d**). Using coupling catalysts such as EDCI, HOBt, or T3P, several benzenesulfonamide reagents were allowed to react overnight at room temperature with the starting materials **1a**–**d** (as described in Table 1) in a suitable solvent such as MeCN, DMF/DCM (5/1), or THF. As a result, a wide range of indole-based benzenesulfonamide analogues (**2a**–**o**) with diverse substituents were afforded in acceptable yields (Table 1). 

Different techniques were utilized to elucidate the chemical structures and the purity of the synthesized target compounds. The ^1^H NMR spectra of compounds **2a**–**o** were characterized by the appearance of two peaks at 7.20–7.50 ppm attributable to the sulfonamide NH_2_ and another peak at 10.20–10.80 ppm attributable to the NH of the amide group confirming the formation of compounds **2a**–**o**. Compound **2a** synthesis was further confirmed by the appearance of two triplet peaks in the aliphatic region at 3.05–3.01 and 2.73–2.69 ppm attributable to the two CH_2_ groups of the aliphatic linker moiety. Similarly, compound **2b** synthesis was easily confirmed by the appearance of the two triplet peaks attributable to the two CH_2_ groups, as well as a singlet peak at 10.77 ppm attributable to the OH substituent on the ZBG moiety. Compound **2c** was characterized by the presence of four triplet peaks at 3.29–3.26, 2.90–2.88, 2.76–2.74 and 2.43–2.39 attributable to the four CH_2_ groups of the aliphatic linker moiety. In the same manner, the presence of triplet peaks in the aliphatic range of the ^1^H NMR spectrum associated with the presence of CH_2_ groups in the linker moiety was used to confirm the synthesis of compounds **2h** and **2m**.

### 2.2. Carbonic Anhydrase Inhibition

The CA inhibitory activities of the hybrid compounds **2a**–**o** and AAZ, as a standard inhibitor, were measured against four isoforms of hCA by a stopped flow CO_2_ hydrase assay (Table 2). The tested CA panel was comprised of the two ubiquitously expressed cytosolic hCA I and II isoforms, along with the two tumor-related transmembrane hCA IX and XII isoforms. The activity of the synthesized hybrids was reported as K_i_ (inhibition constant) to assess their binding affinity. Among the synthesized hybrids, compounds **2a**, **2d** and **2o** exhibited potent inhibitory activities against the hCA I isoform with inhibition constants (K_i_) of 79.8, 97.6 and 56.6 nM, respectively, compared to AAZ (250 nM). Compound **2a** demonstrated the most potent inhibition among all compounds against the hCA II isoform with an inhibition constant of 5.9 nM. This result indicated that compound **2a** possessed 13-fold selectivity for hCA II over the hCA I isoform and 34- and 9-fold selectivity for hCA II over the hCA IX and XII isoforms, respectively. Similarly, compounds **2b**, **2c**, **2d**, **2f**, **2h** and **2o** exhibited potent and selective activity over the hCA II isoform with inhibition constant values of 7.3, 9.0, 7.1, 16.0, 8.6 and 7.5 nM, respectively. On the contrary, the synthesized hybrids only displayed moderate inhibitory activity over the hCA IX isoform with inhibition constant ranging from 169.6–766.3 nM. All the synthesized hybrids displayed potent activity against the tumor-related hCA XII isoform with the most potent hybrids being compounds **2c** and **2n**, which exhibited an inhibition constant of 36.9 nM. Except for compounds **2f** and **2h** which possessed moderate hCA XII inhibition constant of 257.0 and 235.6, respectively, the remaining synthesized hybrids displayed double-digit nanomolar activity ranging from 40.5–90.9 nM. The inhibitory activity of the synthesized hybrids over the hCA isoforms I, II, IX and XII is demonstrated in Table 2.

**Figure 3:**
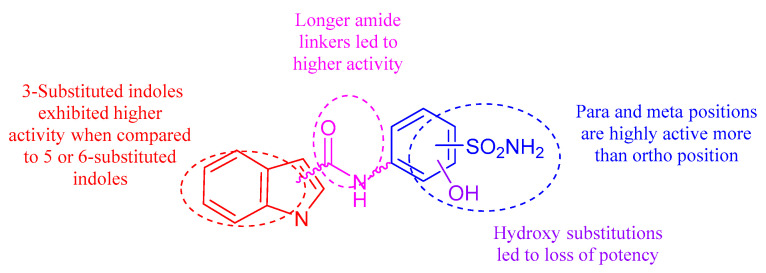
Structure activity relationship (SAR) diagram of the synthesized hybrids **2a**–**o**.

Generally, the sulfonamides substituted at the para- and meta-positions generally exhibited higher inhibitory activity when compared to the ortho-substituted sulfonamide-indole hybrids. Hydroxy substitutions in compounds **2g** and **2l** led to a loss of the potent inhibitory activity. However, the hydroxy substituted compound **2b** displayed a potent inhibitory activity on hCA II (K_i_ = 7.3 nM) and XII (K_i_ = 85.1 nM), which could be attributed to the presence of a longer amide linker. Similarly, in all the synthesized compounds, the longer amide linker usually led to higher activity when compared to a shorter one. Moreover, 3-substituted indoles (**2a**, **2b**, **2c** and **2d**) exhibited the highest activity when compared to 5 or 6-substituted indoles. Interestingly, the reverse amide **2o** showed potent inhibitory activities against all hCA isoforms making it a potential lead for a new series of CA inhibitors. All the synthesized compounds showed potent activity against the tumor-related hCA XII with inhibitory activity ranging from 36.9–257.0 nM. A structure activity relationship (SAR) diagram of the synthesized hybrids **2a**–**o** is illustrated in Figure 3.

### 2.3. Molecular Docking Study

**Figure 4:**
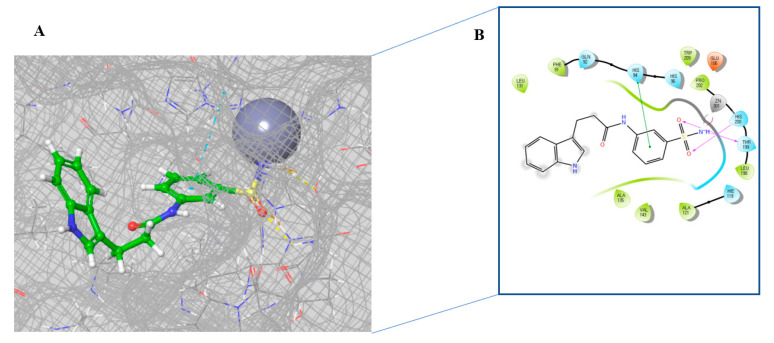
The interaction patterns of compound **2a** in the active sites of hCAI. (**A**) 3D model of compound **2a** surface CA active site, with the surface viewed in the mesh format. (**B**) 2D interaction pattern of compound **2a** with hCA I active site.

The prediction of the binding modes between a ligand and its intended target, as well as the correlation of the obtained scores with prospective activity, are all different applications of molecular docking in computer-aided drug design research [23]. The ability to estimate the impact of specific amino acid mutations on the ligand’s activity profile is another utility of molecular docking studies [24]. Additionally, visualizing the docking study’s resulting interactions aids future ligand modification, resulting in molecules with better affinity properties [25]. Accordingly, a molecular docking study for synthesized hybrid compounds was performed for hCA isozymes I, II and IX to correlate the structural characteristics with the inhibitory activity and investigate the binding modes of the synthesized hybrids. Docking of the synthesized hybrids revealed that the benzenesulfonamide ring fits deeply inside the shallow CA active site, anchoring the zinc ion via a metal bond with Zn^2+^, which is typical for sulfonamide CAIs. Compound **2a** was selected as a representative ligand to be docked over the different isoforms of CA due to its inhibitory profile, where it displayed a remarkable inhibitory profile over the tested isoforms with inhibition constant values of 79.8, 5.9, 205.8 and 54.2 nM over hCA I, II, IX and XII, respectively. For hCA I, compound **2a** adhered to the common pattern of benzenesulfonamides, where the benzenesulfonamide ring fit deeply inside the surface CA active site through coordination with the zinc moiety in the distinctive manner of sulfonamide CAIs via NH^−^-Zn^2+^ bond (Figure 4A). Additionally, compound **2a** established two hydrogen bonds between the oxygens of the sulfonamide moiety with THR199 and HIS200 amino acid residues, which stabilized the binding orientation of compound **2a** inside the active site cavity (Figure 4B). The presence of the hydrogen bonds and metal coordination bonds, as well as the weak hydrophobic interactions, contributed to the potent inhibition profile exhibited by compound **2a**, as well as indicating a strong affinity to the active site of hCAI.

**Figure 5:**
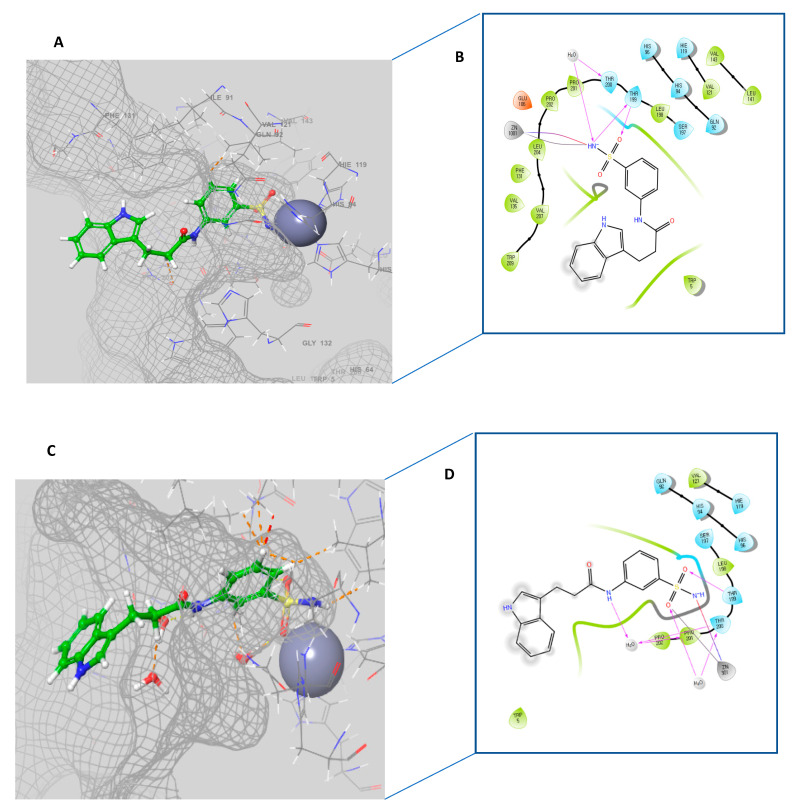
The interaction patterns of compound **2a** in the active sites of hCA II and IX. (**A**) 3D model of compound **2a** surface hCA II active site, with the surface viewed in the mesh format. (**B**) 2D interaction pattern of compound **2a** with hCA II active site. (**C**) 3D model of compound **2a** surface hCA IX active site, with the surface viewed in the mesh format. (**D**) 2D interaction pattern of compound **2a** with hCA IX active site.

Similarly, docking of compound **2a** into the active site of hCA II exhibited the typical metal interaction of the sulfonamide group with the Zn metal (Figure 5B). Compound **2a** established two additional hydrogen bonds with THR 199 amino acid residue of hCA II which increased the stability of the interaction. Furthermore, compound **2a** occupied most of the available space of the shallow active site (Figure 5A) indicating a strong binding affinity between the compound and the active site residues. Docking of compound **2a** into the active site of hCA IX (Figure 5C,D) established two metal interactions between the sulfonamide group and Zn^2+^ in the active site. It also exhibited a similar binding pattern when docked over the different isoforms of hCA, with the flexible amide linker allowing the ligand to fit deeply inside the active site surface to form the essential metal interaction with the Zn^2+^.

## 3. Materials and Methods

### 3.1. Chemistry

The commercial chemicals utilized herein were of reagent grade and were used without further purification. Thermo Scientific 9200 apparatus was employed to measure the melting point of the synthesized compounds. Proton nuclear magnetic resonance (^1^H NMR) spectra were established using a Varian (400 MHz) spectrometer (Varian Medical Systems, Inc., Palo Alto, CA, USA). The ^1^H NMR spectra multiplicity was shown as in the subsequent abbreviations: singlet (s), doublet (d), doublet of doublet (dd), triplet (t), quartet (q), multiplet (m), and broad (b). Chemical shift values are expressed in *δ* values (ppm), while the coupling constant (*J*) values are reported in hertz. ^13^C NMR spectra were obtained employing a Varian (100 MHz) spectrometer and the chemical shifts values are reported in parts per million (ppm) downfield from tetramethylsilane (internal standard) with coupling constants in hertz, as previously reported [26,27]. The G2 QTOF mass spectrometer (Waters Corporation, Milford, MA, USA) was employed to produce the mass spectra, high-resolution mass spectrometry (HRMS, ESI−MS), as previously used [28]. Infrared (IR) spectra were documented as KBr disks utilizing a Shimadzu FT-IR 8400S infrared spectrophotometer. The purification of the products was carried out employing column or flash column chromatography (Biotage, Uppsala, Sweden) with silica gel 60 (230−400 mesh Kieselgel 60), as previously carried out [29]. Moreover, monitoring of the reaction was achieved by employing thin-layer chromatography on 0.25 mm silica plates (E. Merck, silica gel 60 F254). High-performance liquid chromatography (HPLC) analysis was used to assess the optical purity of the synthesized compounds: Chiralpak IG-3 (4.6 × 150, 3), hexane/EtOH/MeOH = 85:10:5, 1.5 mL/min, and λ = 280 nm.

**3-(1*H*-indol-3-yl)-*N*-(3-sulfamoylphenyl)propanamide (2a)**. In a round-bottom flask, the starting material **1a** (3-(1*H*-indol-3-yl)propanoic acid, 0.10 g, 0.50 mmol), 1-ethyl-3-(3-dimethylaminopropyl)carbodiimide (EDCI, 0.14 g, 0.75 mmol) and hydroxybenzotriazole (HOBt, 0.10 g, 0.75 mmol) were dissolved in 8 mL of acetonitrile (MeCN) and stirred at room temperature for 30 min, followed by addition of the 3-aminobenzenesulfonamide (0.09 g, 0.75 mmol). The reaction mixture was stirred at room temperature overnight. After completion of the reaction, excess solvent was evaporated. The obtained residue was diluted with EA, and the organic layer was washed with water and brine. The organic layer was then separated and dried over magnesium sulfate and filtered. The residue was purified by column chromatography on silica gel (0–20% EA:Hex) to obtain the product. White solid. Yield: 35%, mp: 202–204 °C, HPLC purity: 9 min, 99.79%, ^1^H NMR (400 MHz, DMSO-*d*_6_) *δ* 10.78 (s, 1H, indole-NH), 10.24 (s, 1H, amide-NH), 8.18 (s, 1H, Ph-H2′), 7.76–7.74 (m, 1H, Ph-H6′), 7.58–7.56 (d, *J* = 8.00 Hz, 1H, indolyl-H4), 7.49–7.47 (d, *J* = 8.00 Hz, 2H, Ph-H4′ and 5′), 7.35 (s, 2H, NH_2_), 7.34–7.32 (d, 1H, indolyl-H7), 7.13 (s, 1H, indolyl-H2), 7.08–7.04 (t, *J* = 8.00 Hz, 1H, indolyl-H6), 7.00–6.96 (t, *J* = 8.00 Hz, 1H, indolyl-H5), 3.05–3.01 (t, *J* = 8.00 Hz, 2H, CH_2_), 2.73–2.69 (t, *J* = 8.00 Hz, 2H, CH_2_). ^13^C NMR (100 MHz, DMSO-*d*_6_) *δ* 171.81 (C=O amide), 144.98 (Ph-C3′), 140.03 (Ph-C1′), 136.65 (indole-C7a), 129.84 (Ph-C5′), 127.40 (indole-C3a), 122.63 (indole-C2), 122.25 (Ph-C6′), 121.38 (indole-C6), 120.49 (Ph-C4′), 118.78 (Ph-C2′), 118.62 (indole-C5), 116.42 (indole-C4), 113.95 (indole-C3), 111.78 (indole-C7), 37.70 (CH_2_-C=O), 21.17 (indole-CH_2_). HRMS (ESI) *m/z* calculated for C_17_H_17_N_3_O_3_S [M + H]^+^: 344.1069. Found: 344.1087.

***N*-(2-hydroxy-5-sulfamoylphenyl)-3-(1*H*-indol-3-yl)propanamide (2b)**. In a dry round-bottom flask, compound **1a** (0.20 g, 1.06 mmol), propylphosphonic anhydride (T3P, 1.20 mL, 2.10 mmol) and DIPEA (0.55 mL, 3.17 mmol) were dissolved in 8 mL of tetrahydrofuran (THF) and stirred at room temperature for 30 min, followed by the addition of the 3-amino-4-hydroxybenzenesulfonamide (0.24 g, 1.27 mmol). The reaction mixture was stirred at room temperature overnight. After completion of the reaction, excess solvent was evaporated. The obtained residue was diluted with EA, and the organic layer was washed with water and brine. The organic layer was separated and dried over magnesium sulfate and filtered. The residue was purified by column chromatography on silica gel (0–5% EA:Hex) to afford the product. Orange solid. Yield: 8.5%, mp: 203–205 °C, HPLC purity: 8 min, 97.00%, ^1^H NMR (400 MHz, DMSO-*d*_6_) *δ* 10.77 (s, 1H, indole-NH), 9.37 (s, 1H, amide-NH), 8.42 (s, 1H, Ph-H6′), 7.59–7.57 (d, *J* = 7.6 Hz, 1H, indolyl-H4), 7.41–7.38 (dd, *J* = 8.4, 2.3 Hz, 1H, Ph-H4′), 7.34-7.32 (d, *J* = 7.8 Hz, 1H, indolyl-H7), 7.14 (s, 1H, indolyl-H2), 7.13 (s, 2H, NH_2_), 7.08–7.05 (t, 1H, indolyl-H6), 7.00–6.94 (dd, *J* = 14.5, 8.0 Hz, 2H, Ph-H3′ and indolyl-H5), 3.04–2.98 (t, 2H, CH_2_), 2.87-2.74 (t, 2H, CH_2_). ^13^C NMR (100 MHz, DMSO-*d*_6_) *δ* 174.25 (amide-C=O), 153.10 (Ph-C2′), 138.83 (Ph-C5′), 137.01 (indole-C7a), 129.63 (Ph-C1′), 128.92 (indole-C3a), 124.94 (Ph-C4′), 124.83 (indole-C2), 123.51 (indole-C6), 122.33 (Ph-C3′), 121.01 (indole-C5), 120.76 (indole-C4), 117.50 (Ph-C6′), 116.22 (indole-C3), 113.91 (indole-C7), 39.31 (CH_2_-C=O), 23.46 (indole-CH_2_). HRMS (ESI) *m/z* calculated for C_17_H_17_N_3_O_4_S [M + H]^+^: 360.1018. Found: 360.1010.

**3-(1*H*-indol-3-yl)-*N*-(4-sulfamoylphenethyl)propanamide (2c)**. Following the synthetic procedure and work-up conditions of compound **2a**, 4-(2-aminoethyl)benzenesulfonamide was reacted with the starting material **1a**. The obtained residue was purified by column chromatography on silica gel (0–15% EA:Hex). White solid. Yield: 25%, mp: 236–237 °C, HPLC purity: 13 min, 99.00%, ^1^H NMR (400 MHz, DMSO-*d*_6_) *δ* 10.75 (s, 1H, indole-NH), 7.95 (t, 1H, amide-NH), 7.72–7.70 (d, *J* = 8.00 Hz, 2H, Ph-H3′ and H5′), 7.53–7.51 (d, *J* = 8.20 Hz, 1H, indolyl-H4)), 7.33 (s, 2H, NH_2_), 7.31–7.29 (d, *J* = 8.20 Hz, 3H, indoxyl-H7, and Ph-H2′ and H6′), 7.08 (s, 1H, indolyl-H2), 7.06–7.04 (d, *J* = 8.20 Hz, 1H, indolyl-H6)), 6.99–7.95 (t, *J* = 7.40 Hz, 1H, indolyl-H5), 3.29–3.26 (q, *J* = 6.50 Hz, 2H, CH_2_-NH), 2.90–2.88 (t, *J* = 7.4 Hz, 2H, CH_2_), 2.76–2.74 (t, *J* = 7.00 Hz, 2H, CH_2_), 2.43–2.39 (t, *J* = 7.60 Hz, 2H, CH_2_).^13^C NMR (100 MHz, DMSO-*d*_6_) *δ* 172.30 (amide-C=O), 144.40 (Ph-C1′), 142.40 (Ph-C4′), 136.64 (indole-C7a), 129.52 (Ph-C3′ and C5′), 127.44 (indole-C3a), 126.06 (Ph-C2′ and C6′), 122.53 (indole-C2), 121.30 (indole-C6), 118.78 (indole-C5), 118.54 (indole-C4), 114.26 (indole-C3), 111.71 (indole-C7), 36.75 (CH_2_-NH), 35.28 (CH_2_-C=O), 31.13 (CH_2_-Ph), 21.42 (indole-CH_2_). HRMS (ESI) *m/z* calculated for C_19_H_21_N_3_O_3_S [M + H]^+^: 372.1382. Found: 372.1371.

**3-(1*H*-indol-3-yl)-*N*-(4-sulfamoylphenyl)propanamide (2d)**. Following the synthetic procedure and work-up conditions of compound **2b**, 4-aminobenzenesulfonamide was reacted with the starting material **1a**. The obtained residue was purified by column chromatography on silica gel (0–25% EA:Hex). White solid. Yield: 30%, mp: 198–199 °C, HPLC purity: 9 min, 99.98%, ^1^H NMR (400 MHz, DMSO-*d*_6_) *δ* 10.78 (s, 1H, indole-NH), 10.27 (s, 1H, amide-NH), 7.75 (s, 4H, Ph-H2′, 3′, 5′, and 6′), 7.58–7.56 (d, *J* = 8.00 Hz, 1H, indolyl-H4), 7.34–7.32 (d, *J* = 8.00 Hz, 1H, indolyl-H7), 7.24 (s, 2H, NH_2_), 7.13 (s, 1H, indolyl-H2), 7.08–7.05 (t, *J* = 8.00 Hz, 1H, indolyl-H6), 7.00–6.96 (t, *J* = 8.00 Hz, 1H, indolyl-H5), 3.05–3.01 (t, *J* = 8.00 Hz, 2H, CH_2_), 2.71 (t, *J* = 8.06 Hz, 2H, CH_2_). ^13^C NMR (100 MHz, DMSO-*d*_6_) *δ* 171.98 (amide-C=O), 142.60 (Ph-C1′), 138.48 (indole-C7a), 136.65 (Ph-C4′), 127.40 (indole-C3a), 127.08 (Ph-C3′ and C5′), 122.63 (indole-C2), 121.37 (indole-C6), 118.93 (Ph-C2′ and C6′), 118.76 (indole-C5), 118.61 (indole-C4), 113.94 (indole-C7), 111.77 (indole-C3), 37.78 (CH_2_-C=O), 20.94 (indole-CH_2_). HRMS (ESI) *m/z* calculated for C_17_H_17_N_3_O_3_S [M + H]^+^: 344.1069. Found: 344.1065.

***N*-(3-sulfamoylphenyl)-1*H*-indole-5-carboxamide (2e)**. Following the synthetic procedure and work-up conditions of compound **2a**, 3-aminobenzenesulfonamide was reacted with the starting material **1b**. The obtained residue was purified by column chromatography on silica gel (0–20% EA:Hex) to obtain the product. White solid. Yield: 60%, mp: 182–182 °C, HPLC purity: 15 min, 96.78%, ^1^H NMR (400 MHz, DMSO-*d*_6_) *δ* 11.83 (s, 1H, indole-NH), 8.66 (s, 1H, indolyl-H4), 8.20–8.18 (d, *J* = 8.70 Hz, 1H, indolyl-H6), 7.98-7.96 (d, *J* = 8.60 Hz, 1H, Ph-H6′), 7.00-7.88 (d, *J* = 8.30 Hz, 1H, indolyl-H7), 7.69–7.67 (d, *J* = 8.60 Hz, 2H, Ph-H4′ and H5′), 7.62 (s, 1H, Ph-H2′), 7.57–7.53 (t, *J* = 7.60 Hz, 1H, indolyl-H2), 6.75 (s, 1H, indolyl-H3). ^13^C NMR (100 MHz, DMSO-*d*_6_) *δ* 164.22 (amide-C=O), 143.26 (Ph-C3′), 140.24 (Ph-C1′), 129.65 (Ph-C5′), 128.99 (indole-C7a), 128.81 (indole-C5), 128.11 (Ph-C6′), 125.72 (indole-C3a), 125.45 (indole-C2), 123.26 (Ph-C4′), 120.31 (Ph-C2′), 114.36 (indole-C4), 112.86 (indole-C7), 109.76 (indole-C6), 103.73 (indole-C3). HRMS (ESI) *m/z* calculated for C_15_H_13_N_3_O_3_S [M + H]^+^: 316.0756. Found: 279.0872, 230.0630, 144.0434, 120.0550, and 111.0198 (see Supplementary Materials).

***N*-(4-sulfamoylphenyl)-1*H*-indole-5-carboxamide (2f)**. Following the synthetic procedure and work-up conditions of compound **2a**, 4-aminobenzenesulfonamide was reacted with the starting material **1b**. The obtained residue was diluted with EA and the organic layer was washed with water and brine. The obtained residue was purified by column chromatography on silica gel (0–20% EA:Hex). White solid. Yield: 17%, mp: 257–259 °C, HPLC purity: 15 min, 100%, ^1^H NMR (400 MHz, DMSO-*d*_6_) *δ* 11.82 (s, 1H, indole-NH), 8.66 (s, 1H, indolyl-H4), 8.20–8.18 (d, *J* = 8.30 Hz, 1H, indolyl-H6), 7.98–7.95 (d, *J* = 8.7 Hz, 1H, indolyl-H7), 7.90–7.88 (d, *J* = 8.6 Hz, 1H, indolyl-H2), 7.69–7.55 (m, 4H, Ph-H2′, H3′, H5′, and H6′), 6.75 (s, 1H, indolyl-H3). ^13^C NMR (100 MHz, DMSO-*d*_6_) *δ* 164.22 (amide-C=O), 143.26 (Ph-C1′), 140.24 (Ph-C4′), 129.65 (Ph-C3′ and 5′), 128.81 (indole-C7a), 128.11 (indole-C5), 125.45 (indole-C3a), 123.26 (indole-C2), 120.31 (Ph-C2′ and 6′), 114.36 (indole-C4), 112.86 (indole-C7), 109.76 (indole-C6), 103.73 (indole-C3). HRMS (ESI) *m/z* calculated for C_15_H_13_N_3_O_3_S [M + H]^+^: 316.0756. Found: 279.0888, 144.0443, 120.0555, and 111.0207 (see Supplementary Materials).

***N*-(2-hydroxy-5-sulfamoylphenyl)-1*H*-indole-5-carboxamide (2g)**. Following the synthetic procedure in dimethylformamide/dichloromethane (DMF/DCM = 5/1) and work-up conditions of compound **2a**, 3-amino-4-hydroxybenzenesulfonamide was reacted with the starting material **1b**. The obtained residue was purified by column chromatography on silica gel (0–10% EA:Hex). White solid. Yield: 0.63%, mp: 189–190 °C, HPLC purity: 15 min, 99.62%, ^1^H NMR (400 MHz, DMSO-*d*_6_) *δ* 11.82 (s, 1H, indole-NH), 8.66 (s, 1H, indolyl-H4), 8.20–8.18 (d, *J* = 8.60 Hz, 1H, indolyl-H6), 7.98–7.95 (m, 1H, Ph-H4′), 7.90–7.88 (d, *J* = 8.30 Hz, 1H, indolyl-H7), 7.69–7.66 (m, 2H, Ph-H2′ and H6′), 7.63–7.61 (m, 1H, indolyl-H2), 6.75 (s, 1H, indolyl-H3). ^13^C NMR (100 MHz, DMSO-*d*_6_) *δ* 168.84 (amide-C=O), 138.74 (indole-C7a), 128.22 (Ph-C5′), 127.76 (indole-C3a), 127.59 (Ph-C1′), 127.34 (indole-C5), 124.93 (indole-C2), 123.22 (Ph-C4′), 122.62 (Ph-C3′), 121.80 (indole-C4), 119.57 (Ph-C6′), 111.52 (indole-C7), 110.02 (indole-C6), 102.90 (indole-C3). HRMS (ESI) *m/z* calculated for C_15_H_13_N_3_O_4_S [M + H]^+^: 332.0705. Found: 332.0701.

***N*-(4-sulfamoylphenethyl)-1*H*-indole-5-carboxamide (2h)**. Following the synthetic procedure and work-up conditions of compound **2b**, 4-(2-aminoethyl)benzenesulfonamide was reacted with the starting material **1b**. The obtained residue was purified by column chromatography on silica gel (0–6% DCM:MeOH). Orange solid. Yield: 35%, mp: 209–211 °C, HPLC purity: 6 min, 97.68%, ^1^H NMR (400 MHz, DMSO-*d*_6_) *δ* 11.31 (s, 1H, indole-NH), 8.43–8.41 (t, 1H, amide-NH), 8.08 (s, 1H, indolyl-H4), 7.75–7.73 (d, *J* = 8.00 Hz, 2H, Ph-H3′ and H5′), 7.61–7.59 (d, *J* = 8.00 Hz, 1H, indolyl-H6), 7.45–7.39 (m, 4H, indolyl-H2 and H7 & Ph-H2′ and H6′), 7.28 (s, 2H, NH_2_), 6.52 (s, 1H, indolyl-H3), 3.55–3.50 (m, 2H, CH_2_), 2.96–2.92 (t, *J* = 8.00 Hz, 2H, CH_2_-Ph). ^13^C NMR (100 MHz, DMSO-*d*_6_) *δ* 167.71 (amide-C=O), 144.42 (Ph-C1′), 142.39 (Ph-C4′), 137.75 (indole-C7a), 129.56 (Ph-C3′ and C5′), 127.38 (indole-C3a), 127.05 (indole-C5), 126.10 (Ph-C2′ and C6′), 125.95 (indole-C2), 120.86 (indole-C4), 120.17 (indole-C7), 111.26 (indole-C6), 102.47 (indole-C3), 35.40 (CH_2_-NH), 31.12 (CH_2_-Ph). HRMS (ESI) *m/z* calculated for C_17_H_17_N_3_O_3_S [M + H]^+^ 344.1069. Found: 344.1060.

***N*-(2-sulfamoylphenyl)-1*H*-indole-5-carboxamide (2i)**. Following the synthetic procedure and work-up conditions of compound **2a**, 2-aminobenzenesulfonamide was reacted with the starting material **1b**. The obtained residue was purified by column chromatography on silica gel (0–15% EA:Hex). White solid. Yield: 23%, mp: 192–193 °C, HPLC purity: 15 min, 98.02%, ^1^H NMR (400 MHz, DMSO-*d*_6_) δ 12.40 (s, 1H, amide-NH), 11.43 (s, 1H, indole-NH), 8.23 (s, 1H, indolyl-H4), 8.00–7.98 (d, *J* = 8.1 Hz, 1H, indolyl-H6), 7.74–7.70 (t, *J* = 8.0 Hz, 2H, Ph-H4′ and H5′), 7.57–7.53 (t, 1H, indolyl-H2), 7.45–7.40 (m, 3H, indolyl-H7, and Ph-H3′ and H6′), 6.57 (s, 1H, indolyl-H3). ^13^C NMR (100 MHz, DMSO-*d*_6_) *δ* 164.21 (amide-C=O), 143.26 (Ph-C2′), 140.24 (Ph-C1′), 129.63 (Ph-C5′), 128.99 (indole-C7a), 128.80 (indole-C5), 128.11 (Ph-C6′), 125.71 (indole-C3a), 125.44 (indole-C2), 123.25 (Ph-C4′), 120.31 (Ph-C2′), 114.36 (indole-C4), 112.85 (indole-C7), 109.75 (indole-C6), 103.72 (indole-C3). 

***N*-(3-sulfamoylphenyl)-1*H*-indole-6-carboxamide (2j)**. Following the synthetic procedure and work-up conditions of compound **2a**, 3-aminobenzenesulfonamide was reacted with the starting material **1c**. The obtained residue was recrystallized twice using methanol and DCM. Orange solid. Yield: 32%, mp: 201–211 °C, HPLC purity: 15 min, 99.67%, ^1^H NMR (400 MHz, DMSO-*d*_6_) *δ* 11.83 (s, 1H, indole-NH), 8.43 (s, 1H, indolyl-H7), 8.20–8.18 (d, *J* = 8.5 Hz, 1H, indolyl-H4), 7.92–7.80 (m, 4H, indolyl-H5, and Ph-H2′, H4′, H6′), 7.70–7.66 (m, 1H, Ph-H5′), 7.58–7.54 (m, 1H, indolyl-H2), 6.69 (s, 1H, indolyl-H3). ^13^C NMR (100 MHz, DMSO-*d*_6_) *δ* 164.24 (amide-C=O), 143.26 (Ph-C3′), 135.48 (Ph-C1′), 133.75 (indole-C7a), 131.98 (Ph-C5′), 129.65 (Ph-C6′), 129.00 (indole-C3a), 125.73 (indole-C6), 121.25 (indole-C2), 120.74 (Ph-C4′), 120.31 (Ph-C2′), 115.76 (indole-C4), 115.73 (indole-C5), 109.79 (indole-C7), 102.72 (indole-C3). 

***N*-(4-sulfamoylphenyl)-1*H*-indole-6-carboxamide (2k)**. Following the synthetic procedure and work-up conditions of compound **2a**, 4-aminobenzenesulfonamide was reacted with the starting material **1c**. The obtained residue was purified by column chromatography on silica gel (0–20% EA:Hex). White solid. Yield: 13%, mp: 221–222 °C, HPLC purity: 15 min, 99.90%, ^1^H NMR (400 MHz, DMSO-*d*_6_) *δ* 11.83 (s, 1H, indole-NH), 8.42 (s, 1H, indolyl-H7), 8.20–8.18 (d, *J* = 8.5 Hz, 1H, indolyl-H4), 7.92–7.60 (m, 4H, Ph-H2′, H3′, H5′ and H6′), 7.70–7.66 (t, 1H, indolyl-H5), 7.58–7.53 (t, 1H, indolyl-H2), 6.69 (s, 1H, indolyl-H3). ^13^C NMR (100 MHz, DMSO-*d*_6_) *δ* 168.80 (amide-C=O), 135.58 (Ph-C1′), 131.47 (Ph-C4′), 129.32 (indole-C4), 128.23 (indole-C7a), 127.80 (indole-C3a), 124.93 (indole-C6), 123.52 (indole-C2), 120.20 (Ph-C2′ and C6′), 120.01 (Ph-C3′ and C5′), 113.94 (indole-C5), 109.99 (indole-C7), 101.87 (indole-C3). 

***N*-(2-hydroxy-5-sulfamoylphenyl)-1*H*-indole-6-carboxamide (2l)**. Following the synthetic procedure and work-up conditions of compound **2a**, 3-amino-4-hydroxybenzenesulfonamide was reacted with the starting material **1c**. The obtained residue was purified by column chromatography on silica gel (0–15% EA:Hex). Orange solid. Yield: 23%, mp: 213–215 °C, HPLC purity: 15 min, 98.03%, ^1^H NMR (400 MHz, DMSO-*d*_6_) δ 11.45 (s, 1H, indole-NH), 8.05 (s, 1H, indolyl-H7), 8.00–7.98 (d, *J* = 8.4 Hz, 1H, indolyl-H4), 7.73–7.71 (d, *J* = 8.4 Hz, 1H, Ph-H3′), 7.60–7.53 (m, 4H, indolyl-H5, OH, and Ph-H4′ and H6′), 7.44–7.40 (t, 1H, indolyl-H2), 6.52 (s, 1H, indolyl-H3). ^13^C NMR (100 MHz, DMSO-*d*_6_) *δ* 168.80 (amide-C=O), 135.57 (indole-C7a), 131.46 (Ph-C5′), 129.33 (indole-C3a), 128.22 (Ph-C1′), 127.74 (Ph-C4′), 124.93 (indole-C2), 123.51 (indole-C6), 120.20 (indole-C4), 120.01 (Ph-C3′), 119.53 (Ph-C6′), 113.94 (indole-C5), 110.04 (indole-C7), 101.87 (indole-C3). HRMS (ESI) *m/z* calculated for C_15_H_13_N_3_O_4_S [M + H]^+^, 332.0705. Found: 332.0704.

***N*-(4-sulfamoylphenethyl)-1*H*-indole-6-carboxamide (2m)**. Following the synthetic procedure with solvent (DMF/DCM = 5/1) and work-up conditions of compound **2a**, 4-(2-aminoethyl)benzenesulfonamide was reacted with the starting material **1c**. The obtained residue was purified by column chromatography on silica gel (0–10% EA:Hex). White solid. Yield: 28%, mp: 250–252 °C, HPLC purity: 1 min, 96.73%, ^1^H NMR (400 MHz, DMSO-*d*_6_) *δ* 11.37 (s, 1H, indole-NH), 8.49–8.46 (t, *J* = 8.00 Hz, 1H, amide-NH), 7.92 (s, 1H, indolyl-H7), 7.76–7.74 (d, *J* = 8.00 Hz, 2H, Ph-H3′ and H5′), 7.57–7.55 (d, *J* = 8.00 Hz, 1H, indolyl-H5), 7.50–7.48 (m, 2H, indolyl-H2 and H4), 7.45–7.43 (d, *J* = 8.00 Hz, 2H, Ph-H2′ and H6′), 7.28 (s, 2H, NH_2_), 6.47(s, 1H, indolyl-H3), 3.55–3.50 (q, 2H, CH_2_-NH), 2.95–2.93 (t, *J* = 8.00 Hz, 2H, CH_2_-Ph). ^13^C NMR (100 MHz, DMSO-*d*_6_) *δ* 167.63 (amide-C=O), 144.40 (Ph-C1′), 142.43 (Ph-C4′), 135.65 (indole-C7a), 130.17 (indole-C3a), 129.55 (Ph-C3′ and C5′), 128.31 (indole-C2), 127.82 (indole-C6), 126.11 (Ph-C2′ and C6′), 119.78 (indole-C4), 118.31 (indole-C5), 111.52 (indole-C7), 101.61 (indole-C3), 40.92 (CH_2_-NH), 35.38 (CH_2_-Ph). 

***N*-(2-sulfamoylphenyl)-1*H*-indole-6-carboxamide (2n)**. Following the synthetic procedure and work-up conditions of compound **2a**, 2-aminobenzenesulfonamide was reacted with the starting material **1c**. The obtained residue was purified by column chromatography on silica gel (0–12% EA:Hex). White solid. Yield: 10%, mp: 222–223 °C, HPLC purity: 15 min, 98.70%, ^1^H NMR (400 MHz, DMSO-*d*_6_) *δ* 11.82 (s, 1H, indole-NH), 8.42 (s, 1H, indolyl-H7), 8.20–8.18 (d, *J* = 8.4 Hz, 1H, indolyl-H4), 7.91–7.89 (d, 2H, Ph-H3′ and H6′), 7.81–7.78 (t, 1H, Ph-H4′), 7.70–7.66 (t, *J* = 7.3 Hz, 1H, Ph-H5′), 7.57–7.54 (t, 1H, indolyl-H2), 6.69 (s, 1H, indolyl-H3). ^13^C NMR (100 MHz, DMSO-*d*_6_) *δ* 164.24 (amide-C=O), 143.26 (Ph-C2′), 135.48 (Ph-C1′), 133.74 (indole-C7a), 129.66 (Ph-C3′), 128.99 (indole-C3a), 125.74 (indole-C6), 121.25 (indole-C2), 120.74 (Ph-C5′), 120.32 (Ph-C4′), 120.20 (Ph-C6′), 115.75 (indole-C4), 115.72 (indole-C5), 109.80 (indole-C7), 102.72 (indole-C3). 

***N*-(1*H*-indol-5-yl)-4-sulfamoylbenzamide (2o)**. Following the synthetic procedure with solvent (DMF/DCM = 5/1) and work-up conditions of compound **2a**, 4-sulfamoylbenzoic acid was reacted with the starting material **1d**. The obtained residue was purified by column chromatography on silica gel (0–8% DCM:MeOH). Purple solid. Yield: 25%, mp: 261–262 °C, HPLC purity: 6 min, 97.38%, ^1^H NMR (400 MHz, DMSO-*d*_6_) *δ* 11.06 (s, 1H, indole-NH), 10.26 (s, 1H, amide-NH), 8.13–8.11 (d, *J* = 8.00 Hz, 2H, Ph-H2′ and H6′), 8.00 (s, 1H, indolyl-H4′), 7.96–7.94 (d, *J* = 8.00 Hz, 2H, Ph-H3′ and H5′), 7.52 (s, 2H, NH_2_), 7.41–7.33 (m, 3H, indolyl-H2, H6, and H7), 6.42 (s, 1H, indolyl-H3). ^13^C NMR (100 MHz, DMSO-*d*_6_) *δ* 164.44 (amide-C=O), 146.61 (Ph-C4′), 138.79 (Ph-C1′), 133.52 (indole-C7a), 131.07 (indole-C5), 128.64 (Ph-C2′ and C6′), 127.81 (indole-C2), 126.43 (indole-C3a), 126.03 (Ph-C3′ and C5′), 116.38 (indole-C6), 112.64 (indole-C7), 111.53 (indole-C4), 101.63 (indole-C3). HRMS (ESI) *m/z* calculated for C_15_H_13_N_3_O_3_S [M + H]^+^ 316.0756. Found: 316.0757.

### 3.2. Carbonic Anhydrase Inhibition

The inhibitor and enzyme solutions were first preincubated together for 15 min at room temperature to enable for the generation of the E–I complex. Photophysics stopped-flow instrument was used to measure the CA-catalyzed CO_2_ hydration activity. Phenol red (at a concentration of 0.2 mM) was added as an indicator after a period of 10–100 s in the CA-catalyzed CO_2_ hydration reaction at an absorbance maximum of 557 nm, with 20 mM Hepes (pH = 7.4) and 10 mM NaClO_4_ (to maintain constant ionic strength) [30]. The kinetic parameters and inhibition constants were calculated at CO_2_ concentrations ranging from 1.7 to 17 mM [31]. In the initial 5–10% of the reaction, at least six traces were examined to determine the initial velocity for each inhibitor. Similarly, the total measured rates were used to measure the uncatalyzed rates [32]. Distilled–deionized water was used in the preparation of the Stock inhibitor solutions (10 mM), as well as dilutions up to 0.01 nM. The inhibition constants were measured utilizing PRISM 3, whereas the Lineweaver–Burk plots were used to measure the kinetic parameters which comprised the mean of at least three separate measurements [33,34].

### 3.3. Molecular Docking

hCA I (PDB code: 6Y00, resolution 1.37 Å) [35], hCA II (PDB code: 4BF1, resolution 1.35 Å) and hCA IX (PDB code: 4ZWX, resolution 1.70 Å) [36] crystal structures were retrieved from the Protein Data Bank (www.pdb.org, accessed on 19 February 2022). The downloaded crystal structures were prepared employing the preparation wizard of the Schrodinger 2021 suite package under the default settings with the pH value set at a value of 7.4. All ligands were first sketched via ChemDraw Professional 17.0, then exported as a structure data file format and sent to the Ligprep module. Ligand preparation was achieved utilizing the Schrodinger Ligprep module to optimize the geometry of the studied ligands. Glide’s standard precision module was employed to dock the minimized ligands into the appropriate crystal structure binding site, yielding 10 poses for each docked ligand. 

## 4. Conclusions

A new hybrid series of indole-based benzenesulfonamides were successfully synthesized, characterized, and biologically evaluated against four isoforms of CA (I, II, IX, and XII). The inhibitory activities of the hybrid compounds **2a**–**o** and AAZ, as a standard inhibitor, were measured against the four isoforms of hCA by a stopped flow CO_2_ hydrase assay. Compared to AAZ, compounds **2a**, **2d** and **2o** showed higher inhibitory activities over hCA I. Among all, compound **2a** displayed the most potent activity and selectivity against hCA II. Compounds **2b**, **2c**, **2d**, **2f**, **2h** and **2o** also showed low nanomolar inhibitory activities against hCA II. The selectivity profiles of the synthesized target compounds, as well as their SAR, display the high potential of this series to be further investigated as a promising lead towards more potent and selective hCA II inhibitors. 

## Figures and Tables

**Table 1:** Starting materials, reagents, and chemical structures of compounds **2a**–**o**.

Cpd	Starting	Benzenesulfonamide Reagent	Chemical Structure
**2a**	**1a**	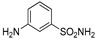	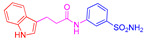
**2b**	**1a**	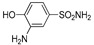	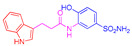
**2c**	**1a**	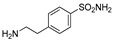	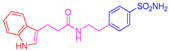
**2d**	**1a**	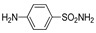	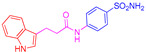
**2e**	**1b**	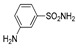	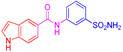
**2f**	**1b**	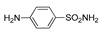	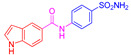
**2g**	**1b**	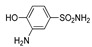	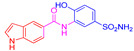
**2h**	**1b**	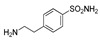	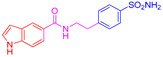
**2i**	**1b**	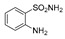	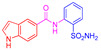
**2j**	**1c**	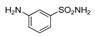	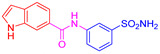
**2k**	**1c**	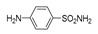	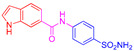
**2l**	**1c**	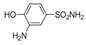	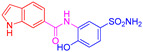
**2m**	**1c**	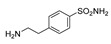	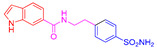
**2n**	**1c**	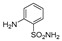	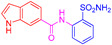
**2o**	**1d**	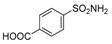	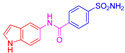

**Table 2:** Inhibition of hCA isoforms I, II, IX and XII by compounds **2a**–**o**; acetazolamide (AAZ) was used as a standard inhibitor.

Cpd	K_i_ (nM)			
	hCA I	hCA II	hCA IX	hCA XII
**2a**	79.8	5.9	205.8	54.2
**2b**	2796	7.3	286.5	85.1
**2c**	217.3	9.0	221.6	36.9
**2d**	97.6	7.1	165.5	42.2
**2e**	5575	45.5	766.3	90.9
**2f**	3105	16.0	165.1	257.0
**2g**	4772	679.9	279.6	85.3
**2h**	553.7	8.6	239.6	235.6
**2i**	7453	71.9	635.0	89.4
**2j**	5397	94.8	654.0	40.5
**2k**	4655	833.4	427.6	83.8
**2l**	4275	681.6	423.0	73.7
**2m**	252.9	38.8	277.9	85.1
**2n**	7511	670.1	487.3	36.9
**2o**	56.6	7.5	169.6	66.9
**AAZ**	250.0	12.1	25.7	5.7

## Data Availability

Data are contained within the article.

## Supplementary Materials

The following supporting information can be downloaded at: https://www.mdpi.com/article/10.3390/ijms23052540/s1. NMR, HPLC, and HRMS representative data for the newly synthesized compounds in this article.
