# Supplementary material for: Identification of Novel and Potent Indole-Based Benzenesulfonamides as Selective Human Carbonic Anhydrase II Inhibitors: Design, Synthesis, In Vitro, and In Silico Studies
Source: Int J Mol Sci. 2022 Feb 25;23(5):2540. doi: 10.3390/ijms23052540 (PMC8910009; doi:10.3390/ijms23052540)
Supplement: Supplementary file 1 [file ijms-23-02540-s001.zip › supplementary.v3.pdf]

Supplementary material

Identification of Novel and Potent Indole-based Benzenesulfonamides as Selective Human Carbonic Anhydrase II Inhibitors: Design, Synthesis, In Vitro, and In Silico Studies

Contents

1. ^{1}HNMR and ^{13}CNMR data of compounds **(2a-2o)**

2. HPLC purity

3. HRMS data

4. HRMS interpretation

1. ^{1}HNMR and ^{13}CNMR data of compounds 2a-2o

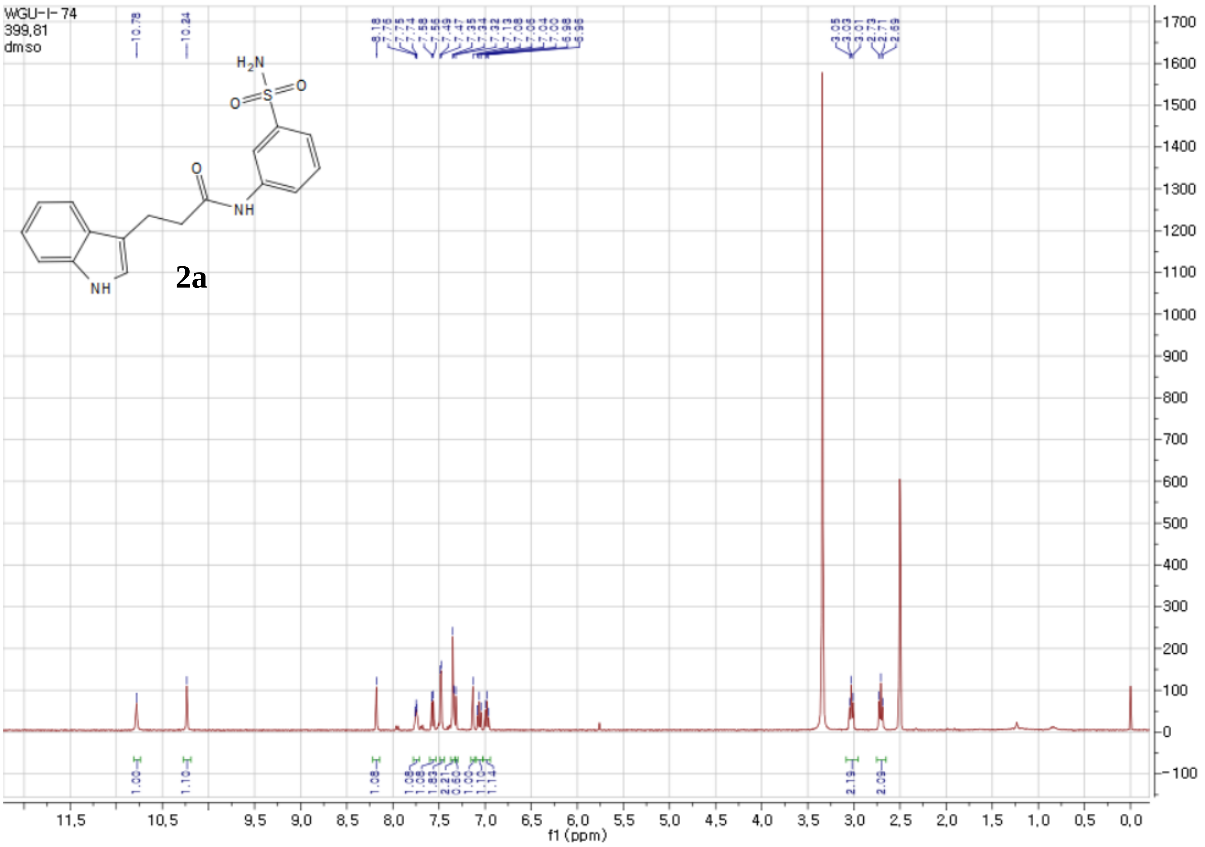

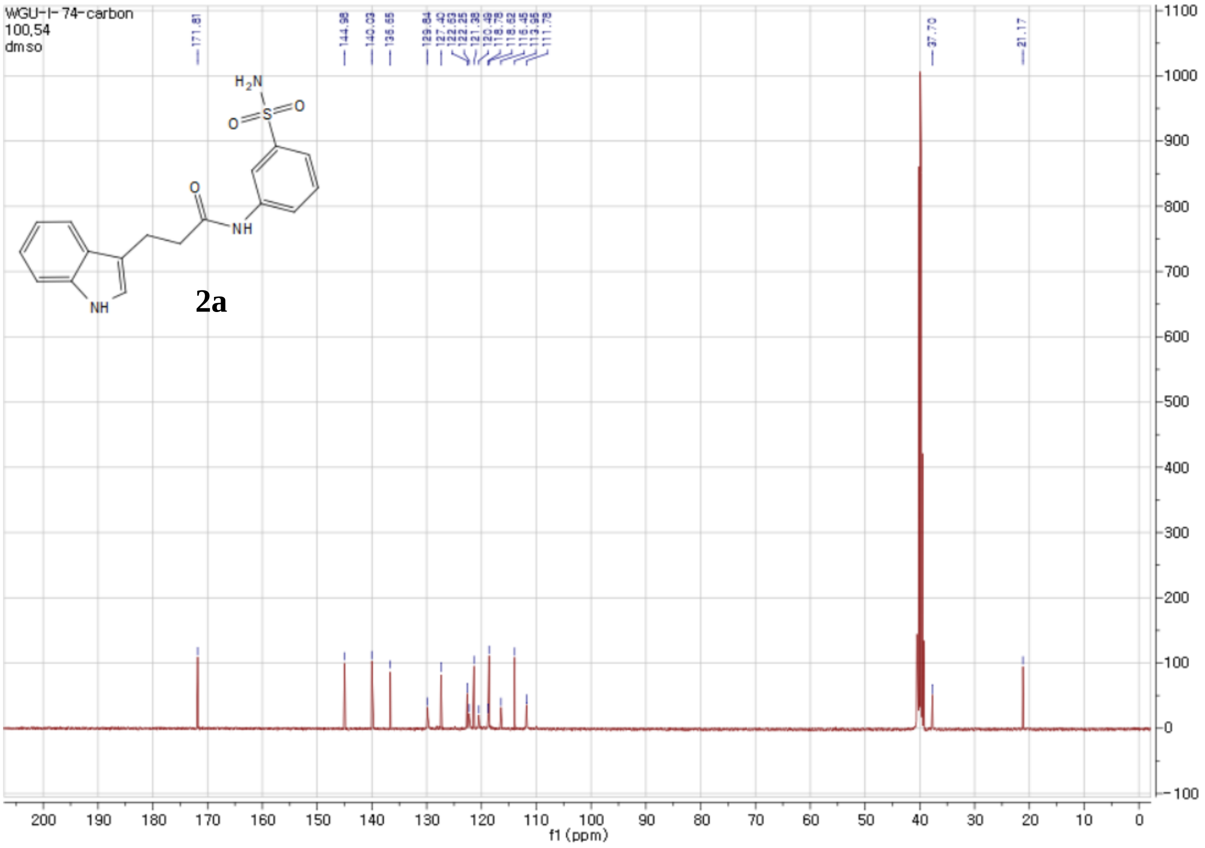

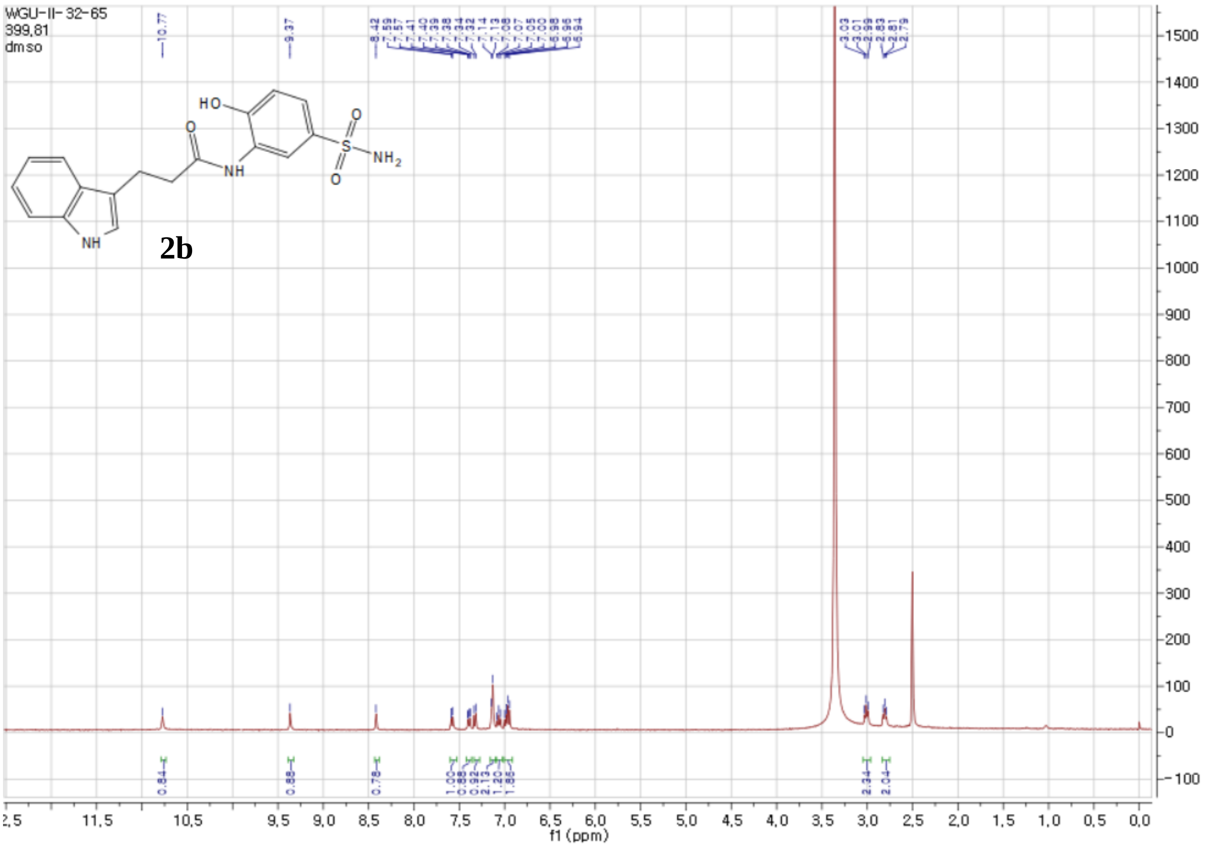
WGU-II-32-65
399,81
dmso
HO
O
NH
S
NH2
NH
f1 (ppm)

2b

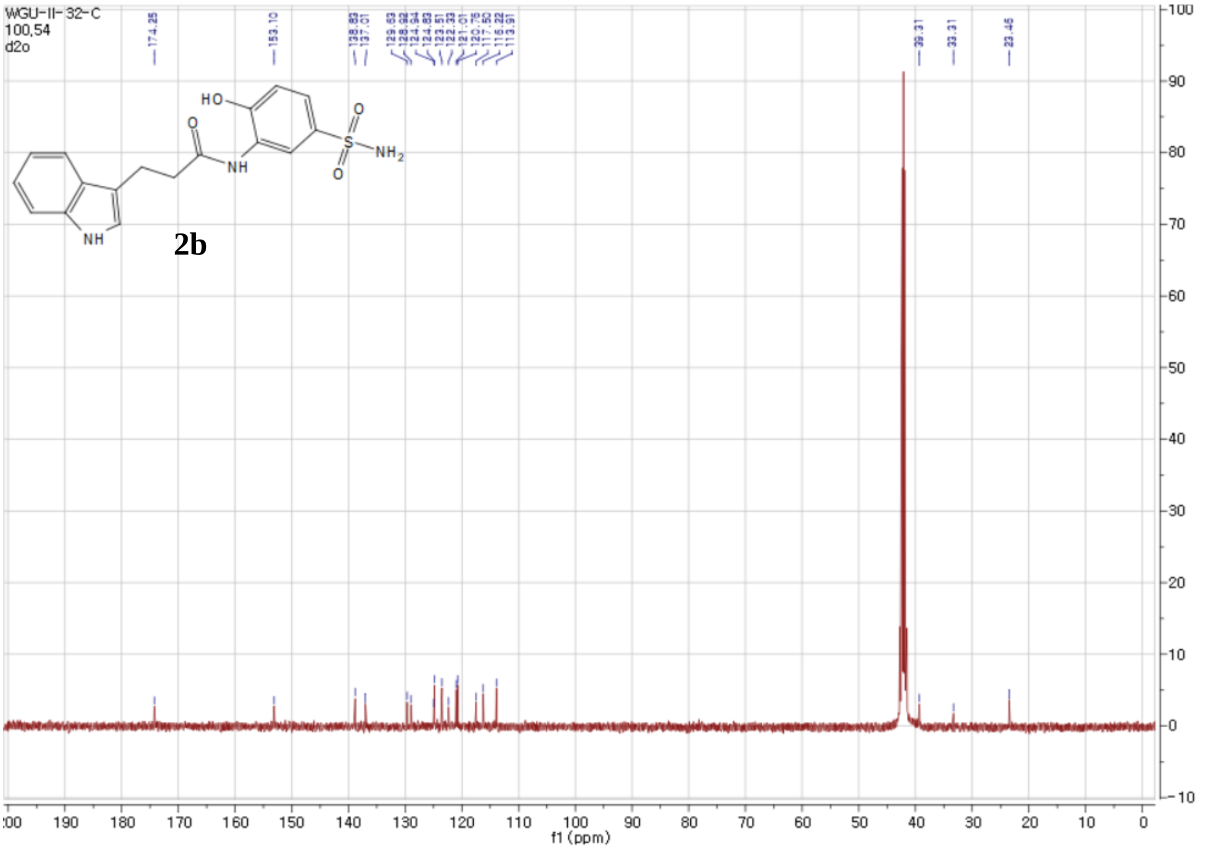
WGU-II-32-C
100,54
d2o
HO
O
NH
S
NH2
NH
f1 (ppm)

2b

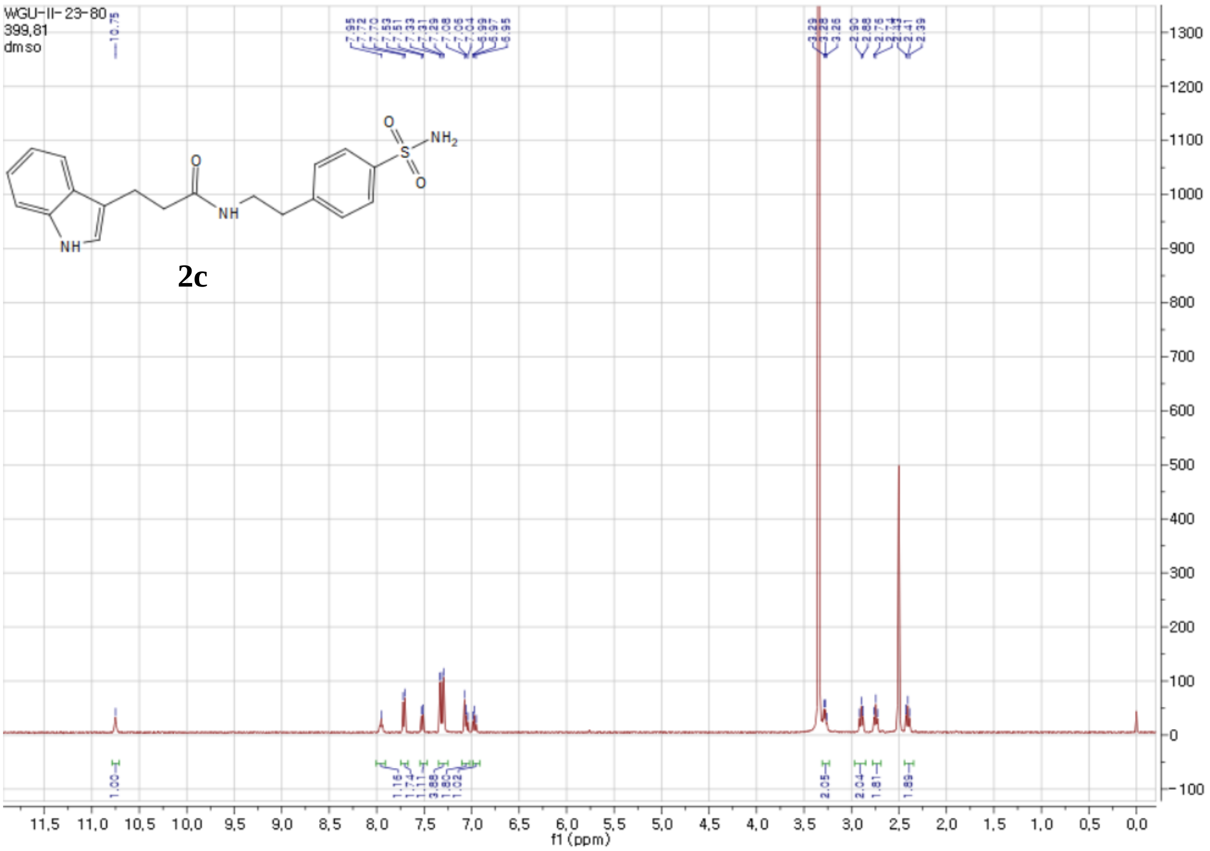
WGU-II-23-80
399,81
dmso
2c
f1 (ppm)

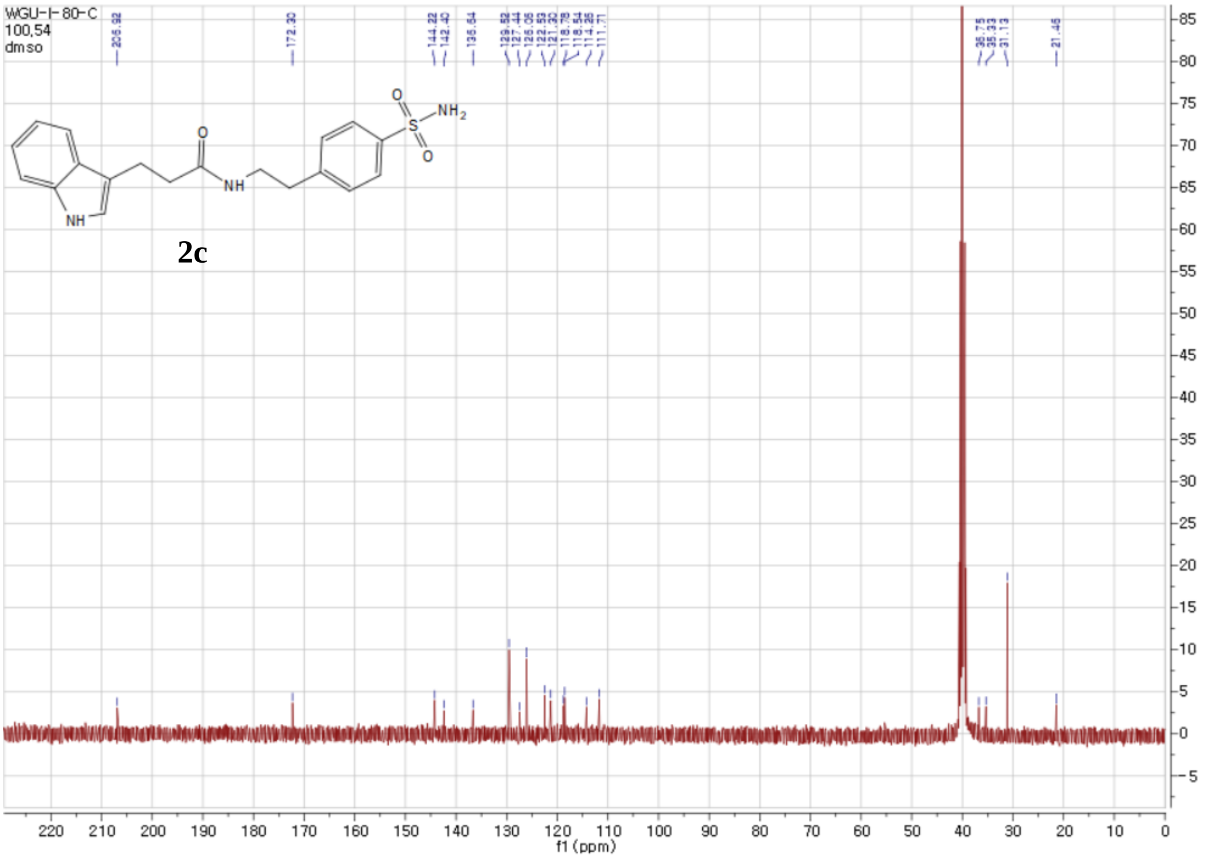
WGU-I-80-C
100,54
dmso
2c
f1 (ppm)

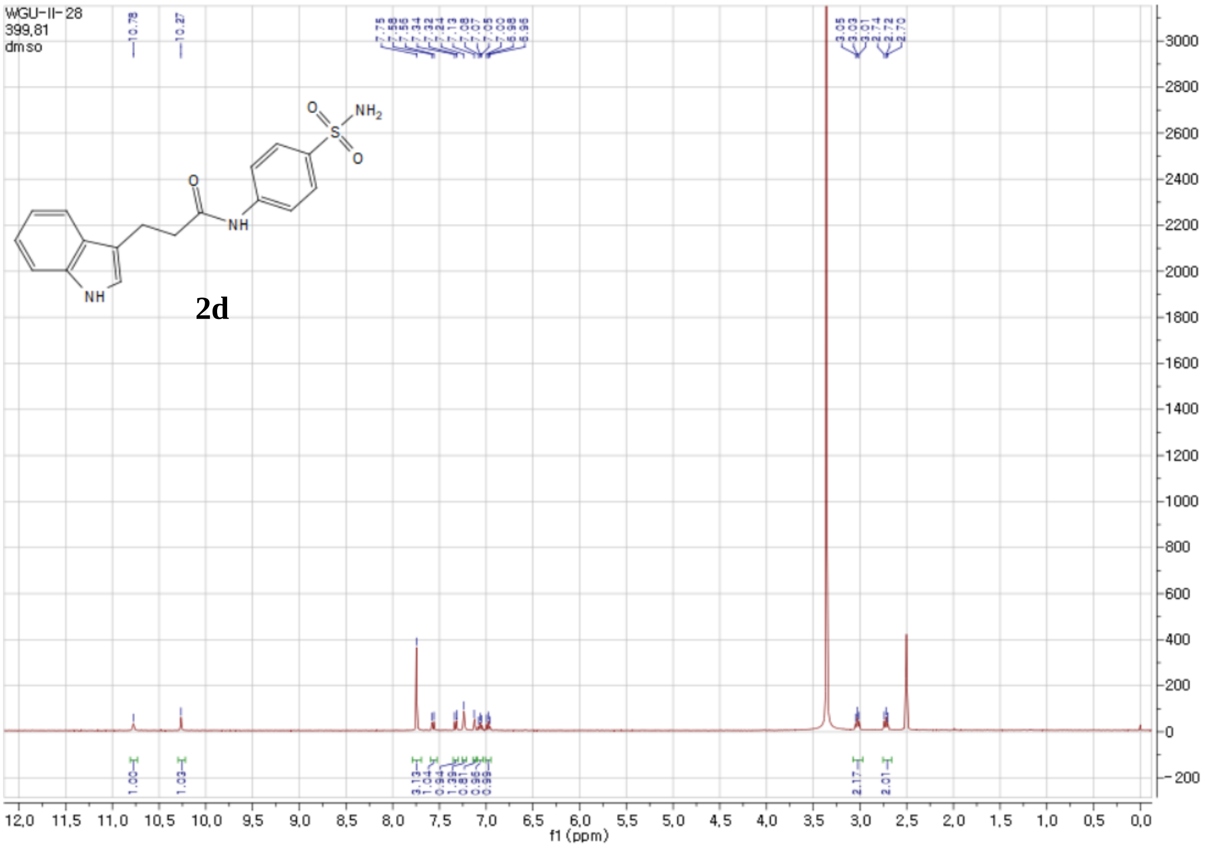
WGU-II-28
399,81
dmso
2d
f1 (ppm)

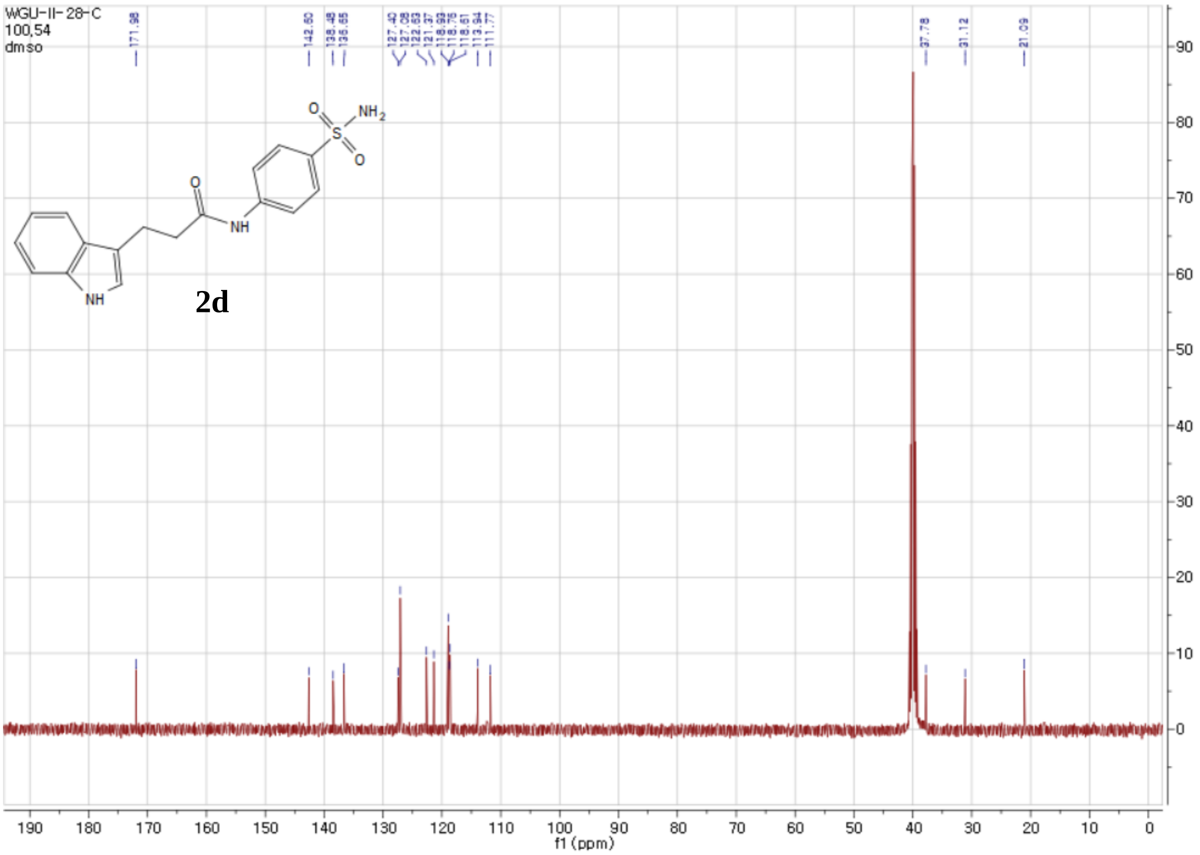
WGU-II-28-C
100,54
dmso
2d
f1 (ppm)

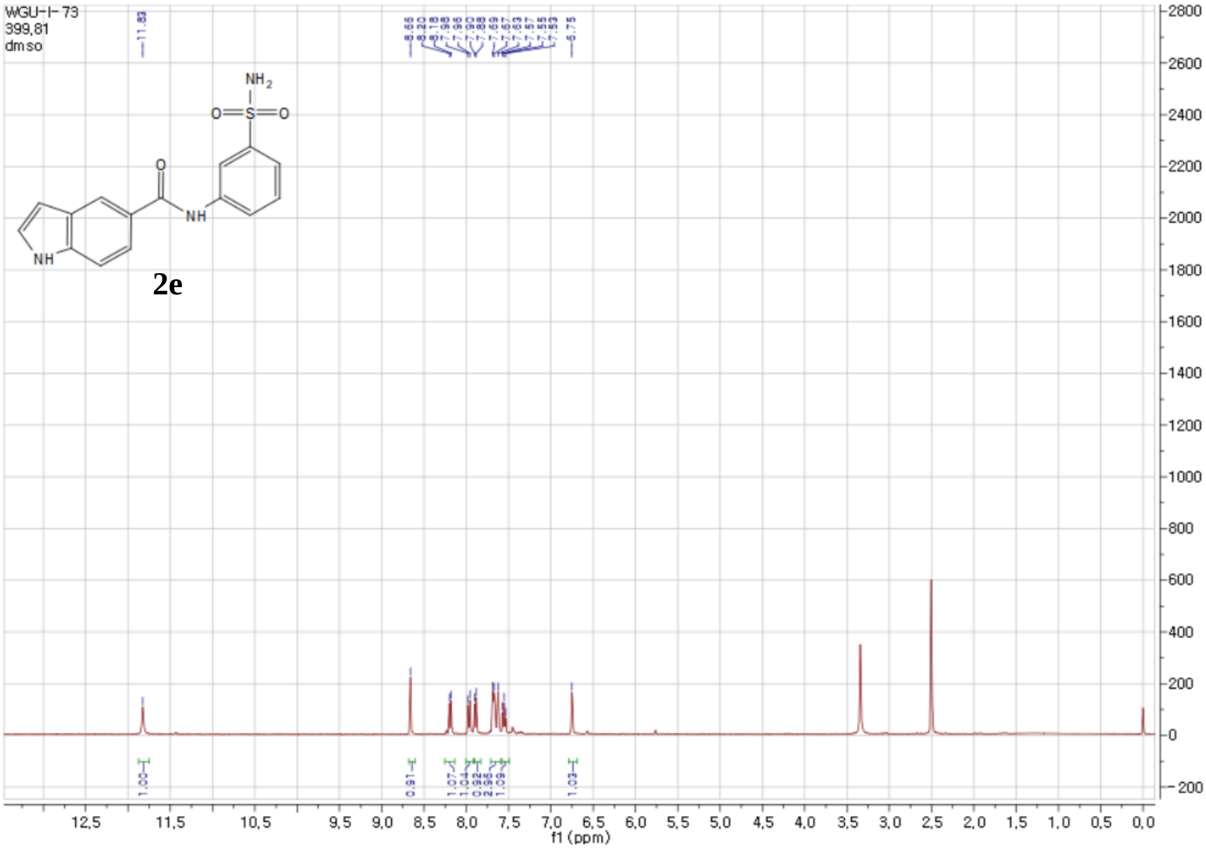

WGU-I-73
399,81
dmso
11.83
6.75
NH2
O=S=O
O
NH
NH
f1 (ppm)

2e

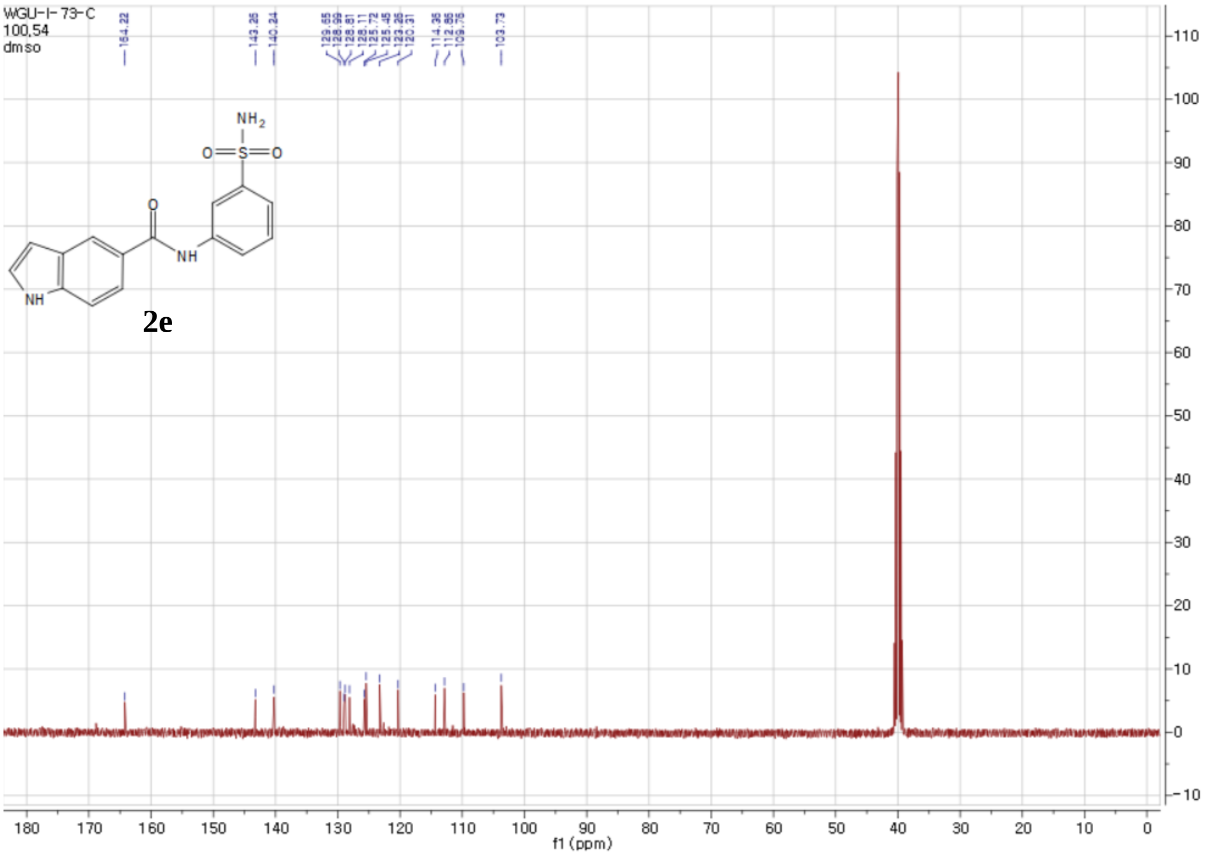

WGU-I-73-C
100,54
dmso
164.22
143.28
140.24
103.73
NH2
O=S=O
O
NH
NH
f1 (ppm)

2e

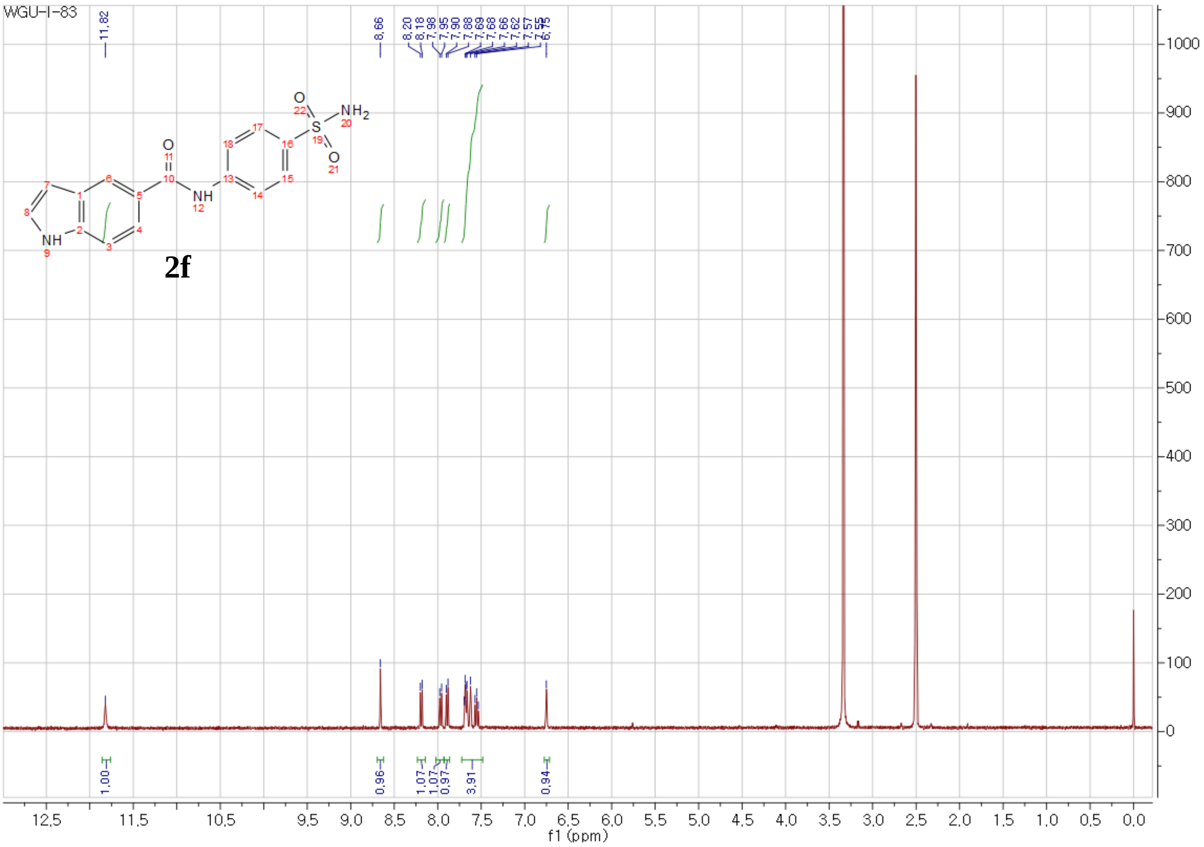
WGU-I-83
11.82
8.66
8.20
8.18
7.98
7.95
7.90
7.88
7.69
7.68
7.66
7.62
7.57
7.55
6.73
2f
1.00
0.96
1.07
1.07
0.97
3.91
0.94
f1 (ppm)

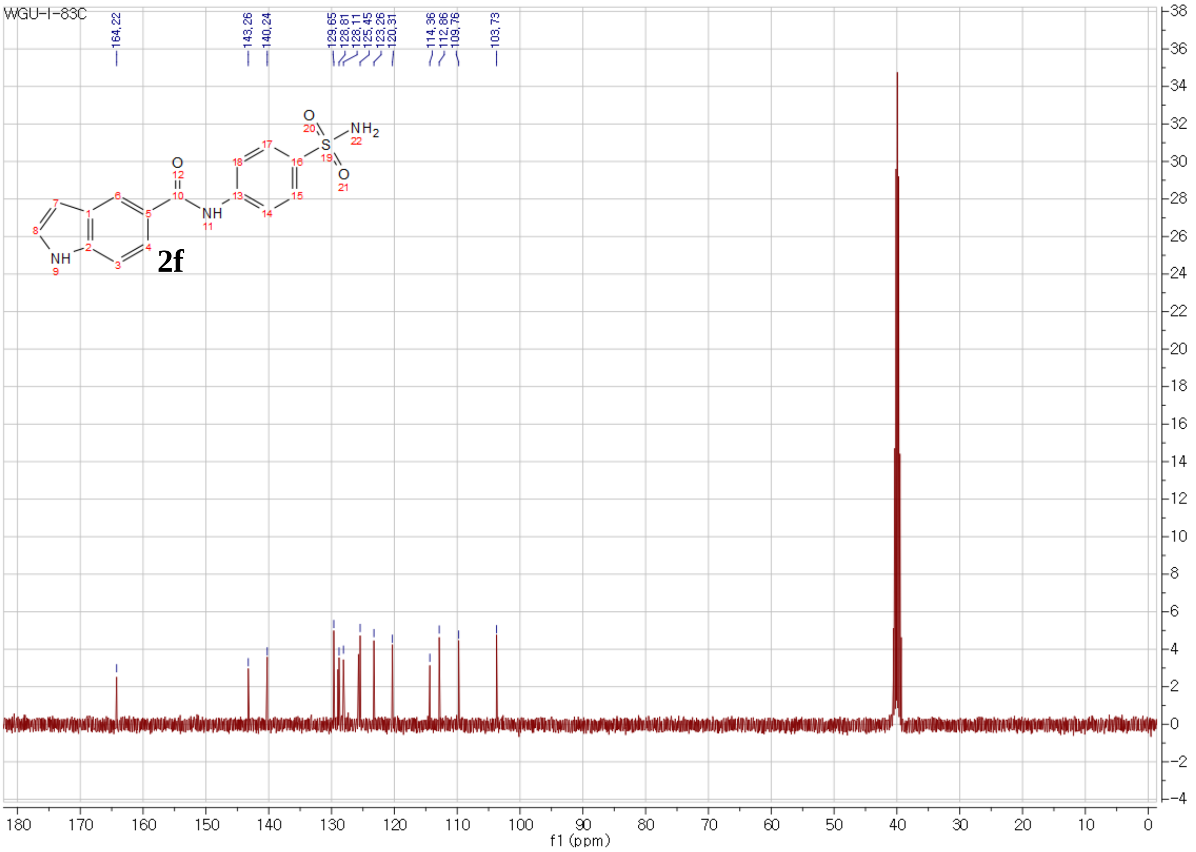
WGU-I-83C
164.22
143.26
140.24
129.65
128.61
128.11
125.45
123.26
120.31
114.36
112.86
109.76
103.73
2f
f1 (ppm)

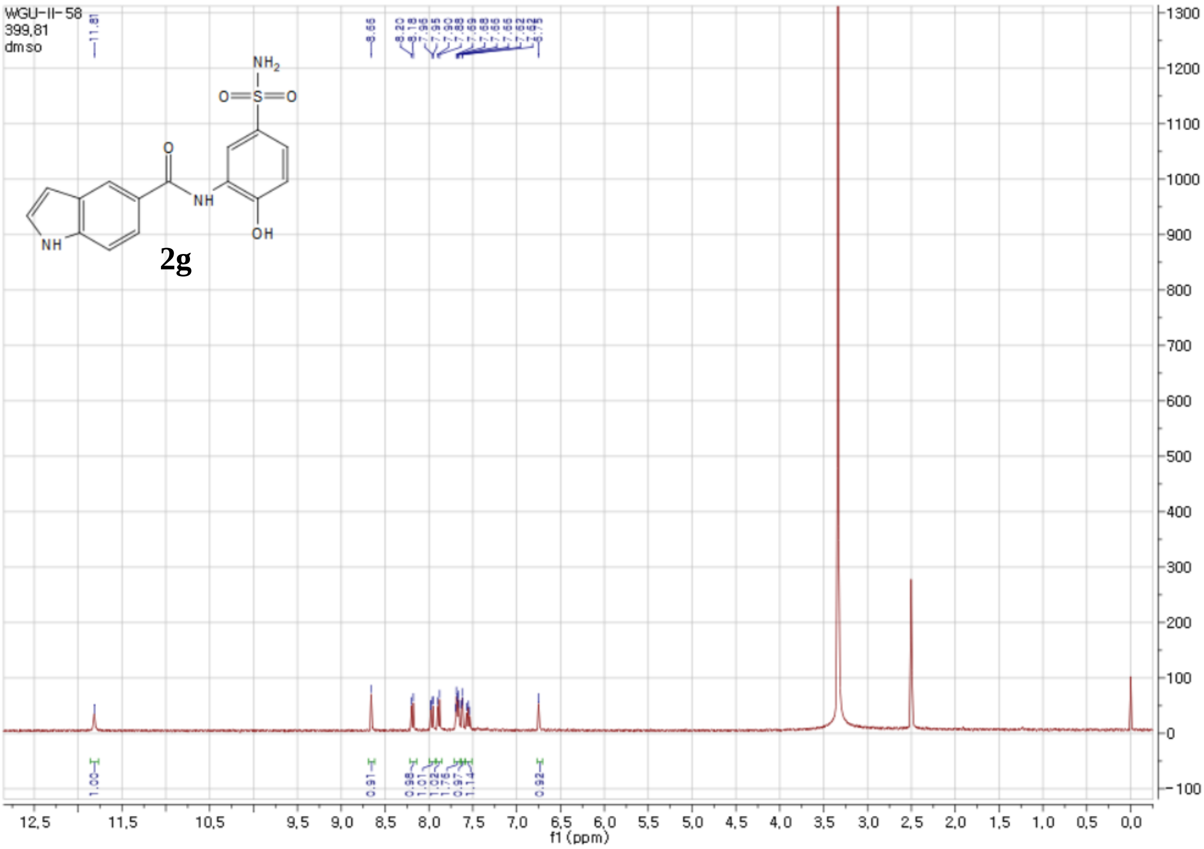
WGU-II-58
399,81
dmso
11.81
8.66
8.20
8.18
6.75
NH2
O=S=O
O
NH
OH
NH
2g
1.00
0.91
0.98
1.01
1.02
1.75
0.97
1.14
0.92
f1 (ppm)

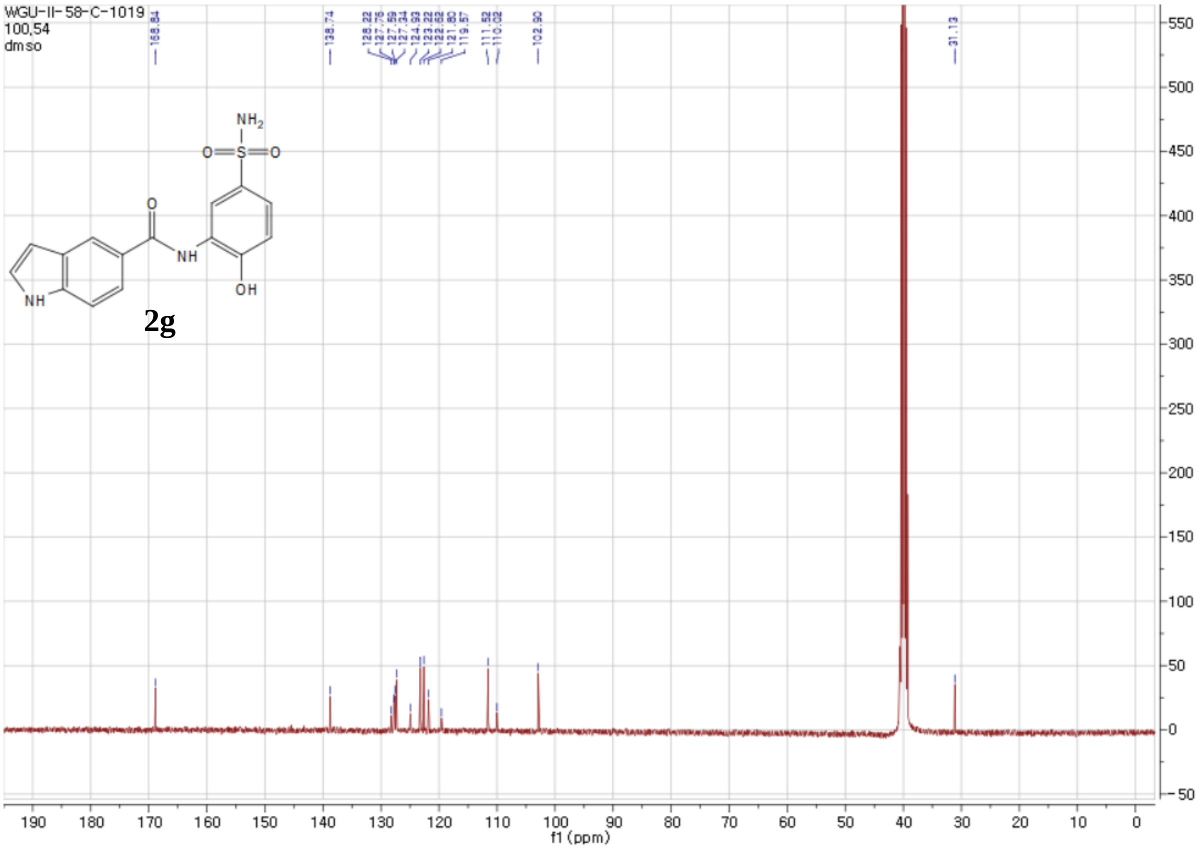
WGU-II-58-C-1019
100,54
dmso
168.84
138.74
128.22
127.75
127.59
127.34
124.93
123.22
122.62
121.80
119.57
111.52
110.02
102.90
31.13
NH2
O=S=O
O
NH
OH
NH
2g
f1 (ppm)

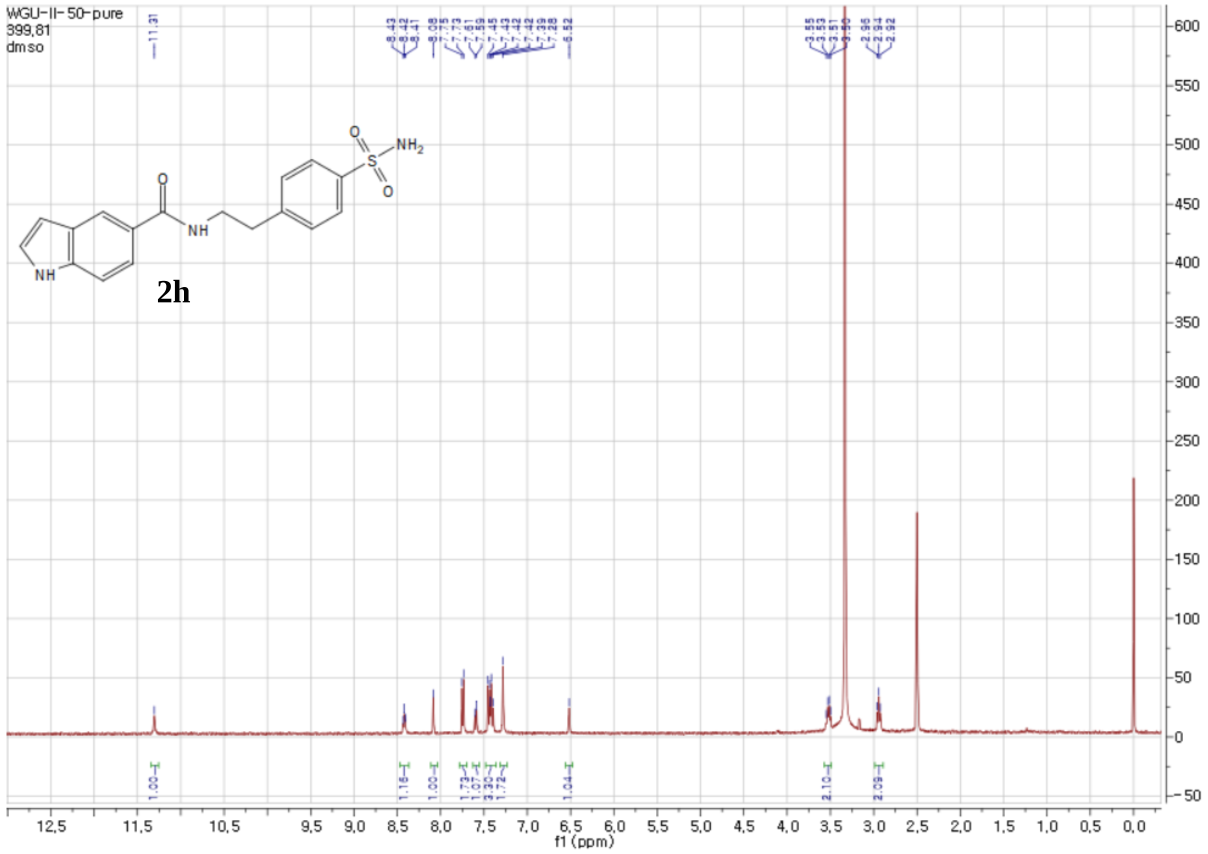
WGU-II-50-pure
399,81
dmso
2h
f1 (ppm)

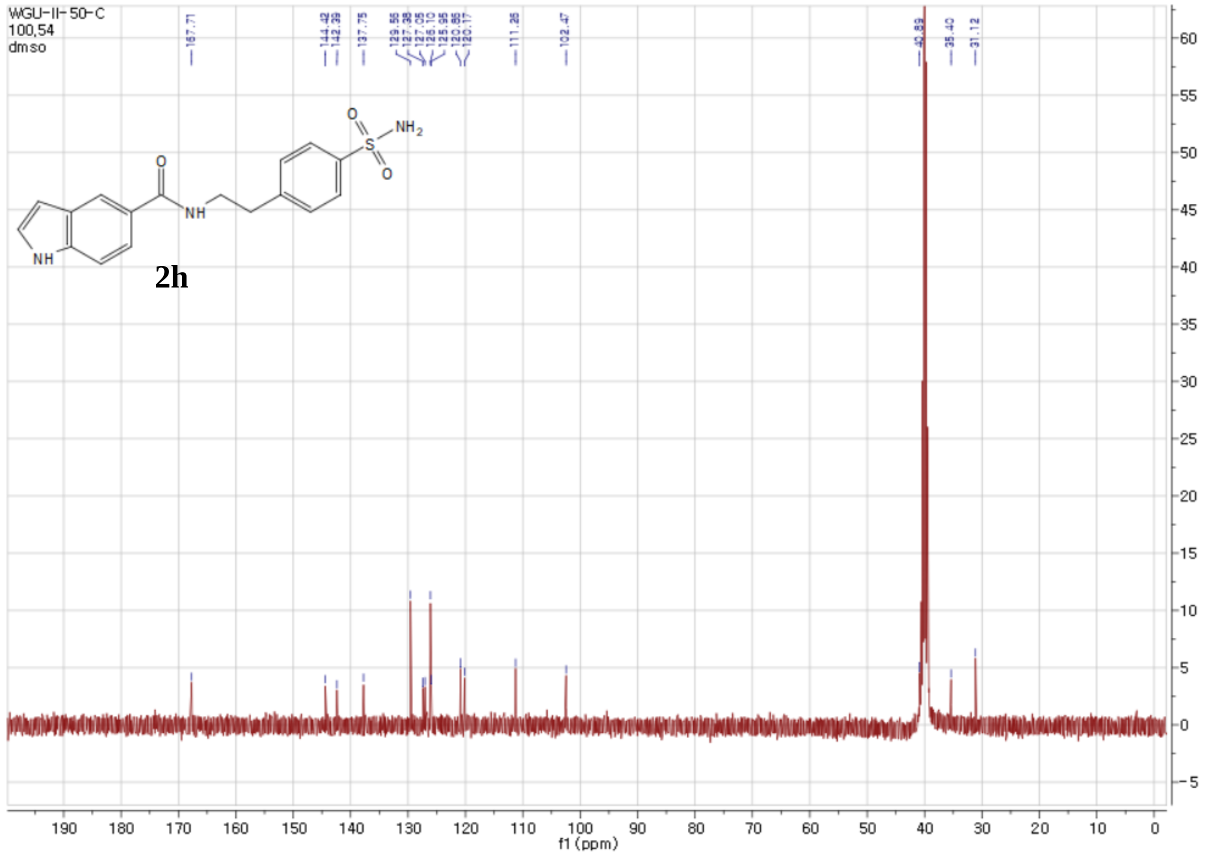
WGU-II-50-C
100,54
dmso
2h
f1 (ppm)

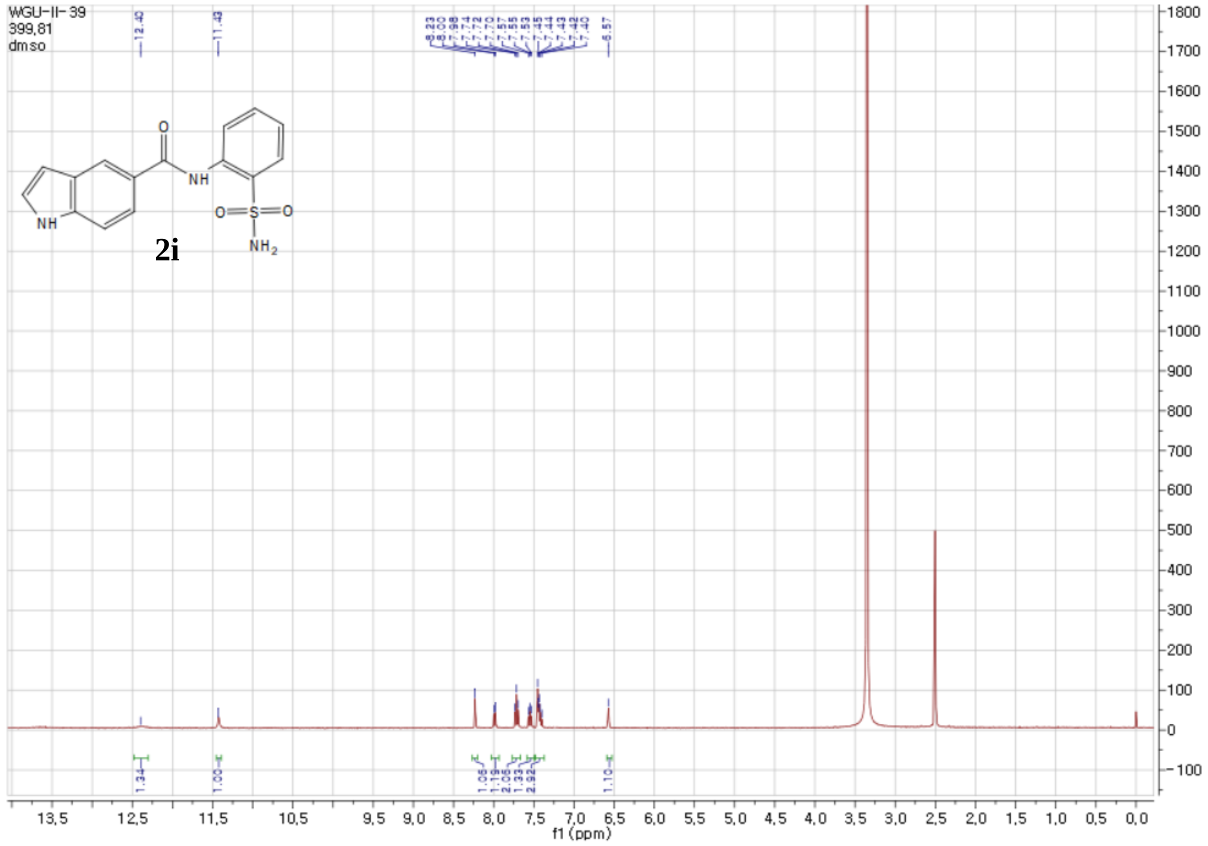
WGU-II-39
399,81
dmso
12.40
11.43
8.23
8.00
7.98
7.74
7.72
7.70
7.57
7.55
7.53
7.45
7.44
7.43
7.42
7.40
6.57
O
NH
NH
O=S=O
NH2
2i
1.34
1.00
1.06
1.19
2.06
1.33
2.92
1.10
f1 (ppm)

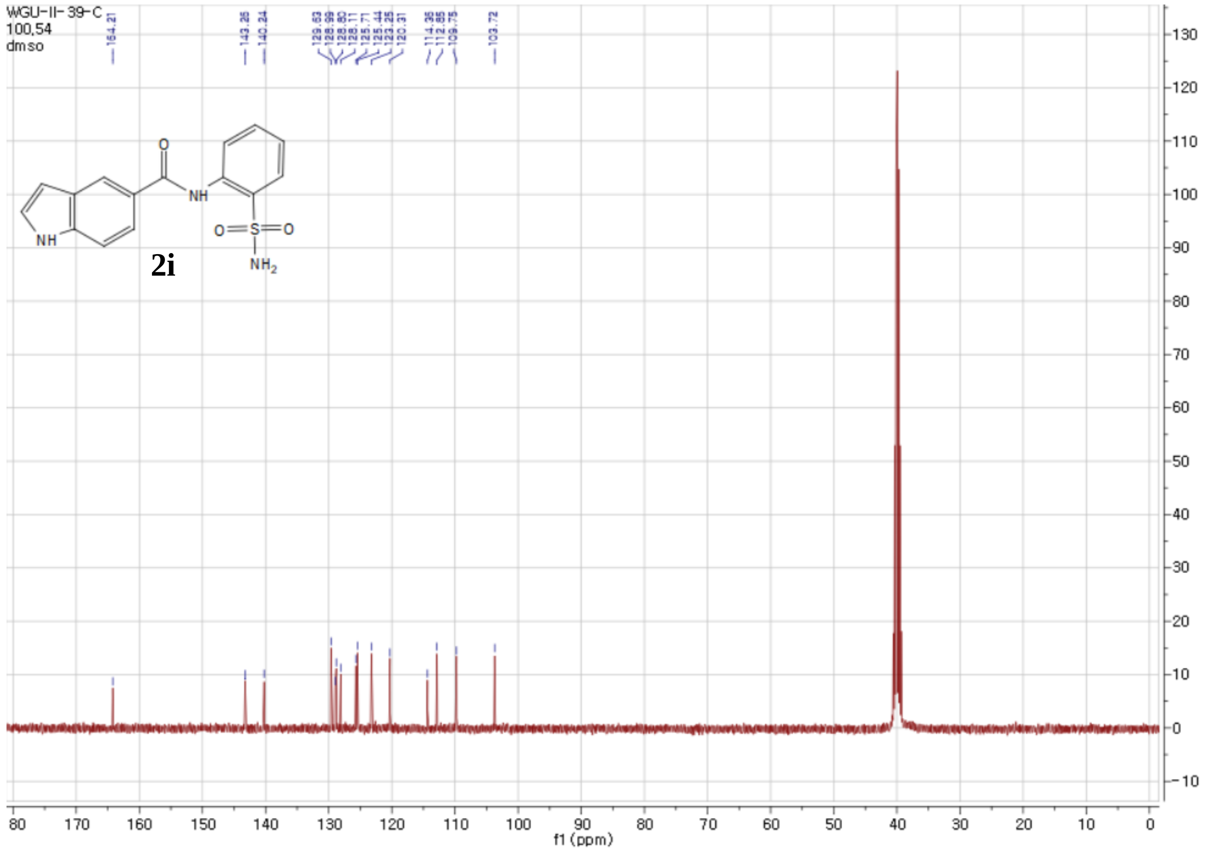
WGU-II-39-C
100,54
dmso
164.21
143.26
140.24
129.63
128.99
128.60
128.11
125.71
125.44
123.25
120.31
114.36
112.85
109.75
103.72
O
NH
NH
O=S=O
NH2
2i
f1 (ppm)

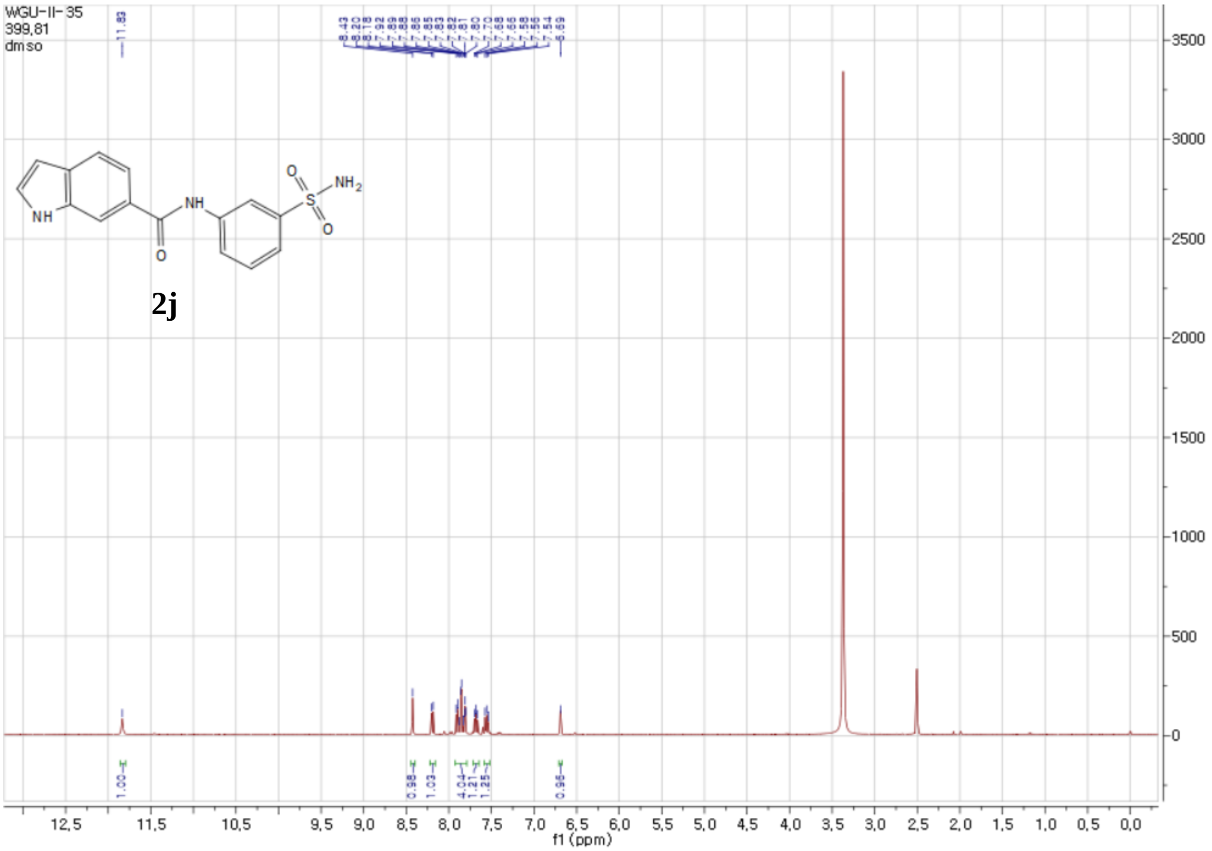
WGU-II-35
399,81
dmso
11.83
8.43 8.20 8.18 7.92 7.89 7.88 7.86 7.85 7.83 7.82 7.81 7.80 7.70 7.68 7.66 7.58 7.56 7.54 6.69
NH
NH
O
O
S
NH2
O
2j
1.00 0.98 1.03 4.04 1.21 1.25 0.96
f1 (ppm)

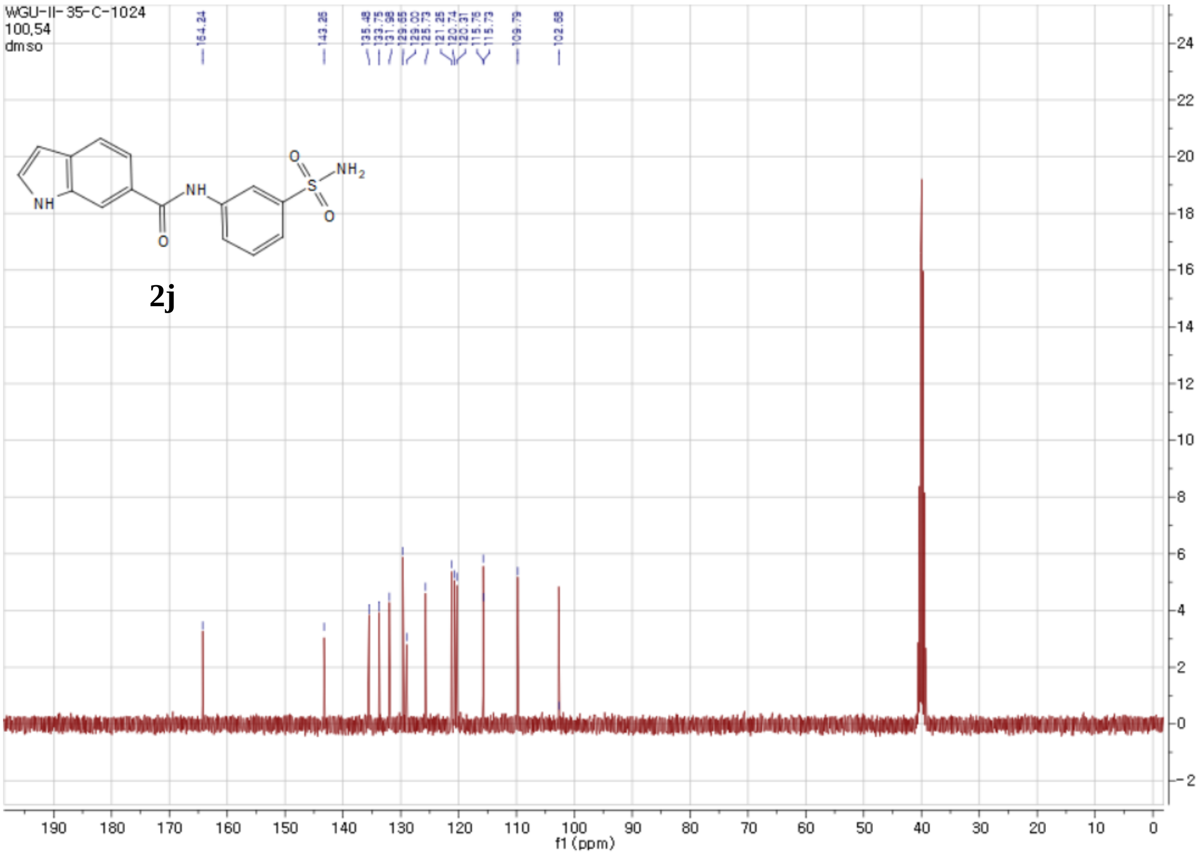
WGU-II-35-C-1024
100,54
dmso
164.24
143.26
135.48 133.75 131.98 129.65 129.00 125.73 121.25 120.74 120.31 115.76 115.73
109.79
102.68
NH
NH
O
O
S
NH2
O
2j
f1 (ppm)

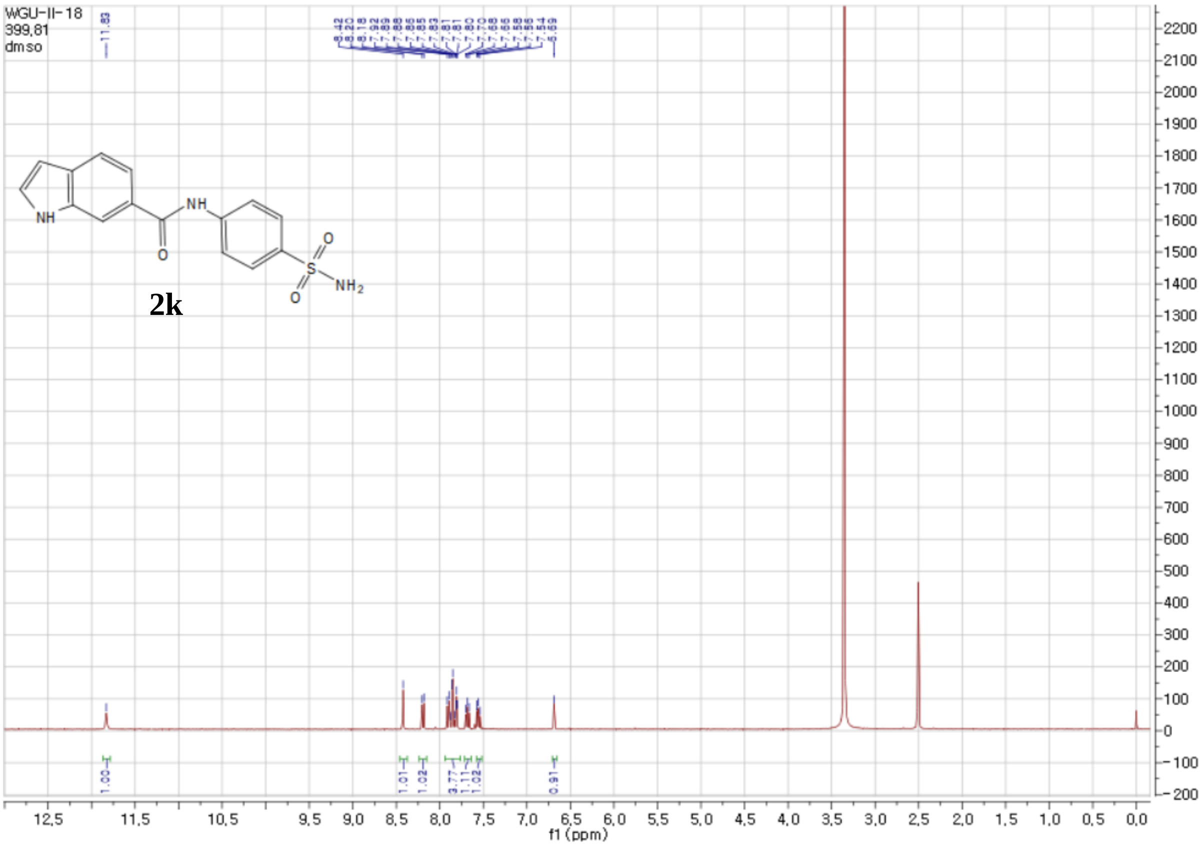
WGU-II-18
399,81
dmso
11.83
NH
NH
O
O
S
O
NH2
2k
1.00
1.01
1.02
3.77
1.11
1.02
0.91
f1 (ppm)

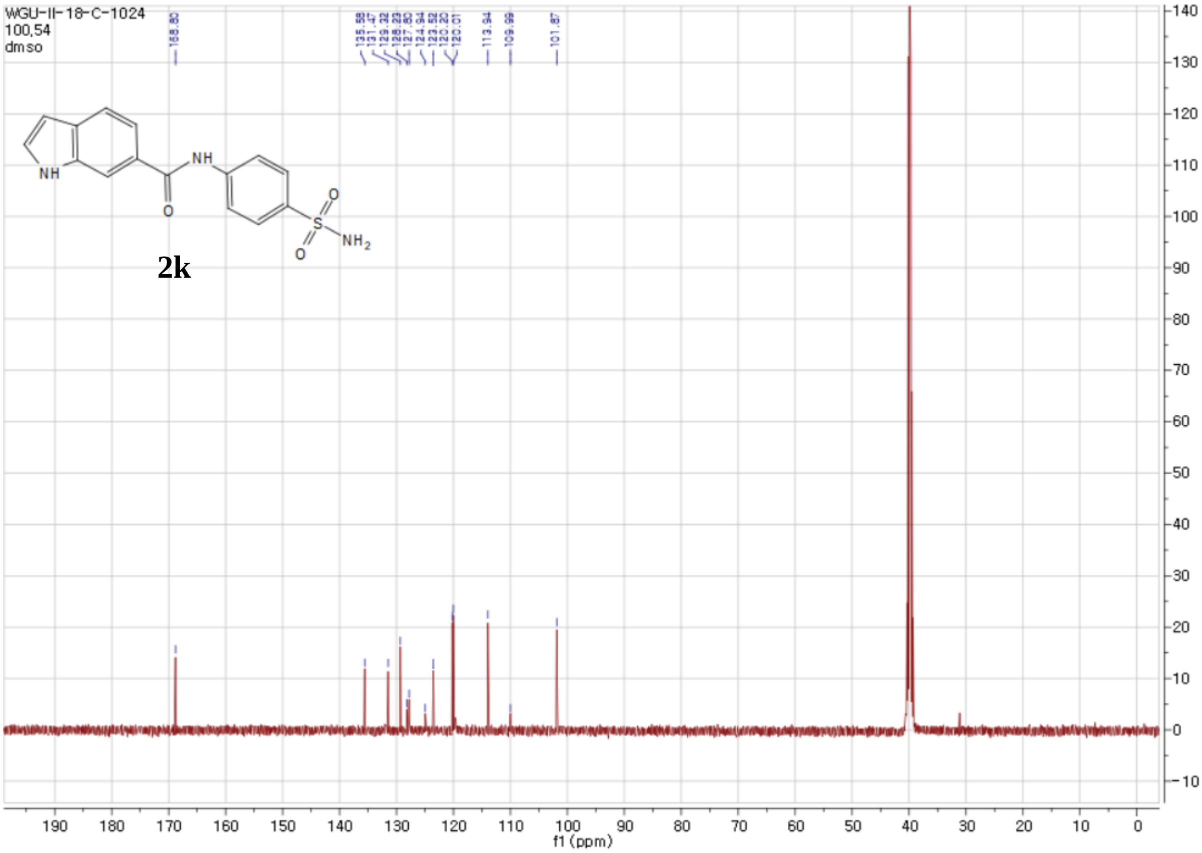
WGU-II-18-C-1024
100,54
dmso
168.85
135.58
131.47
129.32
128.23
127.80
124.94
123.52
120.20
120.01
113.94
109.99
101.87
NH
NH
O
O
S
O
NH2
2k
f1 (ppm)

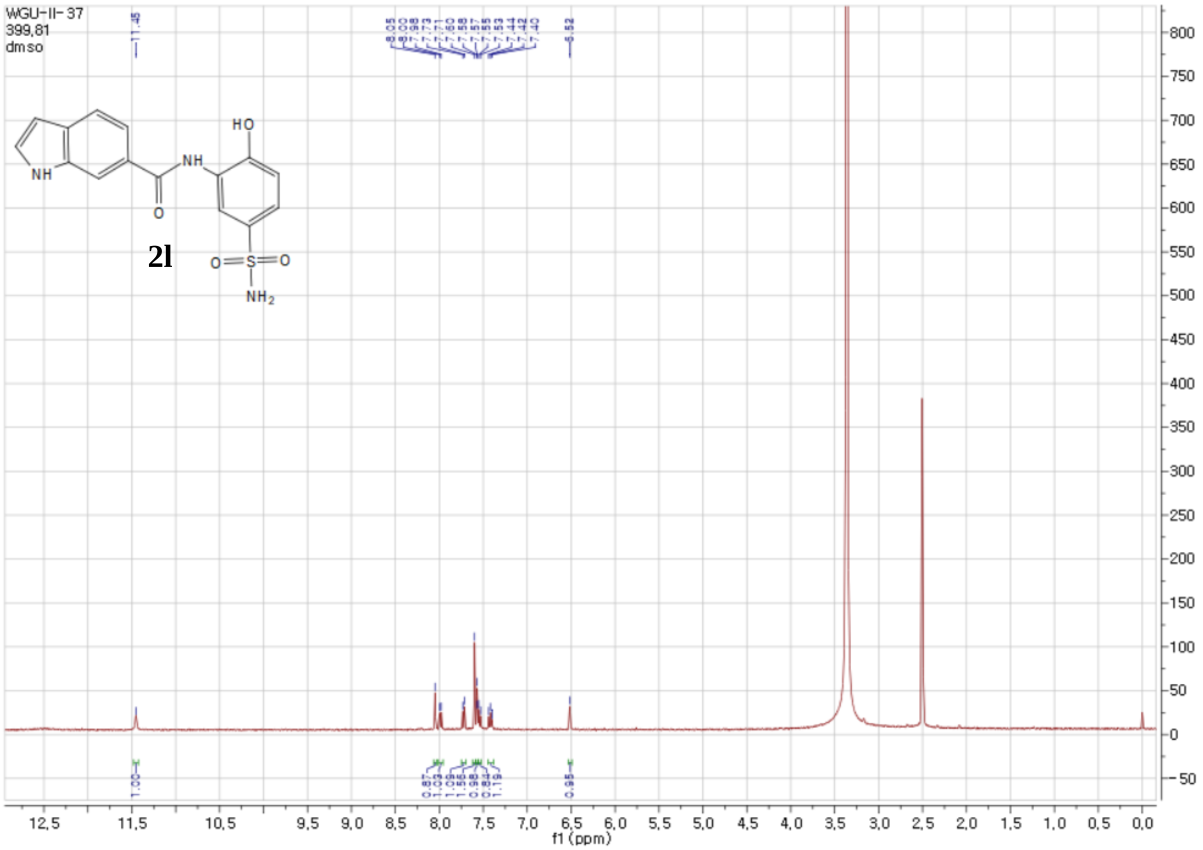
WGU-II-37
399,81
dmso
11.45
8.05 8.00 7.98 7.73 7.71 7.60 7.58 7.57 7.55 7.53 7.44 7.42 7.40
6.52
HO
NH
NH
O
O=S=O
NH2
2l
1.00
0.87 1.03 1.06 1.56 0.98 0.84 1.19
0.95
f1 (ppm)

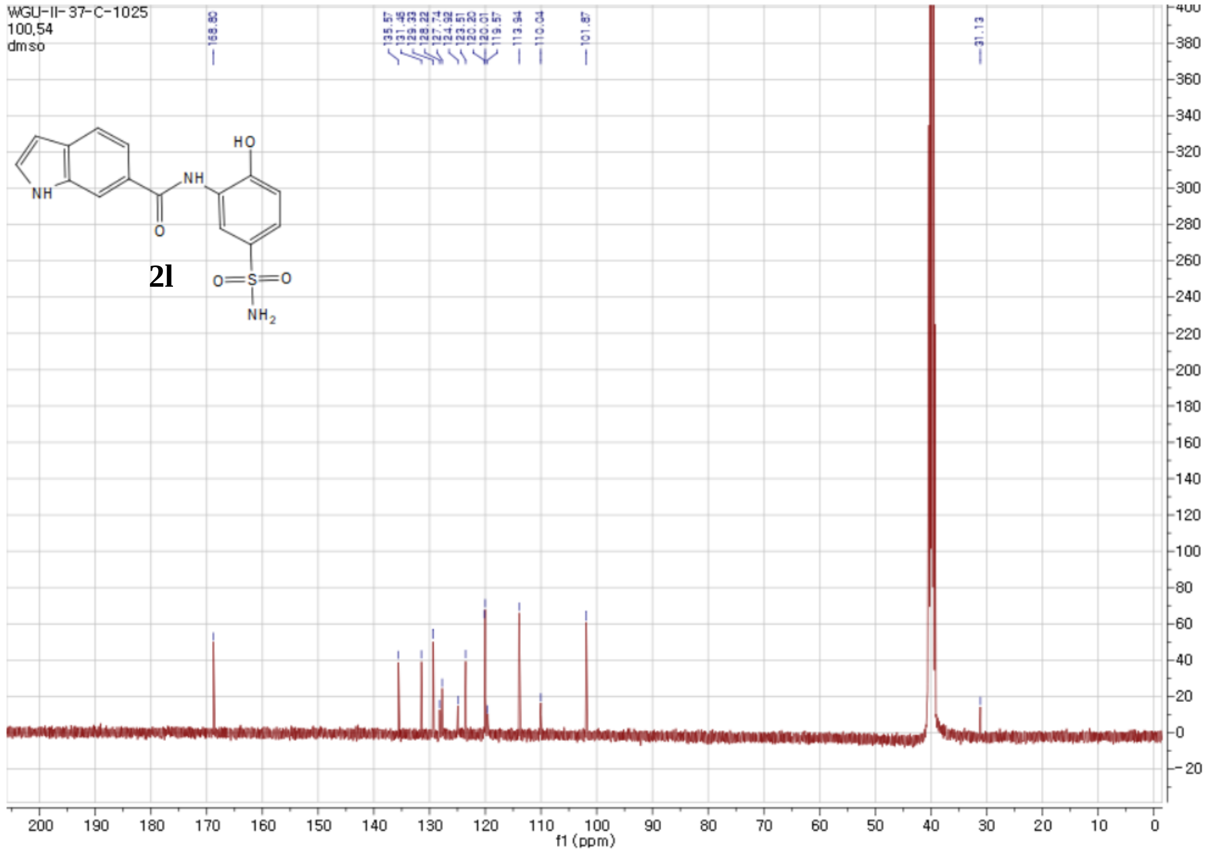
WGU-II-37-C-1025
100,54
dmso
168.80
135.57 131.45 129.33 128.22 127.74 124.92 123.51 120.20 120.01 119.57
113.94
110.04
101.87
31.13
HO
NH
NH
O
O=S=O
NH2
2l
f1 (ppm)

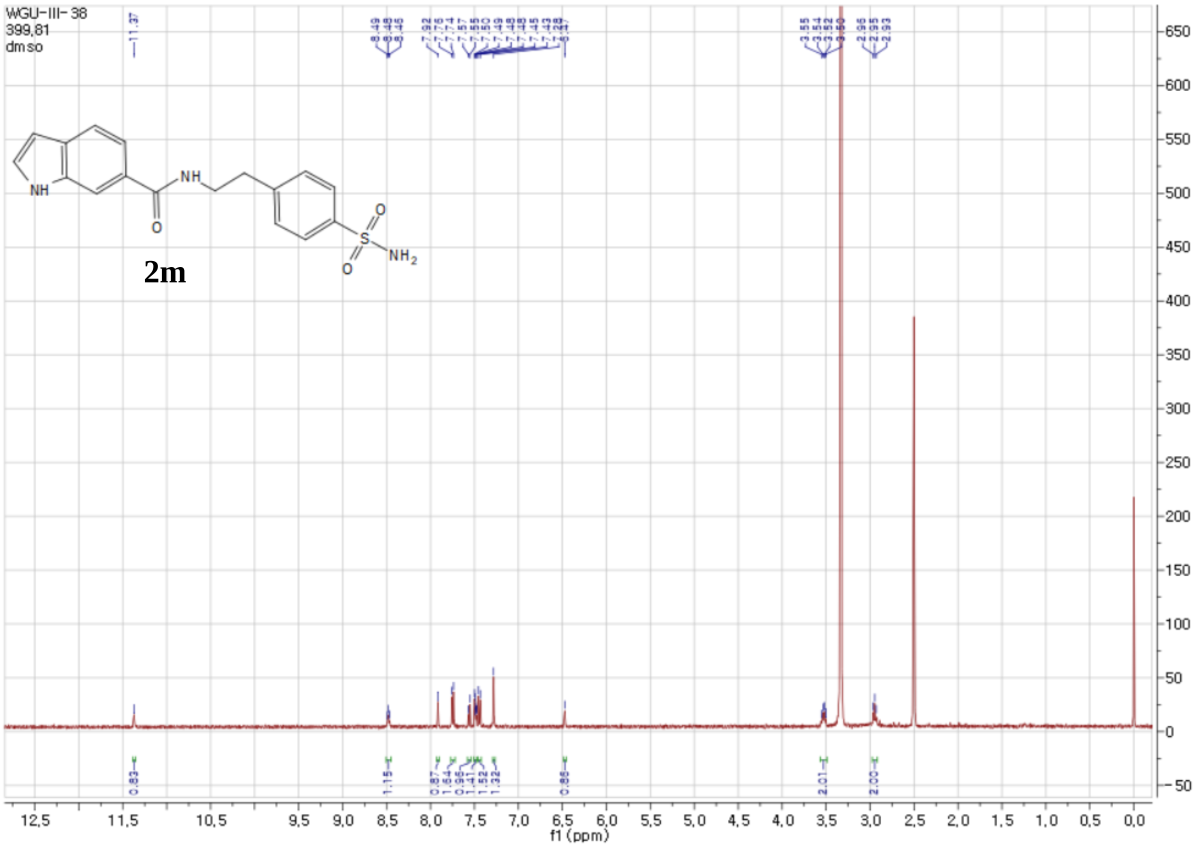
WGU-III-38
399,81
dmso
NH
NH
O
O
S
O
NH2
2m
f1 (ppm)

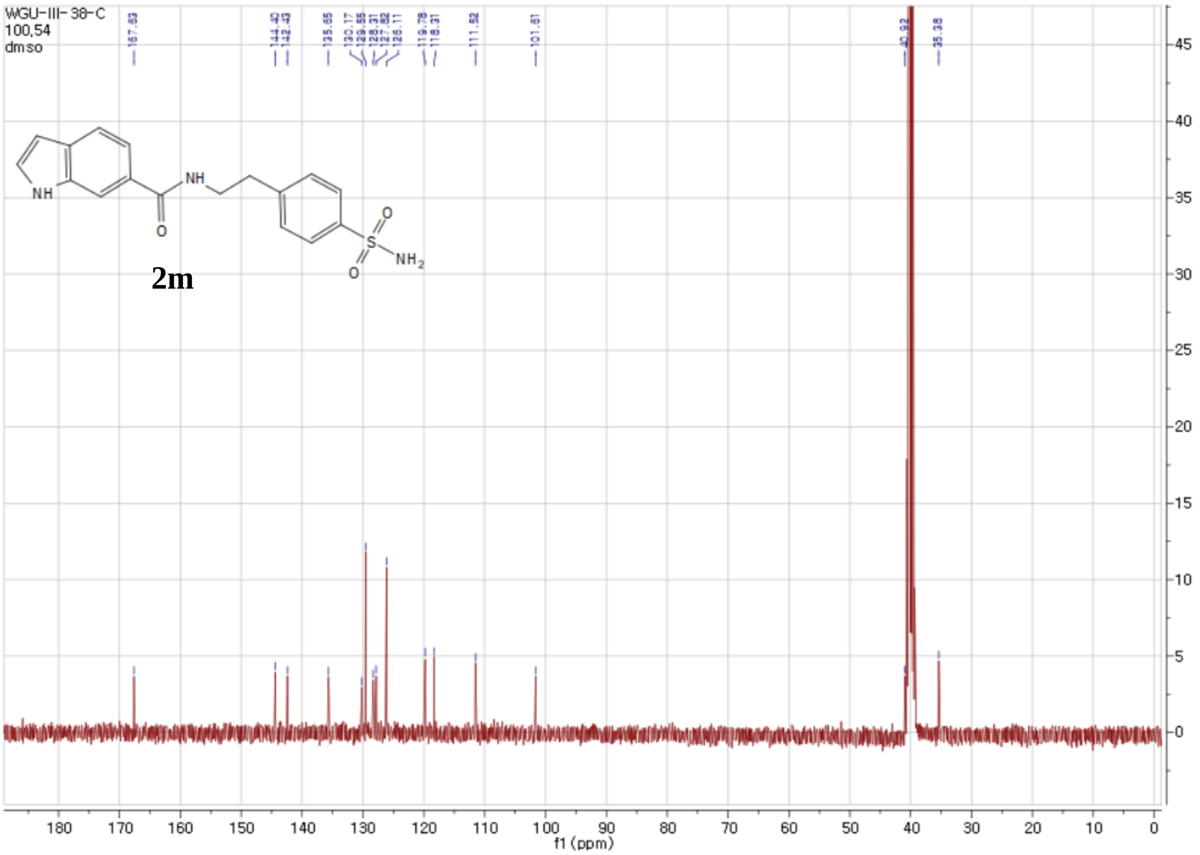
WGU-III-38-C
100,54
dmso
NH
NH
O
O
S
O
NH2
2m
f1 (ppm)

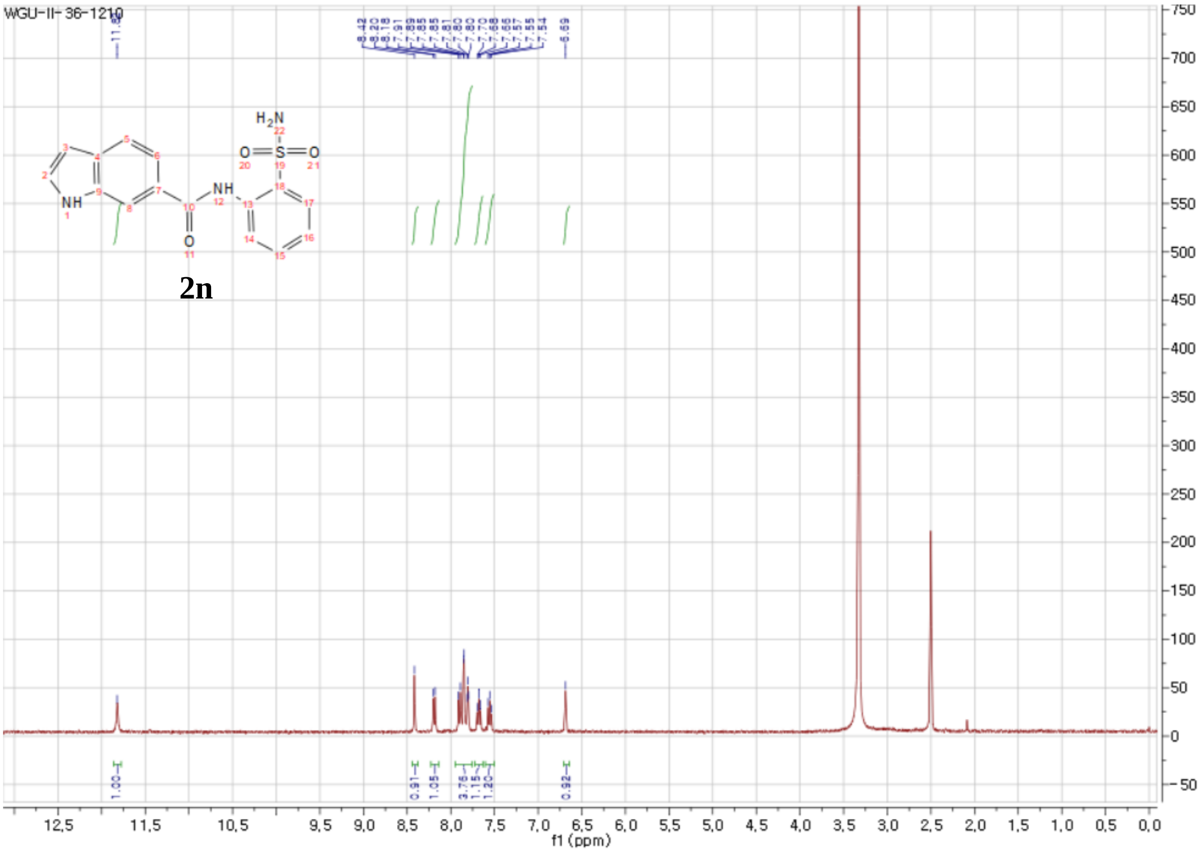
WGU-II-36-1210
H2N
NH
O
f1 (ppm)

2n

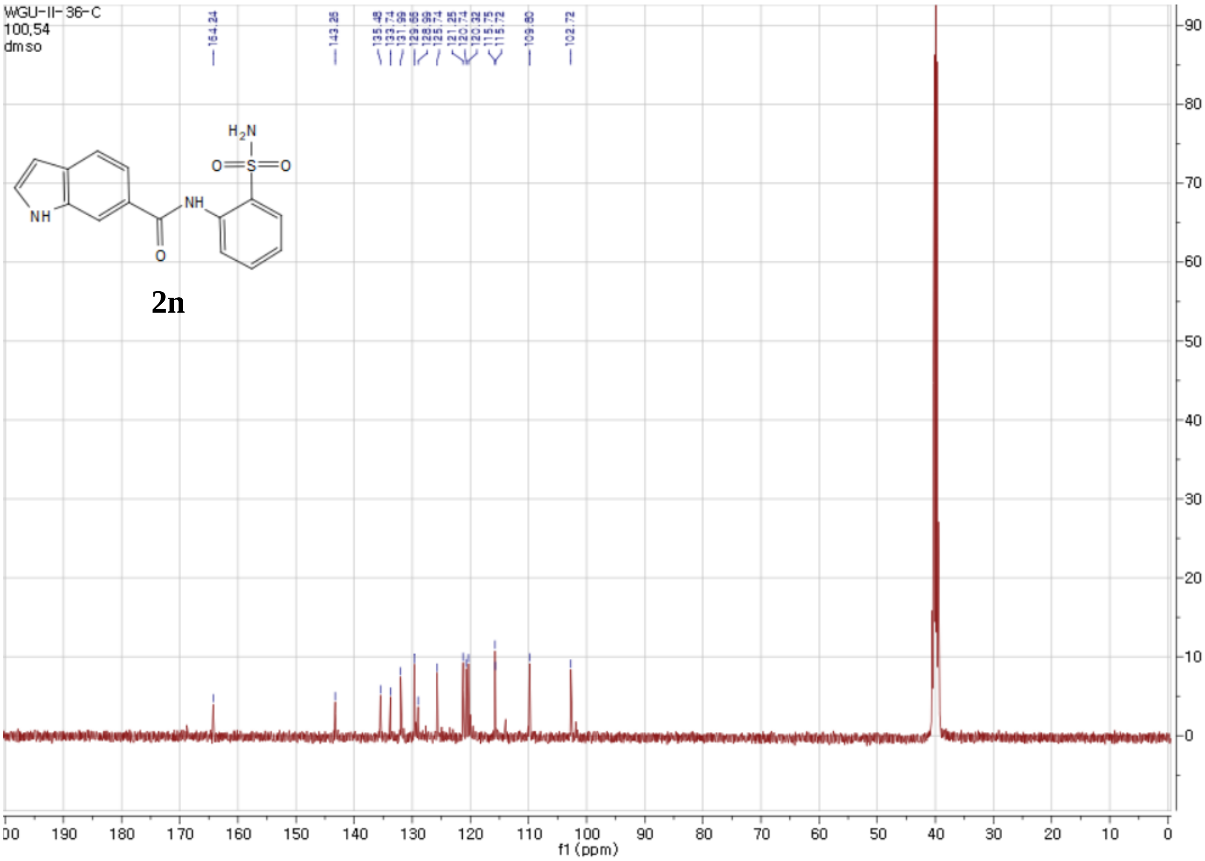
WGU-II-36-C
100,54
dmso
164.24
143.26
102.72
H2N
NH
O
f1 (ppm)

2n

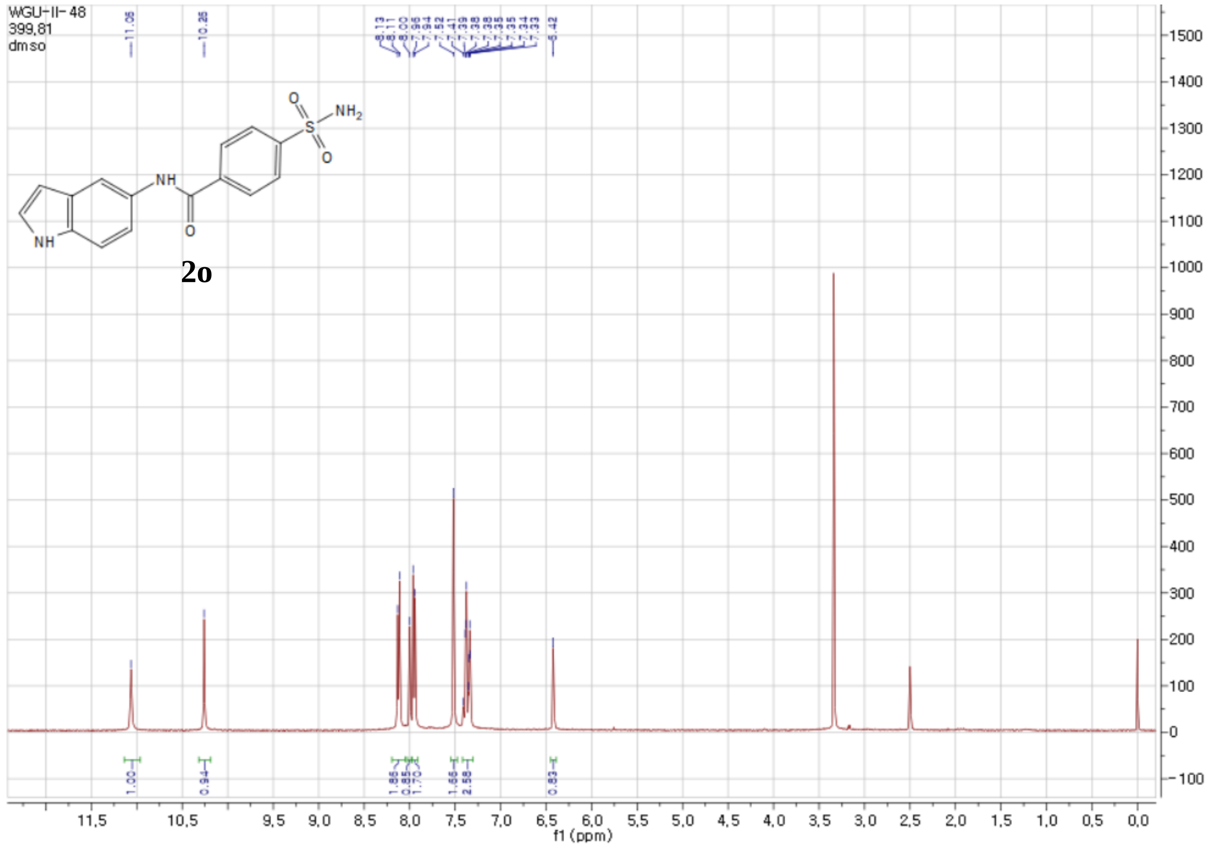
WGU-II-48
399.81
dmso
NH
NH
NH2
2o
f1 (ppm)

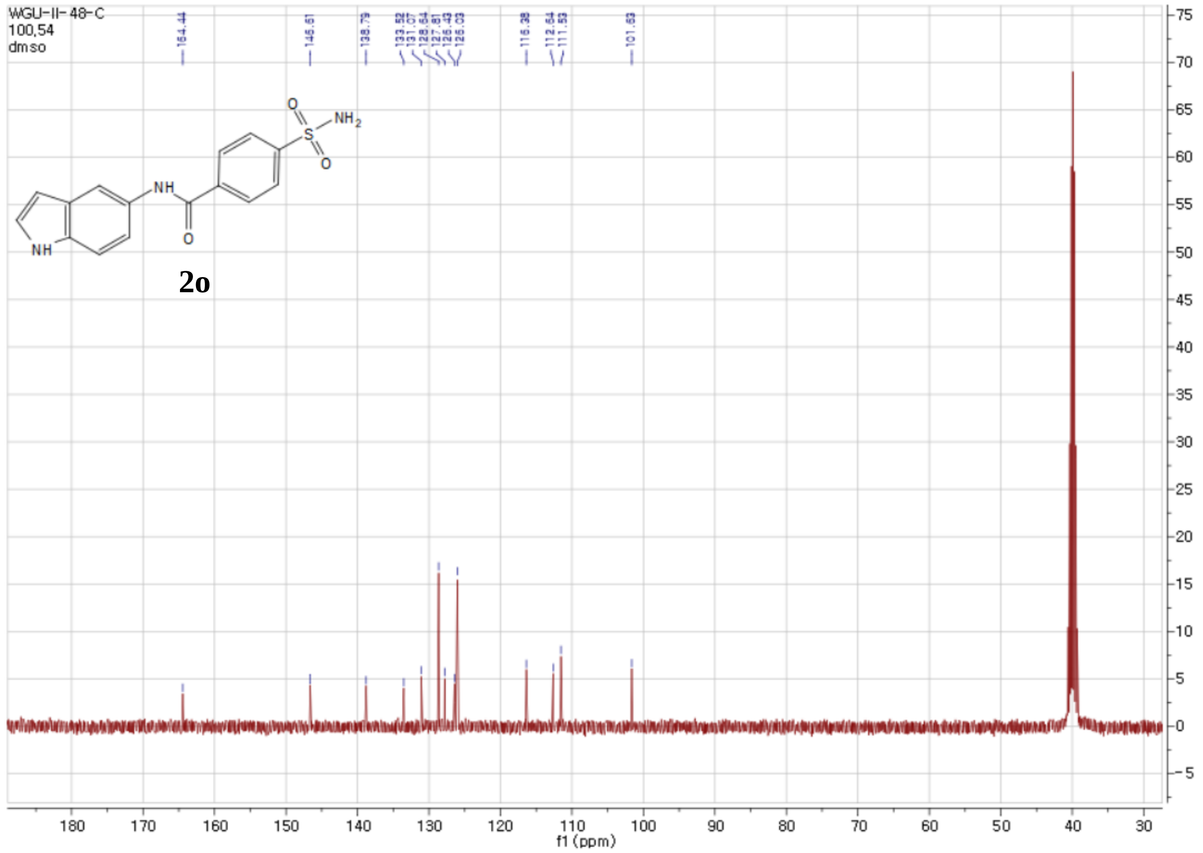
WGU-II-48-C
100.54
dmso
164.44
146.61
138.79
NH
NH
NH2
2o
f1 (ppm)

2. HPLC purity

	Name	Retention Time (min)	Purity1 Angle	Purity1 Threshold	PDA Match1 Spect. Name	PDA Match1 Angle	PDA Match1 Threshold	PDA Match1 Lib. Name	Area (µV*sec)	% Area	Height (µV)	Int Type	Amount
1		5.853							21570	0.21	4841	bb	
2		9.704							10154012	99.79	1640978	bb	

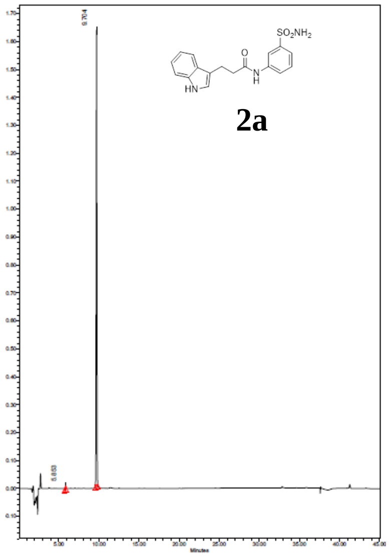

2a

	Name	Retention Time (min)	Purity1 Angle	Purity1 Threshold	PDA Match1 Spect. Name	PDA Match1 Angle	PDA Match1 Threshold	PDA Match1 Lib. Name	Area (µV*sec)	% Area	Height (µV)	Int Type	Amount
1		3.160							35909	0.23	2232	bb	
2		4.933							48324	0.30	4938	bb	
3		6.038							18831	0.12	2398	bb	
4		6.700							1569	0.01	423	bb	
5		7.249							7081	0.04	1689	bb	
6		8.238							44044	0.28	8594	bb	
7		8.659							15369532	97.00	2242742	bb	
8		10.077							2482	0.02	311	bb	
9		11.383							40538	0.26	5556	bb	
10		12.766							17021	0.11	4020	bb	
11		13.641							1431	0.01	382	bb	
12		15.298							257785	1.63	15769	bb	

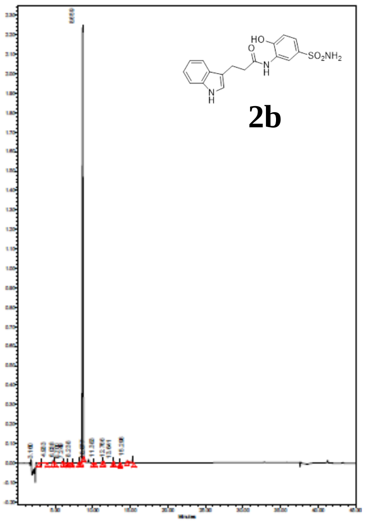

2b

	Name	Retention Time (min)	Purity1 Angle	Purity1 Threshold	PDA Match1 Spect. Name	PDA Match1 Angle	PDA Match1 Threshold	PDA Match1 Lib. Name	Area (µV*sec)	% Area	Height (µV)	Int Type	Amount	Units
1		12.265							909	0.06	253	bb		
2		13.882							1591817	99.00	286374	bb		
3		14.943							525	0.03	199	bb		
4		15.868							0	0.00	-0	bb		
5		16.350							376	0.02	87	bb		
6		17.441							628	0.04	145	bb		
7		18.177							880	0.05	-125	bb		
8		18.965							6813	0.42	563	bb		
9		20.030							5964	0.37	1217	bb		

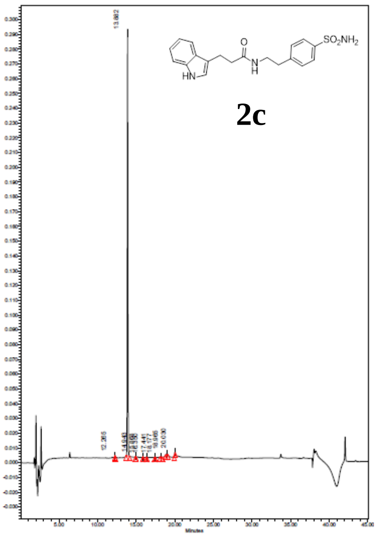

	Name	Retention Time (min)	Purity1 Angle	Purity1 Threshold	PDA Match1 Spect. Name	PDA Match1 Angle	PDA Match1 Threshold	PDA Match1 Lib. Name	Area (µV*sec)	% Area	Height (µV)	Int Type	Amount
1		9.741							16354019	99.98	2322914	bb	
2		12.437							2751	0.02	812	bb	

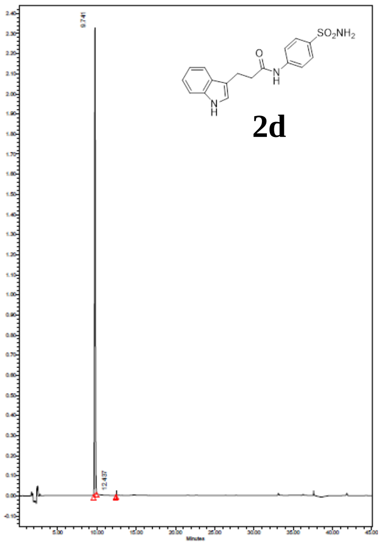

	Name	Retention Time (min)	Purity1 Angle	Purity1 Threshold	PDA Match1 Spect. Name	PDA Match1 Angle	PDA Match1 Threshold	PDA Match1 Lib. Name	Area (µV*sec)	% Area	Height (µV)	Int Type	Amount
1		12.045							13607	0.06	2636	bb	
2		15.123							23033597	96.78	2409116	bb	
3		20.434							752736	3.16	120461	bb	

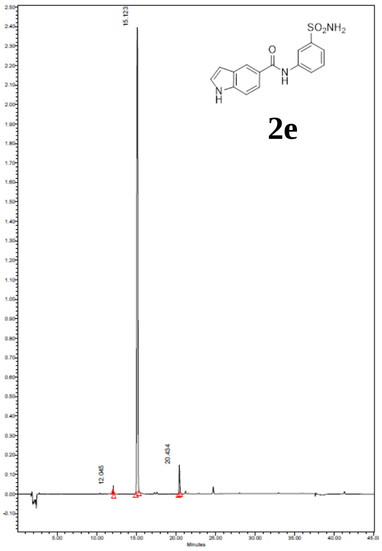

2e

	Name	Retention Time (min)	Purity1 Angle	Purity1 Threshold	PDA Match1 Spect. Name	PDA Match1 Angle	PDA Match1 Threshold	PDA Match1 Lib. Name	Area (µV*sec)	% Area	Height (µV)	Int Type	Amount
1		15.051							26567282	100.00	2436018	bb	

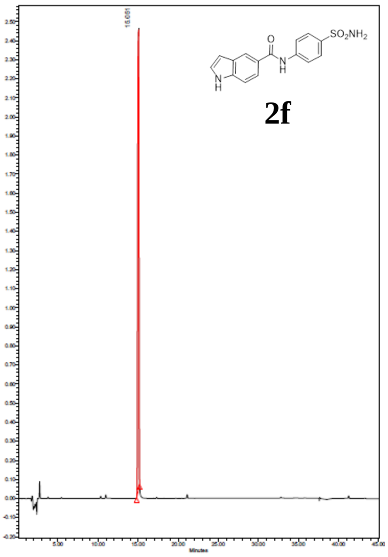

2f

	Name	Retention Time (min)	Purity1 Angle	Purity1 Threshold	PDA Match1 Spect. Name	PDA Match1 Angle	PDA Match1 Threshold	PDA Match1 Lib. Name	Area (µV*sec)	% Area	Height (µV)	Int Type	Amount
1		11.039							40642	0.21	7378	bb	
2		15.087							19368165	99.62	2407029	bb	
3		21.195							33647	0.17	5326	bb	

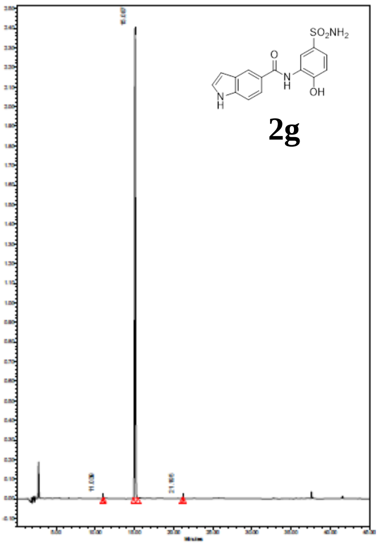

	Name	Retention Time (min)	Purity1 Angle	Purity1 Threshold	PDA Match1 Spect. Name	PDA Match1 Angle	PDA Match1 Threshold	PDA Match1 Lib. Name	Area (µV*sec)	% Area	Height (µV)	Int Type	Amount
1		5.124							14862	0.10	3240	bb	
2		5.406							224896	1.56	39342	bb	
3		6.140							14058338	97.68	2113810	bb	
4		9.788							22932	0.16	6528	bb	
5		12.337							70658	0.49	17520	bb	

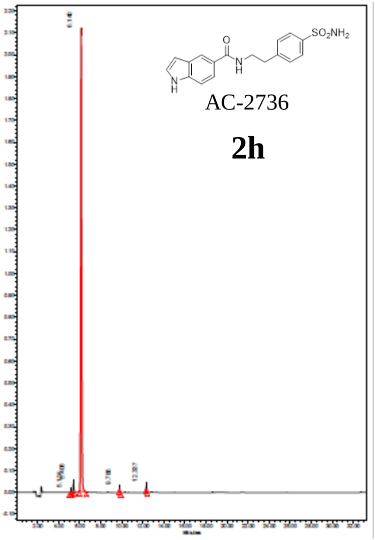

	Name	Retention Time (min)	Purity1 Angle	Purity1 Threshold	PDA Match1 Spect. Name	PDA Match1 Angle	PDA Match1 Threshold	PDA Match1 Lib. Name	Area (μV*sec)	% Area	Height (μV)	Int Type	Amount
1		9.104							54155	0.66	12032	bb	
2		9.748							764	0.01	240	bb	
3		11.355							52092	0.64	10110	bb	
4		11.797							1827	0.02	501	bb	
5		12.212							13261	0.16	2847	bb	
6		12.748							61	0.00	61	bb	
7		13.714							1949	0.02	617	bb	
8		15.972							8014764	98.02	1163330	bb	
9		16.641							37974	0.46	4672	bb	

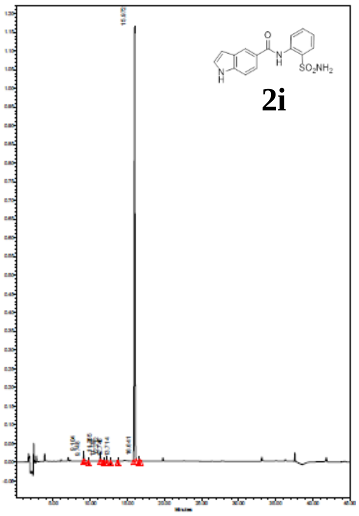

	Name	Retention Time (min)	Purity1 Angle	Purity1 Threshold	PDA Match1 Spect. Name	PDA Match1 Angle	PDA Match1 Threshold	PDA Match1 Lib. Name	Area (μV*sec)	% Area	Height (μV)	Int Type	Amount
1		11.070							29879	0.15	4607	bb	
2		12.015							25660	0.13	-4915	bb	
3		15.723							20068158	99.67	2419202	bb	
4		19.557							10109	0.05	1649	bb	

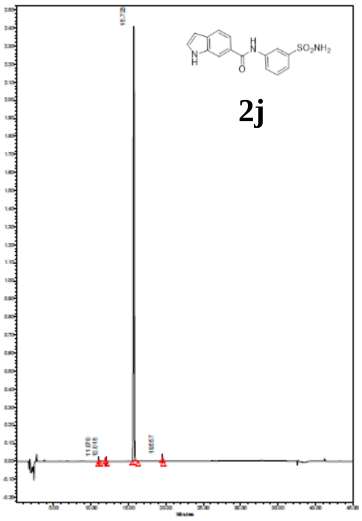

	Name	Retention Time (min)	Purity1 Angle	Purity1 Threshold	PDA Match1 Spect. Name	PDA Match1 Angle	PDA Match1 Threshold	PDA Match1 Lib. Name	Area (µV*sec)	% Area	Height (µV)	Int Type	Amount
1		12.191							1763	0.02	705	bb	
2		12.434							6031	0.08	1412	bb	
3		15.953							7503939	99.90	1092658	bb	

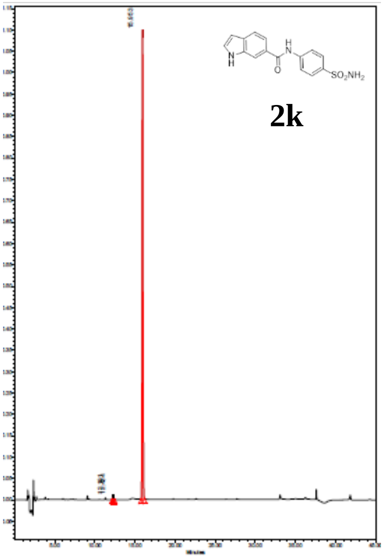

2k

	Name	Retention Time (min)	Purity1 Angle	Purity1 Threshold	PDA Match1 Spect. Name	PDA Match1 Angle	PDA Match1 Threshold	PDA Match1 Lib. Name	Area (µV*sec)	% Area	Height (µV)	Int Type	Amount
1		6.041							11743	0.18	2638	bb	
2		6.995							3264	0.05	681	bb	
3		7.281							8370	0.13	1922	bb	
4		7.773							11908	0.19	1654	bb	
5		9.094							50125	0.78	10364	bb	
6		9.709							7152	0.11	1110	bb	
7		11.344							14838	0.23	2412	bb	
8		12.201							2902	0.05	849	bb	
9		13.144							7235	0.11	-585	bb	
10		13.919							269	0.00	-56	bb	
11		15.492							27	0.00	-14	bb	
12		15.608							0	0.00	0	bb	
13		15.963							6272192	98.03	918650	bb	

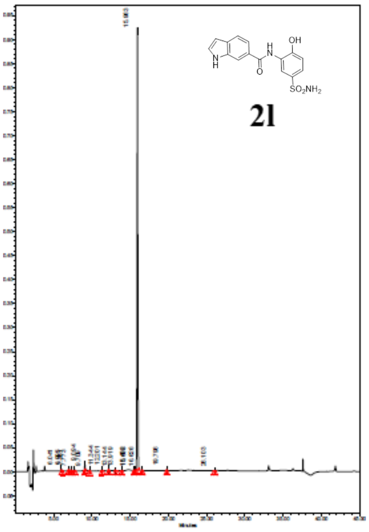

2l

	Name	Retention Time (min)	Purity1 Angle	Purity1 Threshold	PDA Match1 Spect. Name	PDA Match1 Angle	PDA Match1 Threshold	PDA Match1 Lib. Name	Area (µV*sec)	% Area	Height (µV)	Int Type	Amount	Units
1		1.982							1737170	96.73	129132	bb		
2		2.407							1179	0.07	597	bb		
3		2.518							55830	3.11	13988	bb		
4		2.752							1686	0.09	558	bb		

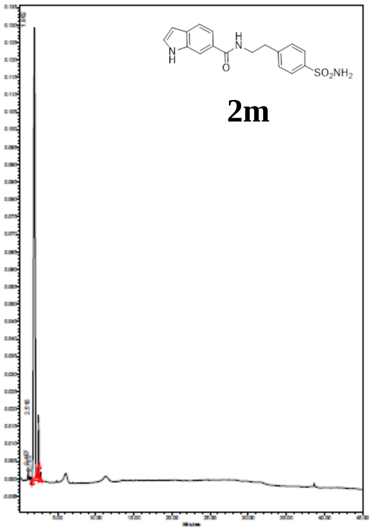

2m

	Name	Retention Time (min)	Purity1 Angle	Purity1 Threshold	PDA Match1 Spect. Name	PDA Match1 Angle	PDA Match1 Threshold	PDA Match1 Lib. Name	Area (µV*sec)	% Area	Height (µV)	Int Type	Amount
1		9.092							76112	0.92	13853	bb	
2		9.338							307	0.00	192	bb	
3		9.714							6898	0.08	1735	bb	
4		11.336							18297	0.22	3916	bb	
5		12.196							2351	0.03	702	bb	
6		15.238							34	0.00	34	bb	
7		15.954							8184772	98.70	1190180	bb	
8		16.619							4039	0.05	793	bb	

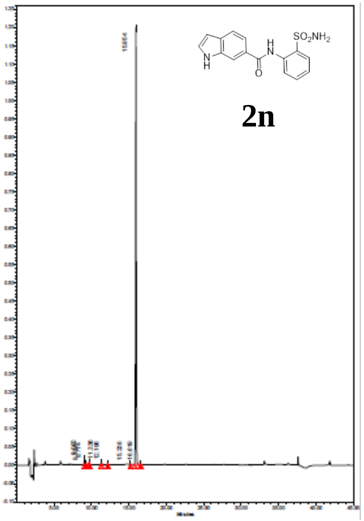

2n

	Name	Retention Time (min)	Purity1 Angle	Purity1 Threshold	PDA Match1 Spect. Name	PDA Match1 Angle	PDA Match1 Threshold	PDA Match1 Lib. Name	Area (μV*sec)	% Area	Height (μV)	Int Type	Amount
1		6.655							16039540	97.38	2313113	bb	
2		7.778							9441	0.06	1850	bb	
3		8.891							6264	0.04	-1087	bb	
4		9.898							415866	2.52	66089	bb	

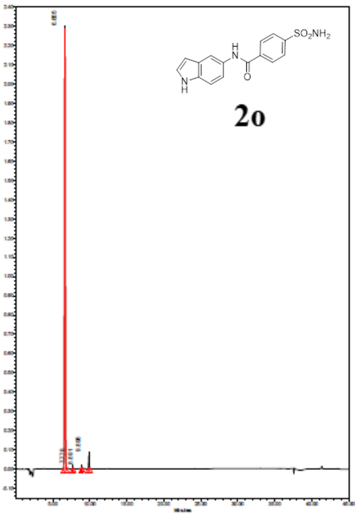

3. HRMS data

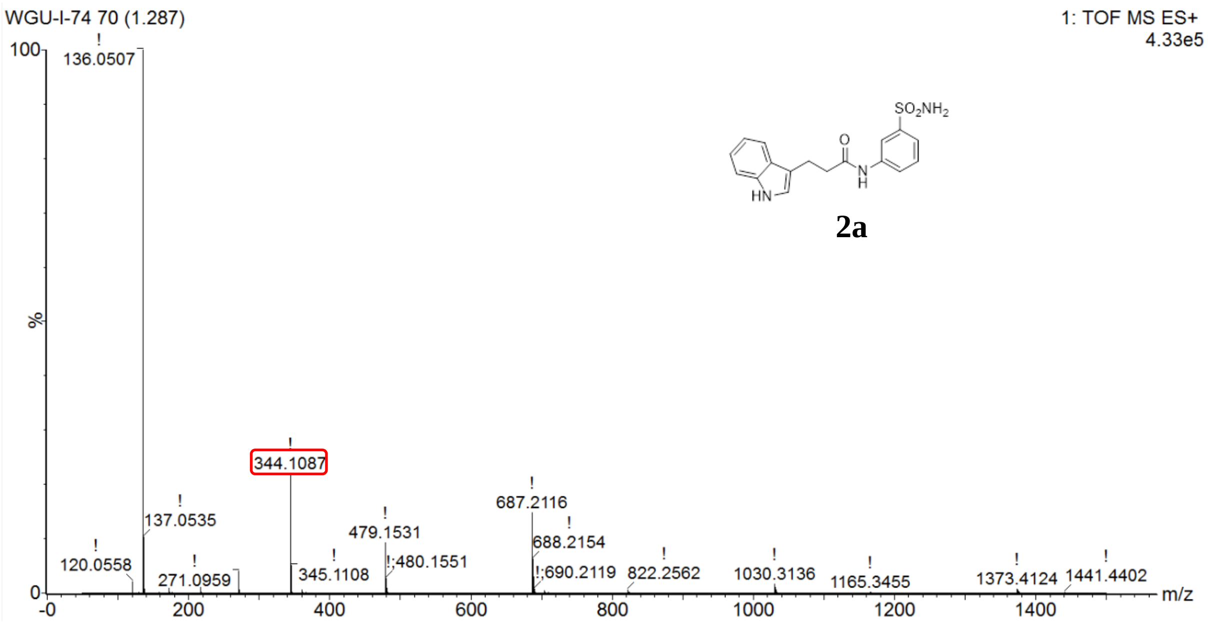

2a

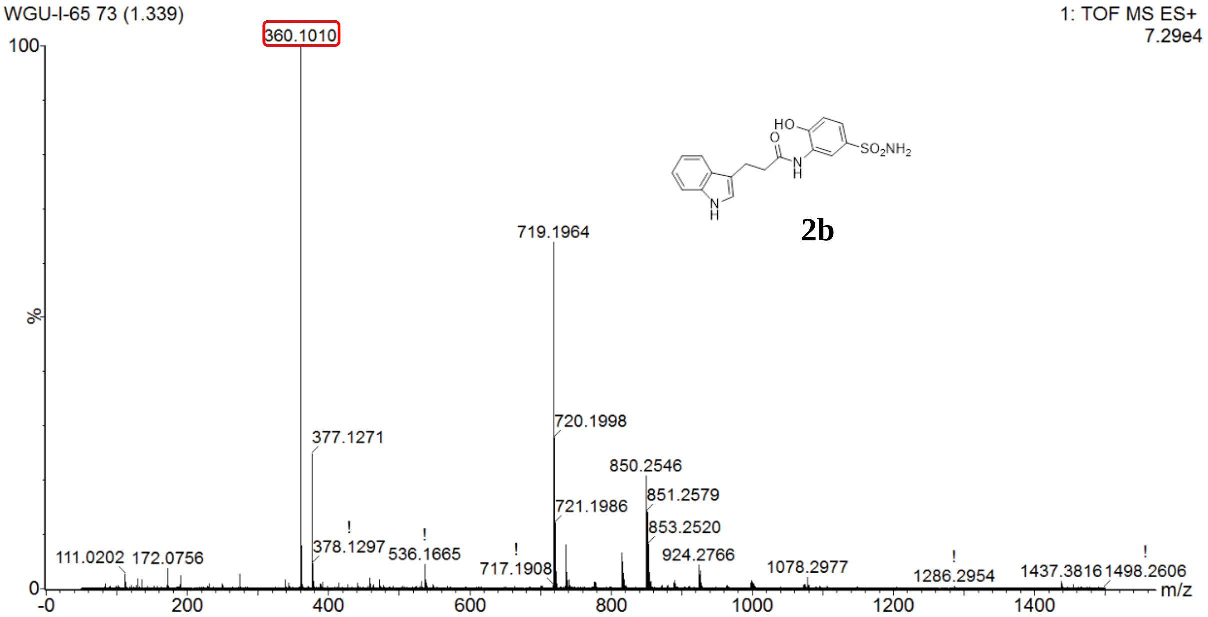

2b

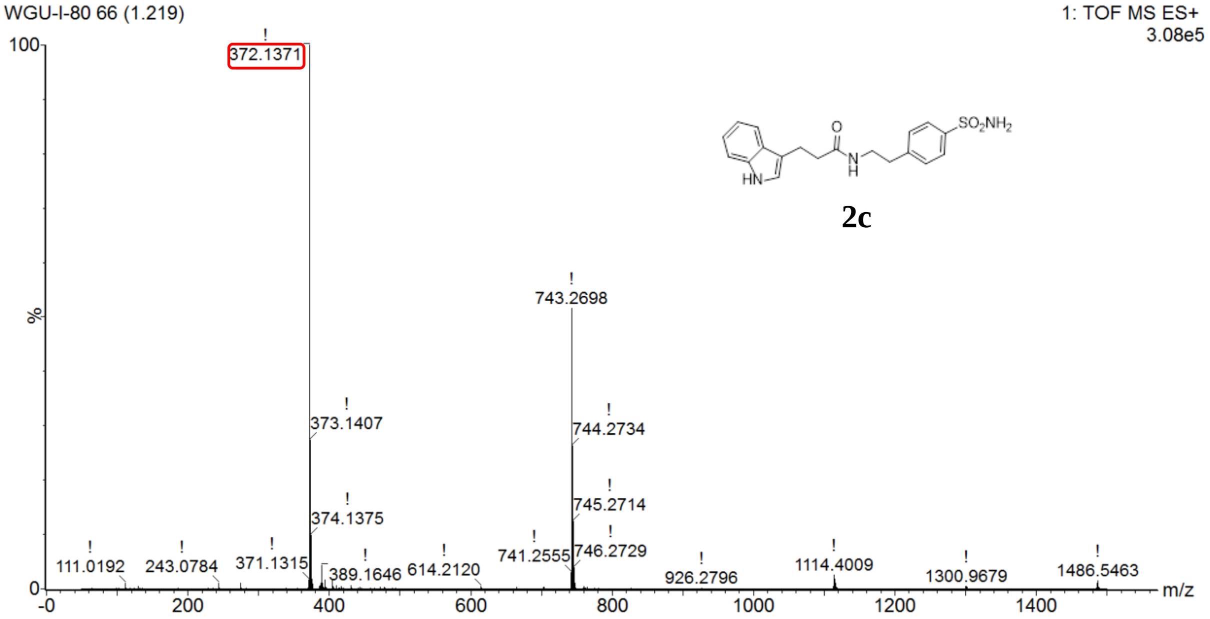
WGU-I-80 66 (1.219)
1: TOF MS ES+
3.08e5
100
%
0
372.1371
743.2698
373.1407
744.2734
374.1375
745.2714
111.0192
243.0784
371.1315
389.1646
614.2120
741.2555
746.2729
926.2796
1114.4009
1300.9679
1486.5463
-0
200
400
600
800
1000
1200
1400
m/z
SO_2NH_2
HN
O
N
H
2c

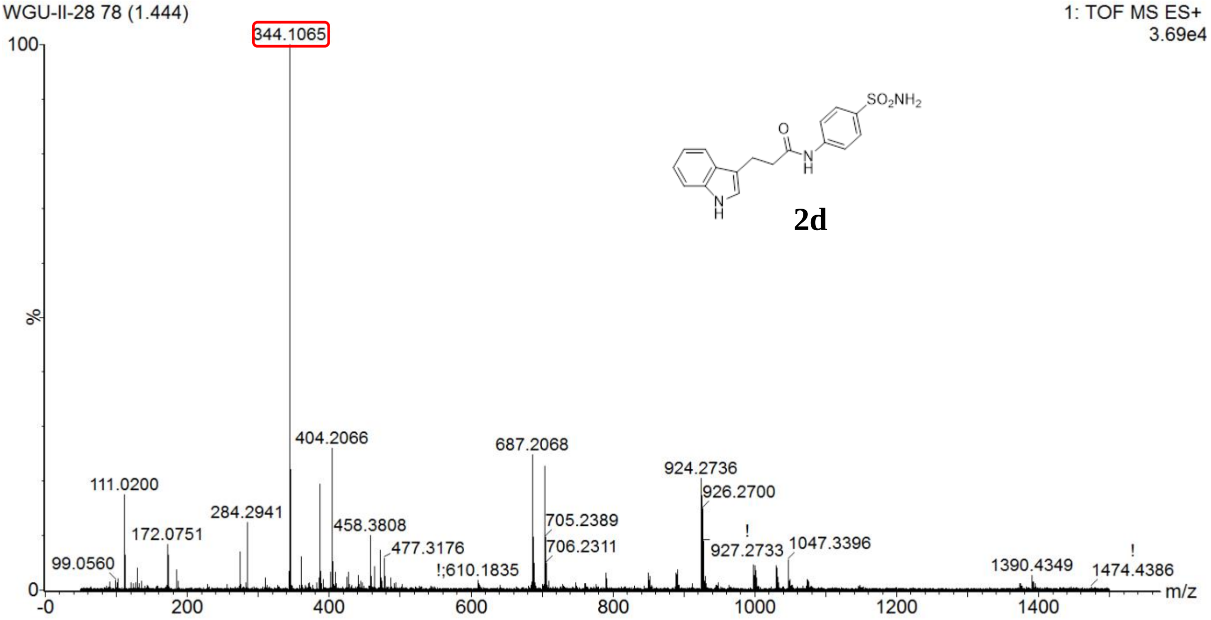
WGU-II-28 78 (1.444)
1: TOF MS ES+
3.69e4
100
%
0
344.1065
404.2066
687.2068
111.0200
284.2941
924.2736
926.2700
172.0751
458.3808
705.2389
477.3176
706.2311
927.2733
1047.3396
99.0560
610.1835
1390.4349
1474.4386
-0
200
400
600
800
1000
1200
1400
m/z
SO_2NH_2
O
N
H
N
H
2d

Compound 2e (Fragmentation)

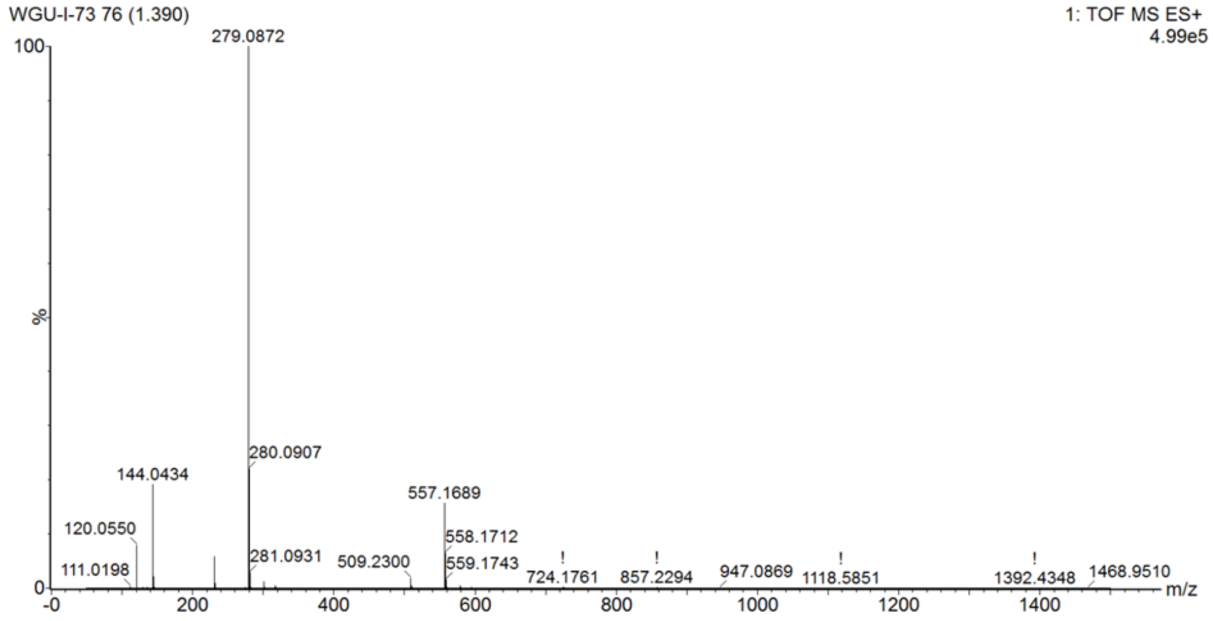

Compound 2f (Fragmentation)

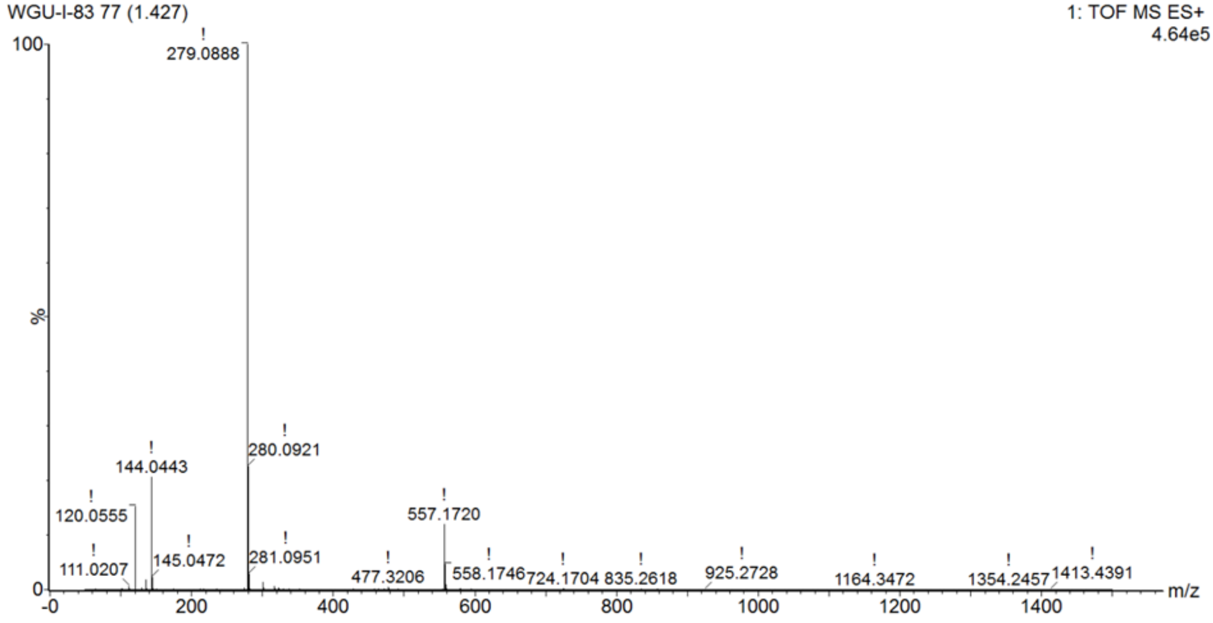

Compound 2g

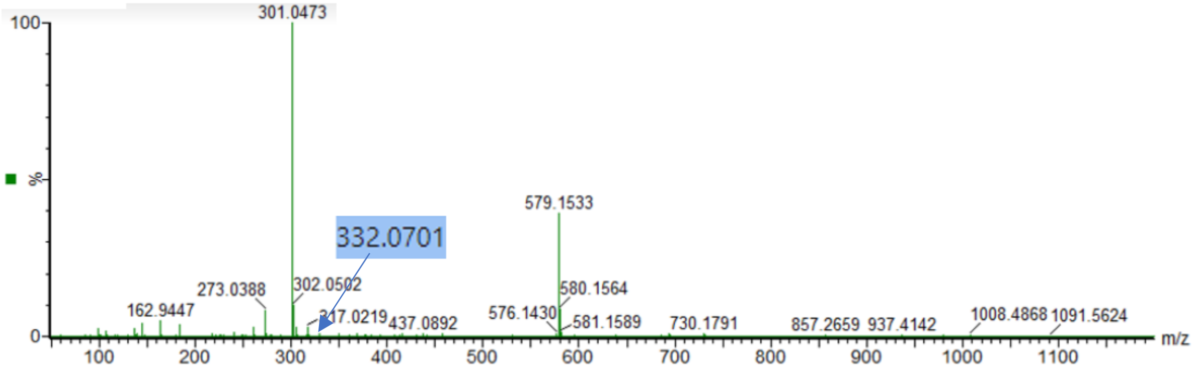

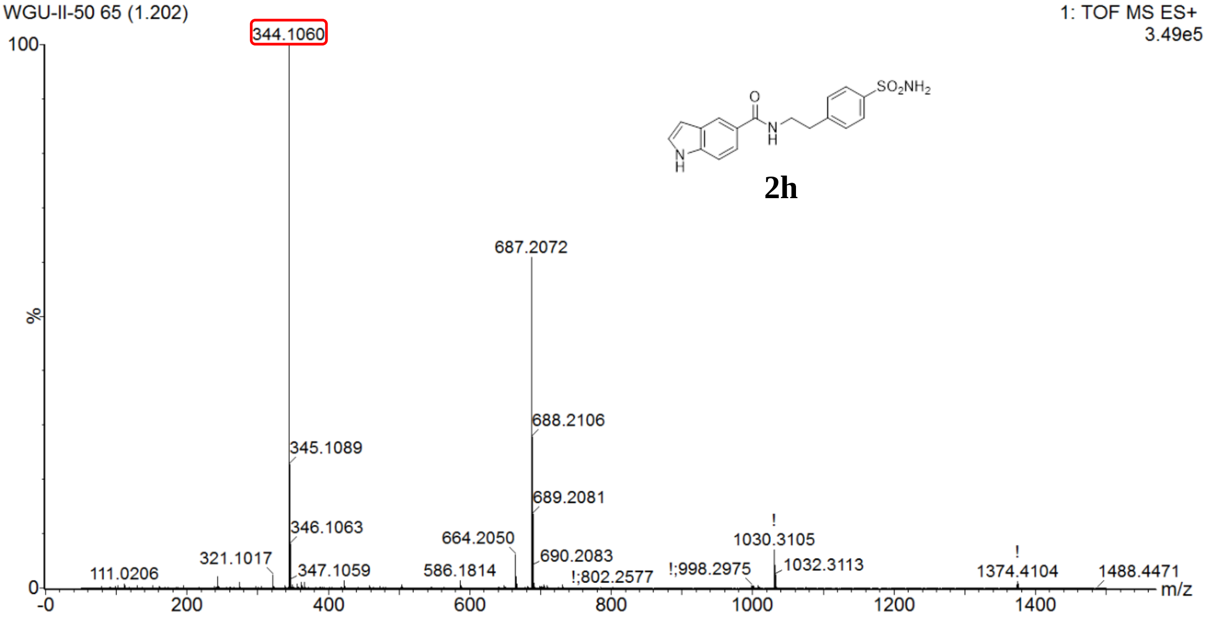

2h

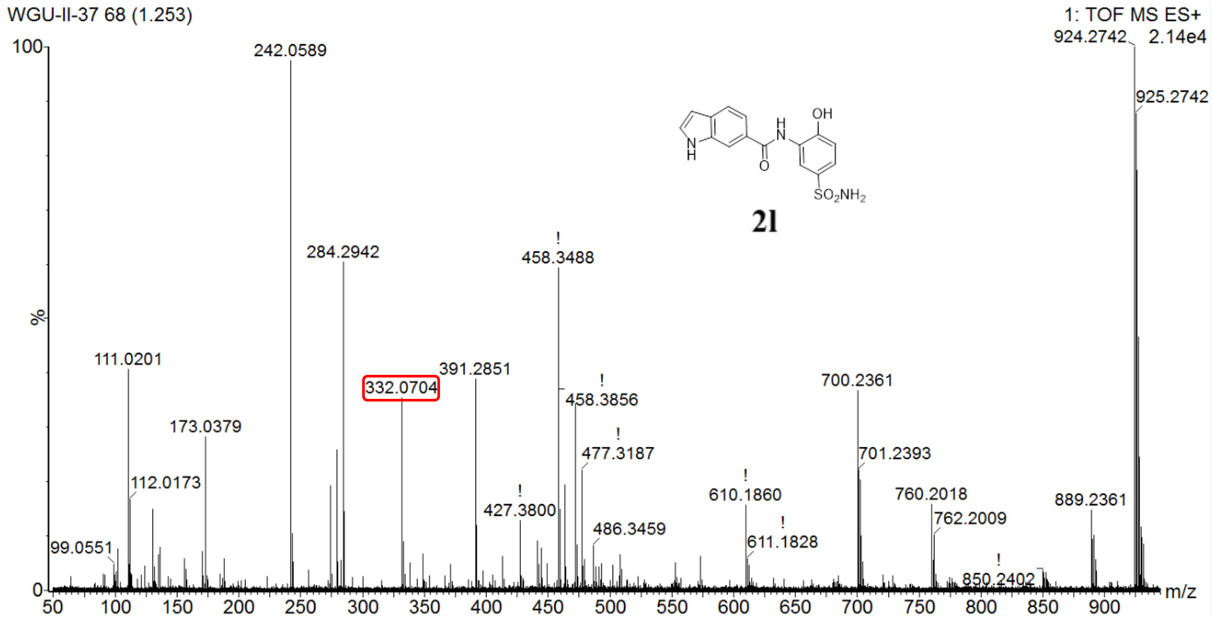

WGU-II-37 68 (1.253)
1: TOF MS ES+
2.14e4
100
%
0
242.0589
284.2942
458.3488
111.0201
332.0704
391.2851
458.3856
700.2361
173.0379
477.3187
112.0173
701.2393
427.3800
610.1860
760.2018
889.2361
99.0551
486.3459
762.2009
611.1828
850.2402
924.2742
925.2742
m/z
2l

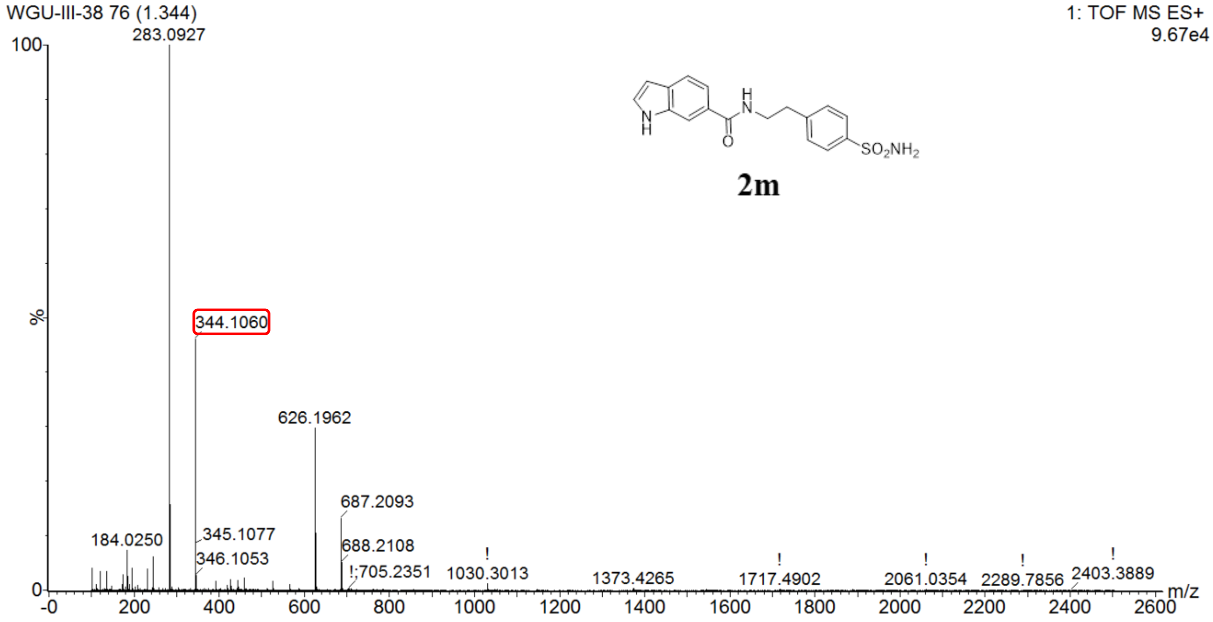

WGU-III-38 76 (1.344)
1: TOF MS ES+
9.67e4
100
%
0
283.0927
344.1060
626.1962
687.2093
184.0250
345.1077
688.2108
346.1053
705.2351
1030.3013
1373.4265
1717.4902
2061.0354
2289.7856
2403.3889
m/z
2m

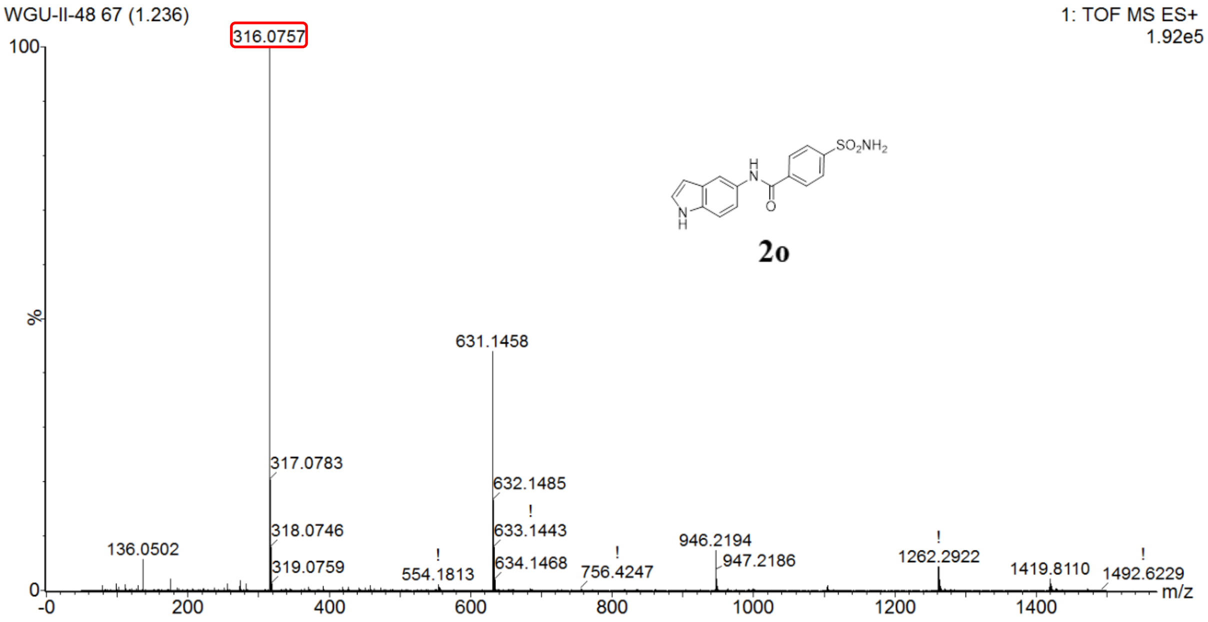
WGU-II-48 67 (1.236)
1: TOF MS ES+
1.92e5
316.0757
631.1458
317.0783
632.1485
318.0746
633.1443
136.0502
319.0759
554.1813
634.1468
756.4247
946.2194
947.2186
1262.2922
1419.8110
1492.6229
SO2NH2
2o
%
m/z
100
0
-0
200
400
600
800
1000
1200
1400

4. Interpretation of HRMS:

Compound 2c:

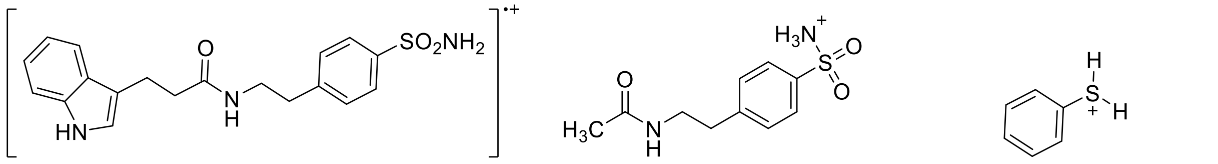

Chemical Formula: $C_{19}H_{21}N_3O_3S^{\bullet+}$ Exact Mass: 371.1298

Chemical Formula: $C_{10}H_{15}N_2O_3S^+$ Exact Mass: 243.0798

Chemical Formula: $C_6H_7S^+$ Exact Mass: 111.0263

Compound 2d:

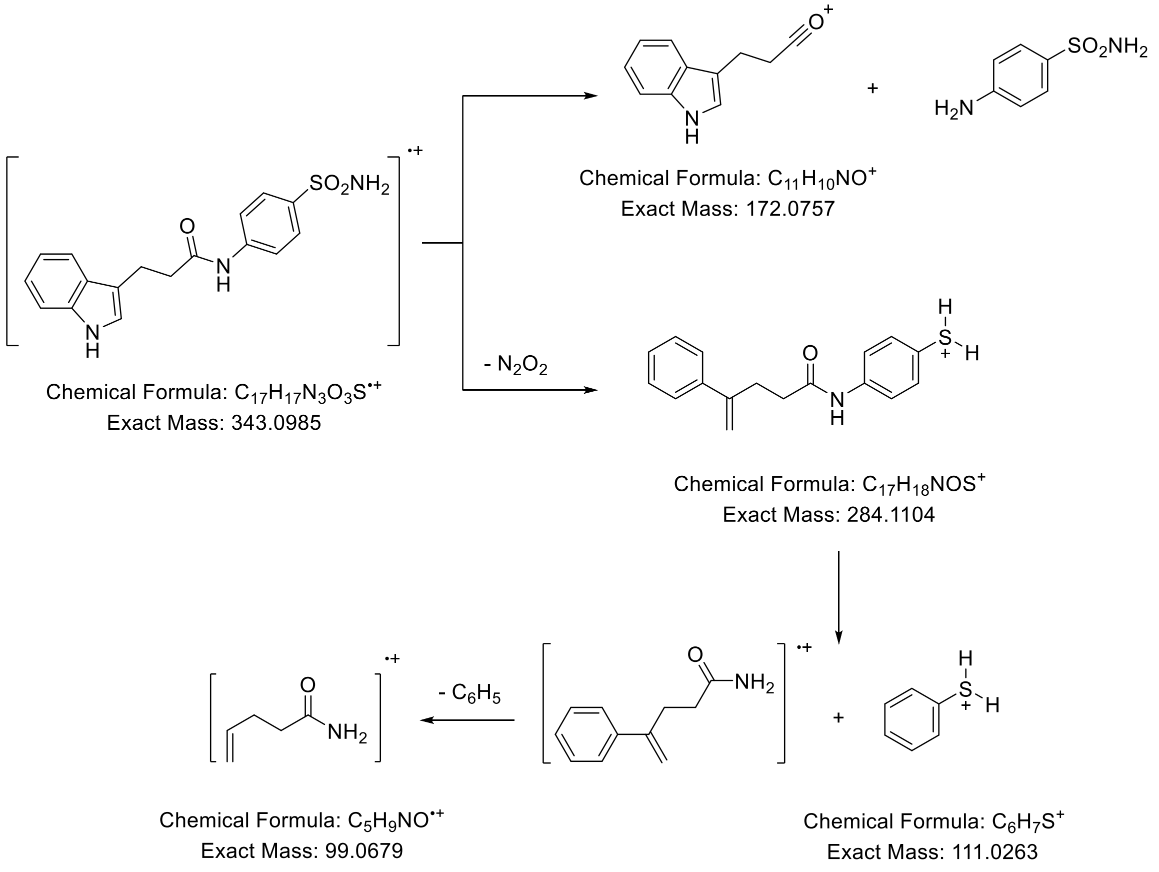

Compounds 2e and 2f:

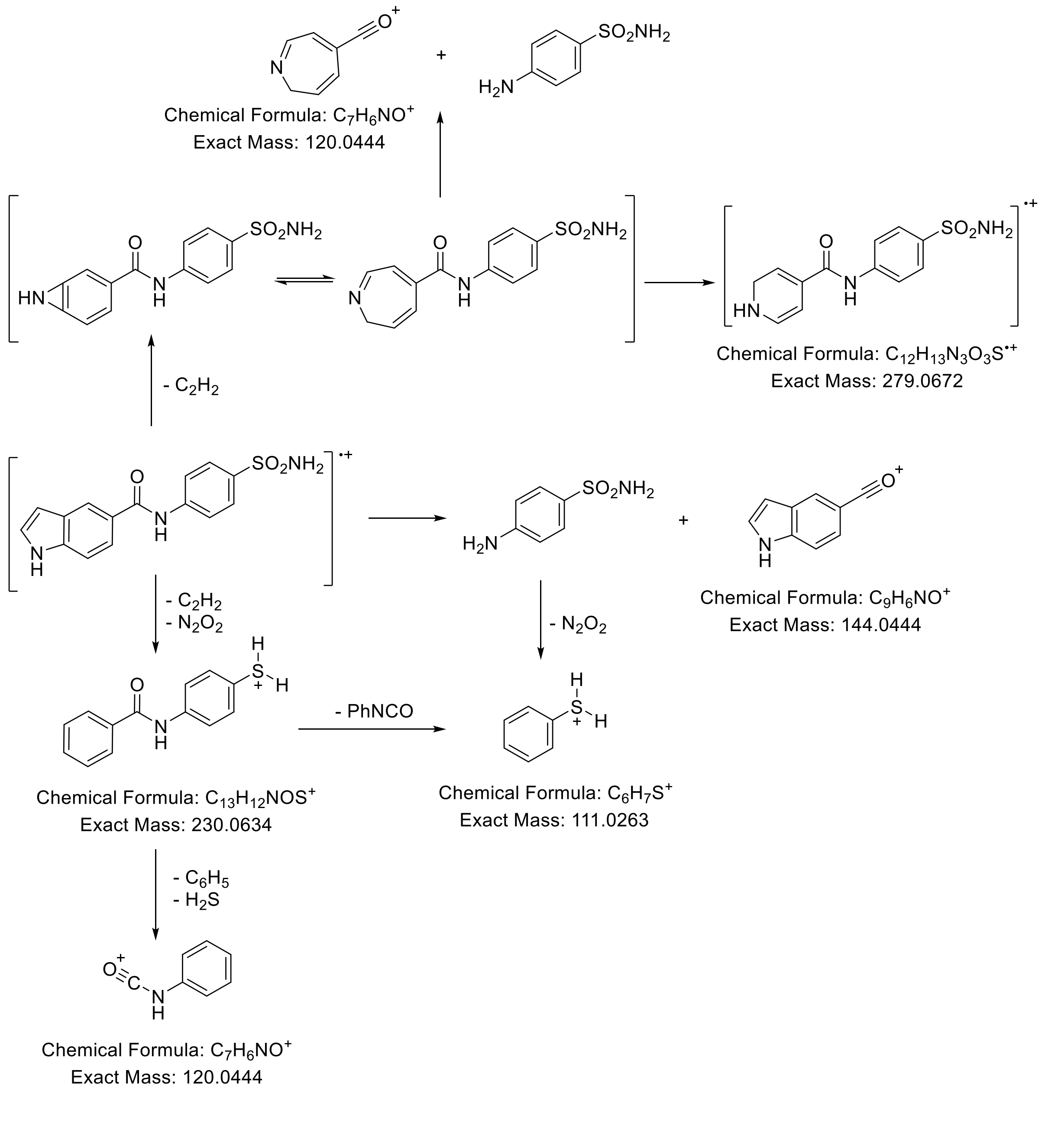
SO2NH2
H2N
Chemical Formula: C7H6NO+
Exact Mass: 120.0444
HN
N
H
Chemical Formula: C12H13N3O3S•+
Exact Mass: 279.0672
- C2H2
- C2H2
- N2O2
- N2O2
- PhNCO
Chemical Formula: C9H6NO+
Exact Mass: 144.0444
Chemical Formula: C13H12NOS+
Exact Mass: 230.0634
Chemical Formula: C6H7S+
Exact Mass: 111.0263
- C6H5
- H2S
Chemical Formula: C7H6NO+
Exact Mass: 120.0444

Compound 2h:

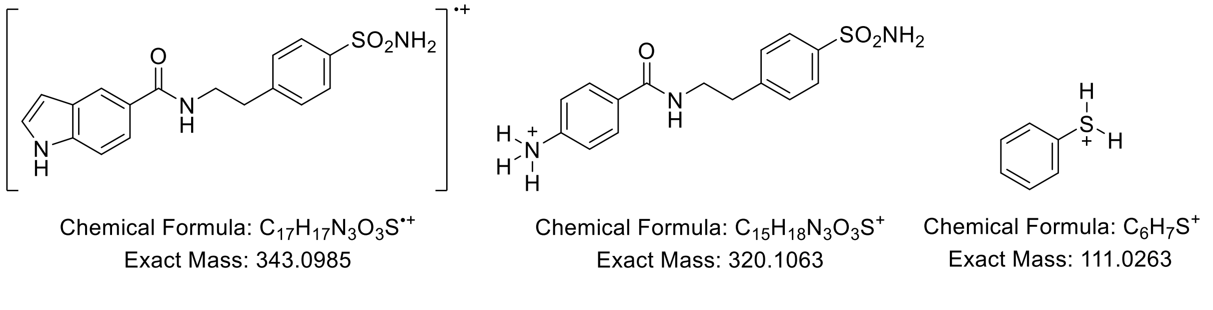
SO2NH2
Chemical Formula: C17H17N3O3S•+
Exact Mass: 343.0985
Chemical Formula: C15H18N3O3S+
Exact Mass: 320.1063
Chemical Formula: C6H7S+
Exact Mass: 111.0263
